# Mental burden and its risk and protective factors during the early phase of the SARS-CoV-2 pandemic: systematic review and meta-analyses

**DOI:** 10.1186/s12992-021-00670-y

**Published:** 2021-03-29

**Authors:** Angela M. Kunzler, Nikolaus Röthke, Lukas Günthner, Jutta Stoffers-Winterling, Oliver Tüscher, Michaela Coenen, Eva Rehfuess, Guido Schwarzer, Harald Binder, Christine Schmucker, Joerg J. Meerpohl, Klaus Lieb

**Affiliations:** 1 Department of Psychiatry and Psychotherapy, University Medical Center of the Johannes Gutenberg University Mainz, Mainz, Germany; 2 Leibniz Institute for Resilience Research (LIR), Mainz, Germany; 3 Institute for Medical Information Processing, Biometry and Epidemiology, Chair of Public Health and Health Services Research, LMU Munich, Munich, Germany; 4 Pettenkofer School of Public Health Munich, Munich, Germany; 5 Institute of Medical Biometry and Statistics, Faculty of Medicine and Medical Center, University of Freiburg, Freiburg, Germany; 6 Institute for Evidence in Medicine, Medical Center – University of Freiburg, Faculty of Medicine, University of Freiburg, Freiburg, Germany; 7 Cochrane Germany, Cochrane Germany Foundation, Freiburg, Germany

**Keywords:** SARS-CoV-2, COVID-19, Early phase, Psychological distress, Pandemic, Health personnel, Systematic review, Meta-analysis, Anxiety, Depression

## Abstract

**Background:**

Mental burden due to the SARS-CoV-2 pandemic has been widely reported for the general public and specific risk groups like healthcare workers and different patient populations. We aimed to assess its impact on mental health during the early phase by comparing pandemic with prepandemic data and to identify potential risk and protective factors.

**Methods:**

For this systematic review and meta-analyses, we systematically searched PubMed, PsycINFO, and Web of Science from January 1, 2019 to May 29, 2020, and screened reference lists of included studies. In addition, we searched PubMed and PsycINFO for prepandemic comparative data. Survey studies assessing mental burden by the SARS-CoV-2 pandemic in the general population, healthcare workers, or any patients (eg, COVID-19 patients), with a broad range of eligible mental health outcomes, and matching studies evaluating prepandemic comparative data in the same population (if available) were included. We used multilevel meta-analyses for main, subgroup, and sensitivity analyses, focusing on (perceived) stress, symptoms of anxiety and depression, and sleep-related symptoms as primary outcomes.

**Results:**

Of 2429 records retrieved, 104 were included in the review (*n* = 208,261 participants), 43 in the meta-analysis (*n* = 71,613 participants). While symptoms of anxiety (standardized mean difference [SMD] 0.40; 95% CI 0.15–0.65) and depression (SMD 0.67; 95% CI 0.07–1.27) were increased in the general population during the early phase of the pandemic compared with prepandemic conditions, mental burden was not increased in patients as well as healthcare workers, irrespective of COVID-19 patient contact. Specific outcome measures (eg, Patient Health Questionnaire) and older comparative data (published ≥5 years ago) were associated with increased mental burden. Across the three population groups, existing mental disorders, female sex, and concerns about getting infected were repeatedly reported as risk factors, while older age, a good economic situation, and education were protective.

**Conclusions:**

This meta-analysis paints a more differentiated picture of the mental health consequences in pandemic situations than previous reviews. High-quality, representative surveys, high granular longitudinal studies, and more research on protective factors are required to better understand the psychological impacts of the SARS-CoV-2 pandemic and to help design effective preventive measures and interventions that are tailored to the needs of specific population groups.

**Supplementary Information:**

The online version contains supplementary material available at 10.1186/s12992-021-00670-y.

## Introduction

The emergence of novel severe acute respiratory syndrome coronavirus 2 (SARS-CoV-2) was described for the first time in Wuhan, China [1, 2] and declared a public health emergency of international concern on 30 January 2020 [3]. The virus spread rapidly and, as of January 14, 2021, led to 90,759,370 confirmed infections and 1,963,169 deaths worldwide [4].

During the early phase of the pandemic, many countries adopted drastic measures, including testing, tracing, self-isolation, and quarantine measures as well as broader population measures ranging from travel bans, school closures, assembly restrictions, curfews, to full lockdowns [5–7]. Besides substantial stressors for individuals and the general public (eg, social isolation, reduced income, restructuring of school, university, and work life) and healthcare systems (eg, disruption of essential health services) [8, 9], the SARS-CoV-2 pandemic has had major socio-economic consequences for the affected countries (eg, global supply chain disruptions) [10, 11]. By drastically changing our way of social interaction (eg, social distancing), it continues to affect many areas of daily life and in line with this social life and participation.

The disease-related threats, containment measures, and associated stressors may have a negative psychological impact on the community at large and potentially even more so on specific risk groups [12–17]. Given the work-related stressors in the context of disease outbreaks (eg, high workload, risk of infection, triage decisions), healthcare workers may suffer from a particularly high burden [18]^1^^,^^2^ [20–22]. Patients with pre-existing physical or mental conditions (eg, chronically ill individuals, psychiatric patients, geriatric patients), people with confirmed COVID-19 diagnosis, those recovering from the infection, or suffering from long COVID-19, and subgroups with special risk exposure (eg, caregivers) may also be at risk of developing stress-related mental symptoms [15, 22–28].

Various systematic reviews have synthesized the evidence on psychiatric symptoms associated with previous highly contagious infectious disease outbreaks (eg, Ebola, SARS-CoV) and the SARS-CoV-2 pandemic [20, 24, 29–35], some of them also narratively summarizing risk and protective factors for mental health [20, 30, 31, 33, 34]. Several meta-analyses have been conducted, either calculating the pooled prevalence of mental symptoms or odds ratios for the risk of mental burden attributable to the SARS-CoV-2 pandemic [20, 24, 29, 32, 33, 36]. Potential moderators of the negative mental health impact were also partly investigated [32]. International evidence indicates an elevated level of mental symptoms in the general public, including symptoms of anxiety, depression, and stress [30–33, 36]. Confirming the risk status of healthcare workers, several reviews also found an increased prevalence of mental symptoms in this group [18]^1,2^ [20, 29, 31, 32]. Finally, a few studies in patient populations (eg, COVID-19 patients, patients with pre-existing mental or physical conditions) show increased mental burden [24, 31–33].

There are several shortcomings of reviews published to date. Most either focus on the general population, healthcare workers, or patients, with only few publications examining the level of mental burden across all three specified, most relevant population groups [31–33]. Further limitations included a limited search strategy [31], language restrictions [24, 30, 31, 33], or a missing preregistration [20, 24, 29–31, 33, 36]. Most importantly, all but one systematic review failed to compare the mental burden during an ongoing pandemic with the burden before the pandemic [31]. Such comparisons, however, are necessary to quantify the mental burden specifically attributable to the current pandemic. We therefore aimed to assess the mental health impact of the SARS-CoV-2 pandemic by comparing data from the early phase of the current pandemic with prepandemic data in the general population, healthcare workers, and patients. We aimed to identify population-specific risk and protective factors for mental health.

## Methods

### Review registration

This systematic review [37] was preregistered with PROSPERO (registration no. CRD42020193249) with the title ‘Psychological distress, protective factors and resilience during the SARS-CoV-2 pandemic: a systematic review and meta-analysis with comparison to standard data’. Details of the methods are presented in the Additional file 1. The MOOSE Checklist for Meta-analyses of Observational Studies and differences between the protocol and the final review are presented in eTables 1 and 2.

### Search strategy and selection criteria

We searched three bibliographic databases from January 1, 2019 to May 29, 2020 (PubMed, PsycINFO, and Web of Science) and inspected the reference lists of included studies. The search strategy comprised terms associated with mental health, pandemics, and the populations of interest (see eMethods 1 in Additional file 1). There were no restrictions concerning language, publication date, or publication format. We did not consider preprint articles. If not reported within a study, we systematically searched for prepandemic comparative data in the same or a similar population (PubMed, PsycINFO; see eMethods 2).

The populations of interest comprised the general population, healthcare workers, and any patients (eg, COVID-19 patients, those with pre-existing physical or mental conditions; eTable 3). Participants were included irrespective of age, health, or employment status. We did not consider infectious disease outbreaks other than due to SARS-CoV-2. To be eligible for the review, studies had to assess at least one mental health outcome, with a broad range of eligible outcomes (ie, anxiety and worrying, depression, posttraumatic stress, sleep, stress, general psychological distress). These outcomes were also considered for a descriptive synthesis of the prevalence (see data analysis). We included original research articles reporting on cross-sectional and longitudinal surveys.

All pandemic studies meeting these criteria were included but were only taken forward to pairwise meta-analyses if using a validated outcome measure and if prepandemic comparative data were available (eTables 4, 5). These were defined as data collected before the exposure to the current pandemic, and in the absence of other disease outbreaks or macro-stressors (eg, disasters), in the same country and population group (if available) and using the same outcome measure. In contrast to the review, we only focused on the four most frequently reported mental health outcomes (primary outcomes), including symptoms related to stress, anxiety, depression, or sleep. Posttraumatic stress, although reported more often than sleep, was not considered for pairwise meta-analyses. As this outcome is usually measured in the aftermath of macro-stressors, we were not able to identify adequate comparative data as mentioned above. Comparative data were selected stepwise using four levels to ensure best available comparability between SARS-CoV-2 exposure (‘pandemic’) studies and prepandemic (‘comparative’) studies. If representative studies in the same country and population (level 1) were not available, we used prepandemic studies in the same (level 2) or an alternative population (level 3; eg, healthcare workers compared with the general population), before resorting to the best available data in a similar country (level 4).

### Study selection, data extraction, and quality assessment

The study selection process for the pandemic studies at the level of titles/abstracts and full-texts was performed in duplicate by two reviewers independently (NR, LG). Any disagreements were resolved by discussion or by consulting a third reviewer (KL). At both title/abstract (κ = 0.90) and full-text level (κ = 0.97), excellent inter-rater reliability was achieved.

Relevant information for each included study was extracted in duplicate by two reviewers (NR, LG), working independently, using a customized spreadsheet (eTable 6), which was shortened for the extraction of comparative data. Discrepancies were resolved through discussion or by a third reviewer (KL).

Three independent reviewers (NR, JSW, LG) assessed the quality of included studies using the modified National Institutes of Health (NIH) Quality Assessment Tool for Observational Cohort and Cross-Sectional Studies [38] (eTable 7), with disagreements being resolved by discussion or a third reviewer (KL). The level of comparability between pandemic and comparative data was assessed using a self-developed tool with four levels based on the previously mentioned levels for the stepwise selection of comparative data (eTable 8).

### Data analysis

The included studies were synthesized in narrative and tabular form, with a descriptive analysis of prevalence rates for mental health symptoms (ie, proportion of participants beyond a cut-off score reported in the included study) and of risk and protective factors. If adequate comparative data for any of the primary outcomes were available, pairwise meta-analyses were performed for the general population, healthcare workers, and patients, respectively (eMethods 3). Given the multiple uses of comparative studies, we used multilevel meta-analyses [39] for the general population and healthcare workers, with pandemic studies being clustered according to prepandemic comparators. For patients, the multilevel model reduces to the classic random-effects model as different comparative studies were available. Prediction intervals were calculated in meta-analyses with at least four studies to take the large between-study heterogeneity into account [40].

Two sensitivity analyses referred to the quality of pandemic studies and the level of comparability (see Search strategy and selection criteria), by limiting the analyses to very comparable pandemic and prepandemic studies (ie, level 1 and 2 mentioned above).

Subgroup analyses for each of the three groups were performed for the surveyed populations (eg, age), characteristics of the pandemic studies (eg, survey start) and of comparative data (eg, publication year), and the relationship of sample sizes in pandemic versus comparative studies, in order to identify potential sources of heterogeneity of the psychological impact of the SARS-CoV-2 pandemic.

## Results

**Fig. 1:**
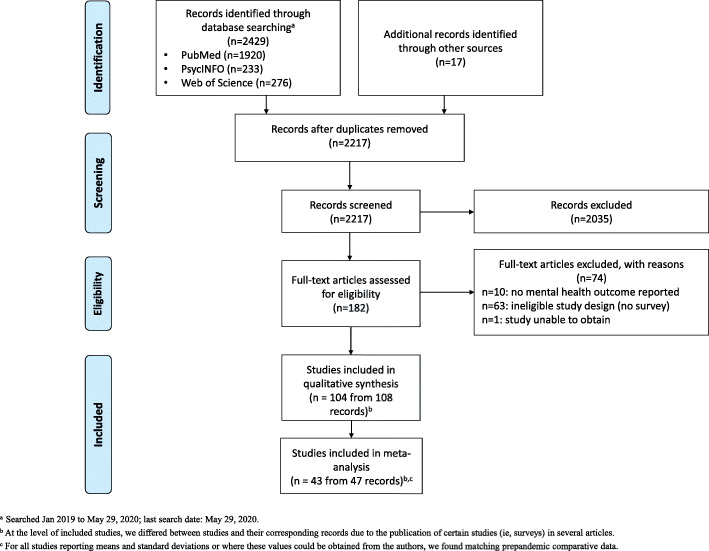
PRISMA flow diagram

Details of the results are presented in the Additional file 2. The systematic search for studies performed during the SARS-CoV-2 pandemic identified 2429 records from database searches and 17 additional records from reference lists, of which 104 studies were included in the review and 43 studies in the meta-analyses (Fig. 1). Of the 104 eligible studies, most studies were performed in the general population (50 studies), followed by 30 studies in healthcare workers, and seven studies in various patient populations. Seventeen studies included mixed samples. Across the three population groups, a total of 208,261 participants ranging from 51 to 52,730 participants [41, 42]^1^ from the pandemic studies were included in the review, the number of participants considered in the meta-analyses, in total 71,613, ranged from 127 to 60,213 participants (eTable 9).

The study characteristics of the 104 included pandemic studies (early phase) are presented in Table 1.

**Table 1:** Study characteristics of included main studies

Study	Study design	Country	Sample size; female: No. (%); age: mean (SD) or alternative information on age (eg, mode)	Subgroups	Survey period	Assessed Outcomes	Instruments or scales
**General Population**							
Ahmad et al. (2020) [43]^1^	CS, OBS	Iraq (Kurdistan)	516; 222 (43%); NA (mode: 18–35 years [65.1%])	NA	NA	Anxiety and fear	Binary single item^a^
Bacon et al. (2020) [44]^1^	CS, OBS	United Kingdom	202; 127 (62.9%), 1 diverse; 33.79 (12.48)	NA	March 18–19, 2020	Anxiety and fear	GAD-7
						Depressive symptoms	BDI-II
Bäuerle et al. (2020) [45]^1^, Teufel et al. (2020) [46]^1^	CS, OBS	Germany	15,037; 10,633 (70.7%), NA (mode: 25–34 years [24.8%])	NA	March 10–May 5, 2020	Anxiety and fear	GAD-7, single item 7-P LS^a^
						Depressive symptoms	PHQ-2
						Psychological Distress	DT
Buzzi et al. (2020) [47]^1^	CS, OBS	Italy	2064; NA; NA	100% adolescents	March 2020	Anxiety and fear	4-P LS^a^
Cao et al. (2020) [48]^1^	CS, OBS	China	7143; 4975 (69.7%); NA	NA	NA	Anxiety and fear	GAD-7
Chang et al. (2020) [49]^1^	CS, OBS	China	3881; 2447 (63.1%); 20.00 (NA); P_25=_19.00, P_75=_22.00]	100% students^b^; medical students (*n* = 3359)	January 31, 2019–February 3, 2020	Anxiety and fear	GAD-7
						Depressive symptoms	PHQ-9
Gao J et al. (2020) [50]^1^	CS, OBS	China	4872; 3267 (67.7%); 32.3 (10.0)	NA	January 31–February 02, 2020	Anxiety and fear	GAD-7
						Depressive symptoms	WHO-5^c^
Germani et al. (2020) [51]^1^	CS, OBS	Italy	1011; 720 (71.2%); 24.2 (3.6)	100% age between 18 and 29 years	March 17–24, 2020	Anxiety and fear	STAI-Y
						Stress	PSS
						Other Outcomes	SDQ
González–Sanguino et al. (2020) [52]^1^	CS, OBS	Spain	3480; 2610 (75%); 37–92 (NA)	NA	March 21–28, 2020	Anxiety and fear	GAD-2
						Depressive symptoms	PHQ-2
						PTSS	PCL-C-2
						Other outcomes	FACIT-Sp12, MSPSS, SCS
Harper et al. (2020) [53]^1^	CS, OBS	UK	324; 162 (50%); 34–32 (11.71)	NA	March 27–28, 2020	Anxiety and fear	FCV-19S, PROMIS-SF Anxiety
						Depressive symptoms	PROMIS-SF Depression
						Other outcomes	WHOQOL-BREF
Jahanshahi et al. (2020) [54]^1^	CS, OBS	Iran	1058; 569 (53–8%); NA (mode: 26–35 years)	NA	March 25–28, 2020	Psychological distress	CPDI
Lauri Korajlija et al. (2020) [55]^1^	CS (repeated), OBS	Croatia	sample 1: 888; 738^d^ (83–1%); 31.3 (10.45) sample 2: 966; 732^d^ (75.8%); 40 (11.94)	NA	1st period: February 24–NA 2nd period: March 19–NA	Anxiety and fear	11-items 5-P LS (based on Swine Flu Anxiety Items, Wheaton et al. 2012)^a^
Lee SA et al. (2020) [56]^1^	CS, OBS	USA	398; 191 (49%); 35.91 (11.73)	NA	March 23–24, 2020	Anxiety and fear	2 single items 5-P LS^a^
						Other outcomes	Passive suicidal ideation (single item 5-P LS)^a^
Lei et al. (2020) [57]^1^	CS, OBS	China	1593; 976 (61.3%); 32.3 (9.8)	‘affected group’: quarantined / relatives quarantined (*n* = 420)^b^	February 04–10, 2020	Anxiety and fear	SAS
						Depressive symptoms	SDS
Li Y et al. (2020) [58]^1^	CS (part of longitudinal cohort study), OBS	China	1442; 891^d^ (61.8%); NA (K-6 < 5: 20.0 [1.5]; K-6 ≥ 5: 20.0 [1.6])	medical students (*n* = 764), nursing students (*n* = 211), medical technology students (*n* = 467)	February 7–13, 2020	PTSS	IES-R
						Psychological distress	K-6
Liu N et al. (2020) [59]^1,2^	CS, OBS	China	285; 155 (54.4%); NA (47.7% < 35)	NA	January 30– February 08, 2020	PTSS	PCL-5
Liu S et al. (2020) [60]^1^	CS, OBS	China	primary school: 209; 116 (56%^d^); NA college: 198; 130 (62%); NA	primary school students, college students	February–March, 2020	Anxiety and fear	3 items, 4-P LS^a^
						Other outcomes	SSS
Lopez et al. (2020) [61]^1^	CS, OBS	Spain	878; 544^d^ (62%) or 636 (72%^d^), data in text and Table 1 inconsistent; NA (mode: 60–70 years [71%^d^])	100% community-dwelling older adults; age 60–70 (*n* = 626); age 71–80 (*n* = 252)	NA	Anxiety and fear	^a^
						Other outcomes	BRCS, Ryff’s PWB (subscales for personal growth and purpose in life)
Ma et al. (2020) [62]^1^	CS, OBS	China	123; 71^d^ (57.7%^d^); 37.4 (10.6)	100% isolated people^b^	January 2020	Anxiety and fear	DASS-21 Anxiety
						Depressive symptoms	DASS-21 Depression
						Stress	DASS-21 Stress
						Sleep-related symptoms	PSQI
						Other outcomes	SF-36
Mazza et al. (2020) [63]^1^	CS, OBS	Italy	2766; 1982 (71.7%); 32.94 (13.2)	NA	March 18–22, 2020	Anxiety and fear	DASS-21 Anxiety
						Depressive symptoms	DASS-21 Depression
						Stress	DASS-21 Stress
McKay et al. (2020) [64]^1^	CS, OBS	China	908; 752 (82.8%); 40.37 (9.27)	NA	February 24–March 15, 2020	Anxiety and fear	CoVGAD-7, DASS-21 Anxiety
						Depressive symptoms	DASS-21 Depression
Moccia et al. (2020) [65]^1^	CS, OBS	Italy	500; 298 (59.6); NA (mode: 28–37 years, *n* = 129)	NA	April 10–13, 2020	Psychological distress	K-10
						Other outcomes	TEMPS-A
Odriozola-González et al. (2020) [66]^1^	CS, OBS	Spain	2530; 1672 (66.1%); 27.9 (12.4)	students (*n* = 1944); administrative staff (*n* = 247); faculty members and academic staff (*n* = 339)^b^	March 28–April 3, 2020	Anxiety and fear	DASS-21 Anxiety
						Depressive symptoms	DASS-21 Depression
						Stress	DASS-21 Stress
						PTSS	IES
Olagoke et al. (2020) [146]^1^	CS, OBS	USA	501; 277 (55.29%); 32.44 (11.94)	NA	March 25, 2020–NA	Depressive symptoms	PHQ-2
						Other outcomes	Perceived self-efficacy (Ajzen 2002)
Ozamiz-Etxebarria et al. (2020) [68]^1^	CS, OBS	Spain	976; 792 (81.1%); NA (mode: 18–25 years [56.5%])	NA	March 11–15, 2020	Anxiety and fear	DASS-21 Anxiety
						Depressive symptoms	DASS-21 Depression
						Stress	DASS-21 Stress
Özdin et al. (2020) [69]^1^	CS, OBS	Turkey	343; 169 (49.2%); 37.2 (10.3)	NA	April 14–16, 2020	Anxiety and fear	HAI
						Depressive symptoms	HADS
Perez–Fuentes et al. (2020) [70]^1^	CS, OBS	Spain	1014; 681 (67.2%); 40.87 (12.42)	NA	March 18–23, 2020	Depressive symptoms	BIP-Q5
Qiu et al. (2020) [41]^1^	CS, OBS	China, Hong Kong, Macao, Taiwan	52,730; 34,131 (64.7%)	NA	January 31–February 2, 2020	Psychological distress	CPDI
Ren et al. (2020) [71]^1^	CS, OBS	China	1172; NA; NA	NA	February 14–March 29, 2020	Anxiety and fear	GAD-7
						Depressive symptoms	PHQ-9
						Stress	PSS-10
						Sleep-related symptoms	ISI
						PTSS	PCL-5
						Other outcomes	MINI suicidality module
Reznik et al. (2020) [72]^1^	CS, OBS	Russia & Belarus	850; 622 (73.2%); 34.8 (13.0)	NA	after March 27, 2020	Anxiety and fear	FCV-19S
Roy et al. (2020) [73]^1,2^	CS, OBS	India	662; 339 (51.2%); 29.09 (8.83)	NA	March 22–24, 2020	Anxiety and fear	18 items 5-P LS^a^
Sakib et al. (2020) [74]^1^	CS, OBS	Bangladesh	8550; 3760 (44%); 26.5 (9.1)	NA	April 1–10, 2020	Anxiety and fear	FCV-19S
						Depressive symptoms	PHQ-9
Satici et al. (2020) [75]^1^	CS, OBS	Turkey	1304; 917 (70.3%); 29.5 (10.5)	NA	NA	Anxiety and fear	DASS-21 Anxiety, FCV-19S
						Depressive symptoms	DASS-21 Depression
						Stress	DASS-21 Stress
Shammi et al. (2020) [76]^1^	CS, OBS	Bangladesh	1066; 405 (38.5%); 27.80 (10.05)	NA	March 28–30, 2020	Psychological distress	COVID-19 related mental distress (5 items 5-P LS)^a^
Shevlin et al. (2020) [77]^1^	CS, OBS	UK	2025; 1047 (51.9%); 45.4 (15.9)	NA	March 23–28, 2020	Anxiety and fear	GAD-7, VAS on COVID-19 anxiety
						Other outcomes	PHQ-15
Soraci et al. (2020) [78]^1^	CS, OBS	Italy	249; 229 (92%); 34.50 (12.21)	NA	March 18–21, 2020	Anxiety and fear	FCV-19S, HADS
Sutin et al. (2020) [147]^1^	CS, OBS	USA	2094; 1024 (48.9%)^d^; 51.03 (16.58)	overweight (*n* = 706); obesity (*n* = 587)	mid–March, 2020	Anxiety and fear	13 items 5-P LS^a^
Tan W et al. (2020) [80]^1^	CS, OBS	China	673; 172^d^ (25.6%^d^); 30.8 (7.4)	NA	February 24–252,020	Anxiety and fear	DASS-21 Anxiety
						Depressive symptoms	DASS-21 Depression
						Stress	DASS-21 Stress
						Sleep-related symptoms	ISI
						PTSS	IES-R
Tian et al. (2020) [81]^1^	CS, OBS	China	1060; 511 (48.2%); 35.01 (12.8)	HCW (n = 42), students (*n* = 330)	January 31–February 02, 2020	Anxiety and fear	SCL-90 Anxiety
						Depressive symptoms	SCL-90 Depression
						Psychological distress	SCL-90 GSI
						Other outcomes	SCL-90 subscales
Tsipropoulou et al. (2020) [82]^1^	CS, OBS	Greece	2970; 2153 (72.5%); NA (mode: 18–30 years [52%])	NA	NA	Anxiety and fear	FCV-19S, GAD-7
						Depressive symptoms	PHQ-9
Tull et al. (2020) [79]^1^	CS, OBS	USA	500; 235^d^ (47%); 40 (11.6)	NA	March 27–April 5, 2020	Anxiety and fear	DASS-21 Anxiety, SHAI
						Depressive symptoms	DASS-21 Depression
						Stress	DASS-21 Stress
Voitsidis et al. (2020) [83]^1^	CS, OBS	Greece	2363; 1800 (76.2%); NA (mode: 18–30 years [55%])	NA	April 10–13, 2020	Anxiety and fear	^a^
						Depressive symptoms	PHQ-2
						Sleep-related symptoms	AIS
						Other outcomes	IUS-12, JGLS
Wang C et al. (2020a) [84]^1,2^, Wang C et al. (2020b) [85]^1^	2 CS (repeated), OBS	China	1738 not counting participants in both surveys; 333 in both 1st survey: 1210; 814^d^ or 878^d^ (67.3%); NA (mode: 21.4–30.8 years [53.1%]) 2nd survey: 861; 646^d^ (75%); NA (mode: 21.4–30.8 years [46.5%])	NA	January 31–February 2, 2020 and February 28–March 1, 2020	Anxiety and fear	DASS-21 Anxiety
						Depressive symptoms	DASS-21 Depression
						Stress	DASS-21 Stress
						PTSS	IES-R
Wang H et al. (2020) [86]^1^	CS, OBS	China	1599; 1068 (66.8%); 33.9 (12.3)	NA	February 1–4, 2020	Psychological distress	K-6
Wang Y et al. (2020) [87]^1,2^	CS, OBS	China	600; 333 (55.5%); 34 (12)	NA	February 6–9, 2020	Anxiety	SAS
						Depressive symptoms	SDS
Yang H et al. (2020) [88]^1^	CS (repeated), OBS	China	during COVID-19: 3000; 1500^d^ (50%); 34.7 (NA)	NA	end of December 2019 and mid–February, 2020	Other outcomes	Emotional well-being (Kahneman and Deaton, 2010)
Yuan R et al. (2020) [89]^1^	CS, OBS	China	parents of children hospitalised during the epidemic (EH): 50; 31 (62%^d^); 36.80 (5.20) parents of children hospitalised during the non-epidemic period (NEH): 50; 26 (52%^d^); 37.22 (5.40)	EH (*n* = 50)^b^, NEH (n = 50)^b^	NA	Anxiety	HADS Anxiety, VDAS
						Depressive symptoms	HADS Depression
						Other Outcomes	SF-36
Zhang SX et al. (2020a) [90]^1^; Zhang SX et al. (2020b) [91]^1^	CS, OBS	China	369; 165 (44.7%); 36.6 (10.5)	NA	February 20–21, 2020	Psychological Distress	K6
						Other outcomes	SF12, SWLS
Zhang Y et al. (2020) [92]^1,2^	CS, OBS	China	263; 157 (60%); 37.7 (14.0)	NA	January 28–February 05, 2020	PTSS	IES
Zhou SJ et al. (2020) [93]^1^	CS, OBS	China	8079; 4326 (53.5%); NA (median: 16, minimum 12, maximum 18 years)	100% senior high school students^b^	March 8–15, 2020	Anxiety	GAD-7
						Depressive symptoms	PHQ-9
**Healthcare workers**							
Abdessater et al. (2020) [94]^1^	CS, OBS	France	275; 91^d^ (33%) or 83^d^ (30%), ambiguous data; 29.5 (0.47)	100% urologists	March 27–30, 2020	Stress	^a^
Ahmed et al. (2020) [95]^1^	CS, OBS	multinational (Pakistan > Saudi Arabia > others)	650; 490 (75%); NA (mode: 20–30 years [54%])	100% dentists	March 10–17, 2020	Anxiety	8 binary items^a^
Alhaj et al. (2020) [96]^1^	CS, OBS	multinational (Canada, USA, others)	52; 14 (27%); NA (mode: < 30 years [69%])	100% surgeons	April 14–28, 2020	Psychological distress	Affection of mental health (binary single item)^a^
Amerio et al. (2020) [97]^1^	CS, OBS	Italy	131; 63 (48.1%); 52.3 (12.2)	100% physicians (general practitioners)	March 15–April 15, 2020	Anxiety	GAD-7
						Depressive symptoms	PHQ-9
						Sleep-related symptoms	ISI
						Other outcomes	SF-12
Badahdah et al. (2020) [98]^1^	CS, OBS	Oman	194; 116^d^ (60%); 40.72 (8.53)	100% physicians	early April 2020	Anxiety	GAD-7
						Stress	PSS-10
						Other outcomes	WHO-5^c^
Bohlken et al. (2020) [99]^1^	CS, OBS	Germany	396; NA; 165 (42%); 56.9 (7.6)	100% physicians	April 1–6, 2020	Anxiety and fear	Single items 5-P LS^a^
						Sleep disorders	Single item 5-P LS^a^
Cai H et al. (2020) [100]^1,2^	CS, OBS	China	534; 367 (69%); 36.4 (16.18)	physicians (*n* = 233), nurses (*n* = 248)	January–March, 2020	Anxiety and fear	Single items 4-P LS^a^
Cai W et al. (2020) [101]^1^	CS, OBS	China	whole sample: 1521; 1149 (75.5%^d^); NA (mode: 18–30 years, [43.5%])	physicians (*n* = 511), nurses (*n* = 546)	NA	Anxiety and fear	SCL-90 anxiety
						Depressive symptoms	SCL-90 depression
						Psychological distress	SCL-90 positive items
						Other outcomes	SCL-90 subscales, CD-RISC, SSRS
Chew et al. (2020) [102]^1^	CS, OBS	multinational (Singapore, India)	906; 583 (64.3%); NA (median [IQR]: 29 [25–35] years)	physicians (*n* = 268), nurses (*n* = 355), allied healthcare professionals (*n* = 96), non-HCW (*n* = 187)	February 19–April 17, 2020	Anxiety and fear	DASS-21 anxiety
						Depressive symptoms	DASS-21 depression
						Stress	DASS-21 stress
						Sleep-related symptoms	Single item 4-P LS^a^
						PTSS	IES-R
Consolo et al. (2020) [103]^1^	CS, OBS	Italy	356; 141 (39.6%); NA (mode: 35–55 years [48.6%])	100% dentists	April 2–21, 2020	Anxiety and fear	GAD-7
Gan et al. (2020) [104]^1^	CS, OBS	China	11,183; 10,811 (96.7%); NA (mode: 20–29 years)	100% nurses	February 4–10, 2020	Anxiety and fear	VAS on anxiety
						Stress	VAS on stress
Huang JZ et al. (2020) [105]^1,2^	CS, OBS	China	230; 187 (81.3%); NA (mode: 30–39 years [53%])	physicians (n = 70), nurses (*n* = 160)	February 7–14, 2020	Anxiety and fear	SAS
						PTSS	PTSD-SS
Kang et al. (2020) [106]^1,2^	CS, OBS	China	994; 850 (85.5%); NA (mode: 30–40 years [63.4%])	physicians (*n* = 183), nurses (*n* = 811)	January 29–February 4, 2020	Anxiety and fear	GAD-7^e^
						Depressive symptoms	PHQ-9^e^
						Sleep-related symptoms	ISI^e^
						PTSS	IES-R^e^
Khusid et al. (2020) [107]^1^	CS, OBS	USA	332; 117 (35%); 30.5 (2.6)	100% urologists	April 7–11, 2020	Anxiety and fear	2 items 5-P LS^a^
						Depressive symptoms	2 items 5-P LS^a^
Lai et al. (2020) [18]^1,2^	CS, OBS	China	1257; 964 (76.7%); NA (mode: 26–40 years [64.7%])	physicians (*n* = 493), nurses (n = 764)	January 29–February 3, 2020	Anxiety and fear	GAD-7
						Depressive symptoms	PHQ-9
						Sleep-related symptoms	ISI
						PTSS	IES
Mo et al. (2020) [108]^1,2^	CS, OBS	China	180; 162 (90%); 32.71 (6.52)	NA	end of February 2020	Anxiety and fear	SAS
						Stress	SOS
Pu et al. (2020) [109]^1^	CS, OBS	China	867: 829 (95.6%^d^); 30.8 (7.1)	100% nurses	NA	Anxiety and fear	SAS
						Other outcomes	TAF
Rossi et al. (2020) [110]^1^	CS, OBS	Italy	1379; 1064 (77.2%); 39.0 (6.0)	physicians (*n* = 433), general practitioners (*n* = 86), nurses (*n* = 472)	March 27–31, 2020	Anxiety and fear	GAD-7
						Depressive symptoms	PHQ-9
						Stress	PSS
						Sleep-related symptoms	ISI
						PTSS	GPS–PTSD
Sahu et al. (2020) [111]^1^	CS, OBS	India	611; NA; NA (mode: 30–40 years, *n* = 192 [31·4%])	100% orthopedic surgeons	March 31–April 4, 2020	Stress	Single-item^a^
Shacham et al. (2020) [112]^1^	CS, OBS	Israel	338; 198 (586%); 46.39 (11.2)	dentists (*n* = 198), dental hygienists (*n* = 140^d^)	March 30–April 10, 2020	Psychological distress	K-6
Suleiman et al. (2020) [113]^1^	CS, OBS	Jordan	308; 113 (36.7%); 30.3 (5.8)	100% physicians	March 23–27, 2020	Anxiety and fear	Binary single items^a^
Tan B et al. (2020) [114]^1^	CS, OBS	Singapore	470; 321 (68.3%); NA (median: 31, IQR: 28–36 years)	physicians (*n* = 135), nurses (*n* = 161), allied hospital personnel (*n* = 174)	February 19–March 13, 2020	Anxiety and fear	DASS-21 anxiety
						Depressive symptoms	DASS-21 depression
						Stress	DASS-21 stress
						PTSS	IES-R
Wang S et al. (2020) [115]^1^	CS, OBS	China	123; 111 (90%); 33.75 (8.41)	100% pediatricians; physicians (*n* = 48), nurses (*n* = 75)	January 30–February 07, 2020	Anxiety and fear	SAS
						Depressive symptoms	SDS
						Sleep-related symptoms	PSQI
Wu K et al. (2020) [116]^1^	CS, OBS, controlled	China	experimental group: 60; 44 (73%); 33.5 (12.4) comparison group: 60; 45 (75%) 33.8 (11.9)	COVID-19 hospital (*n* = 60), non-designated hospital = comparison group (n = 60)	NA	Anxiety and fear	SAS, SCL-90 anxiety
						Depressive symptoms	SCL-90 depression, SDS
						Sleep-related symptoms	PSQI
						PTSS	PCL-C
						Psychological distress	SCL-90 total score
						Other outcomes	SCL-90 subscales
Xiao et al. (2020a) [117]^1,2^	CS, OBS	China	180; 129 (71.7%); 32.31 (4.88)	physicians (*n* = 82), nurses (*n* = 98)	January–February, 2020	Anxiety and fear	SAS
						Sleep-related symptoms	PSQI
						Other outcomes	GSES, SASR, SSRS
Xu J et al. (2020) [118]^1^	CS, OBS, controlled	China	outbreak period: 60; 38 (63.3%); 36.68 (9.67) ‘post-epidemic’: 60; 32 (53.3%); 35.77 (7.06)	100% surgeons	January 28–February 29, 2020 and March 2–21, 2020	Anxiety and fear	‘Anxiety scale’, dream anxiety score
						Depressive symptoms	‘Depression score’
						Other outcomes	SF-36
Yin et al. (2020) [119]^1^	CS, OBS	China	371; 228 (61.5%); 35.3 (9.5) physicians: NA nurses: NA	physicians (*n* = 67), nurses (*n* = 264)	February 01–05, 2020	Sleep-related symptoms	PSQI
						PTSS	PCL-5
Zhang C et al. (2020) [120]^1^	CS, OBS	China	1563; 1293 (83%^d^); NA (mode: 26–40 years, *n* = 495 [31.7%^d^]) physicians: NA nurses: NA	physicians (*n* = 454), nurses (*n* = 984), administrative staff (*n* = 30), other medical staff (*n* = 95)	January 29–February 03, 2020	Anxiety and fear	GAD-7
						Depressive symptoms	PHQ-9
						Sleep-related symptoms	ISI
						PTSS	IES-R
Zhang SX et al. (2020c) [121]^1^	CS, OBS	Iran	304; 178 (58.6%); 35.1 (9.1)	NA	April 5–20, 2020	Anxiety and fear	GAD-2^d^
						Depressive symptoms	PHQ-2^d^
						Psychological distress	K6
						Other outcomes	SF-12
Zhu J et al. (2020) [122]^1^	CS, OBS	China	156; 137 (83%); 34.16 (8.06) physicians: 79; 51^d^ (65%^d^)	physicians (*n* = 79), nurses (n = 86)	February 1–29, 2020	Anxiety and fear	SAS
						Depressive symptoms	SDS
**Patients**							
Cai X et al. (2020) [123]^1^, Yuan B et al. (2020) [124]^1^	CS, OBS	China	126; 66 (52.4%); 45.7 (14.0)	100% cured COVID-19 patients	March 2–12, 2020	Anxiety and fear	SAS
						Depressive symptoms	SDS
						PTSS	PTSD-SS
Durankus et al. (2020) [125]^1^	CS, OBS	Turkey	260; 260 (100%); 29.6 (3.8)	100% pregnant women	NA	Anxiety and fear	BAI
						Depressive symptoms	EPDS, BDI
						Psychological distress	Single item 11-P LS^a^
Li X et al. (2020) [126]^1^	CS, OBS	China	76; 35 (46%); 36 (15)	suspected COVID-19 patients	January 31–February 22, 2020	Anxiety and fear	HAMA
						Depressive symptoms	HAMD
Liu X et al. (2020a) [42]^1^	CS, OBS	China	COVID-19 suspected patients: 21; 12 (57.1%); 43.1 (2.6): not COVID-19 suspected patients: 30; 15 (50%); 45.0 (9.2)	100% schizophrenia patients; COVID-19 suspected patients (n = 21), not COVID-19 suspected patients (n = 30)	January 30–February 21, 2020	Anxiety and fear	HAMA
						Depressive symptoms	HAMD
						Stress	PSS
						Sleep-related symptoms	PSQI
						Other outcomes	PANSS
Wu Y et al. (2020) [127]^1,3^	CS, OBS, controlled	China	4124; 4124 (100%^d^), NA (median: 30, range = 17–32 years)	100% pregnant women; before (group 1: *n* = 2839)/after (group 2: *n* = 1284) January 20, 2020	January 1–February 9, 2020	Anxiety and fear	EPDS-3A
						Depressive symptoms	EPDS
Xu H et al. (2020) [128]^1^	CS, OBS	China	350; 199 (54.1%); NA (mode: 40–60 years [51%])	100% lung cancer patients	March 4–6, 2020	Depressive symptoms	Single item^a^
						Sleep-related symptoms	Single item^a^
Yassa et al. (2020) [129]^1^	CS, OBS	Turkey	172; 172 (100%); 27.5 (5.3)	100% pregnant women	ten days after first confirmed COVID-19 death in Turkey	Anxiety and fear	Single ternary item^a^
**Mixed groups**							
Büntzel et al. (2020) [130]^1^	CS, OBS	Germany	193; NA; NA (mode: > 60 years)	physicians (n = 47), cancer patients (*n* = 146)	April 16–19, 2020	Anxiety and fear	Single item^a^
						Stress	Single item^a^
Guo et al. (2020) [131]^1^	CS, OBS, controlled	China	P:103; 44 (42.7%); 42.5 (12.5); control (GP): 103; 49 (47.6%); 41.5 (13.1)	COVID-19 patients (*n* = 103), not infected control group (n = 103)	February 10–28, 2020	Anxiety and fear	GAD-7
						Depressive symptoms	PHQ-9
						Stress	PSS-10
						PTSS	PCL-5
Hao F et al. (2020) [132]^1^	CS, OBS, controlled	China	P: 76; 51 (37.1%); 32.8 (11.8); control (GP): 109; 68 (62.4%); 33.1 (11.2)	psychiatric patients (n = 76), control group (*n* = 109)	February 19–22, 2020	Anxiety and fear	DASS-21 anxiety
						Depressive symptoms	DASS-21 depression
						Stress	DASS-21 stress
						Sleep-related symptoms	ISI
						PTSS	IES-R
Hao X et al. (2020) [133]^1^	CS, OBS, controlled	China	P: 252; 132^d^ (52.4%^d^); 29.3 (11.6); control (GP): 252; 132^d^ (52.4%^d^); 29.4 (11.5)	epilepsy patients (n = 252), control group (n = 252)	February 1–29, 2020	Psychological distress	K-6
Huang Y et al. (2020) [134]^1,2^	CS, OBS	China	7236; 3952 (54.6%); 35.3 (5.6)	GP (*n* = 4986), HCW (*n* = 2250)	February 3–17, 2020	Anxiety and fear	GAD-7
						Depressive symptoms	CES-D
						Sleep-related symptoms	PSQI
Iasevoli et al. (2020) [135]^1^	CS, OBS, controlled	Italy	461; NA; NA P: 205; NA; NA caregivers: 51; NA; NA control (GP): 205; NA; NA	psychiatric patients (*n* = 205), caregivers (n = 51), non-psychiatric persons (n = 205)	April 13–17, 2020	Anxiety and fear	GAD-7
						Depressive symptoms	PHQ-9
						Stress	PSS
						Other outcomes	SPEQ
Jin YH et al. (2020) [136]^1^	CS, OBS	China	103; 64 (62.1%); NA (median [IQR]: 35 [14.0])	100% infected with SARS-CoV-2; physicians, nurses	February 15–29, 2020	Anxiety and fear	Single item multiple choice^a^
Ko et al. (2020) [137]^1^	CS, OBS	Taiwan	1904; 1282 (67.3%); 38.0 (10.8)	GP (n = NA), HCW (n = NA)	April 10–20, 2020	Other outcomes	Psychological wellbeing (single item 5-P LS)^a^
Li Z et al. (2020) [138]^1,2^	CS, OBS	China	740; 128 (59.8%); 25 (IQR: 22–38.3 years]	GP (n = 214), HCW (*n* = 526)	February 17–21, 2020	PTSS	Vicarious Traumatization Questionnaire
Lu W et al. (2020) [139]^1,2^	CS, OBS	China	2299; 1785 (77.6%); NA (78% < 40 years)	HCW (*n* = 2042), GP (*n* = 257)	February 25.26, 2020	Anxiety and fear	HAMA, NRS on fear
						Depressive symptoms	HAMD
Ni et al. (2020) [140]^1^	CS, OBS	China	total: 1791; NA; NA GP: 1577; 1218 ^d^ (60.8%); NA (mode: 18–34 years [38.6%]) HCW: 214; 147^d^ (68.8%); NA (mode: 18–34 years [58.9%])	GP (*n* = 1577), HCW (*n* = 214)	February 18.24, 2020	Anxiety and fear	GAD-2
						Depressive symptoms	PHQ-2
Sanchez et al. (2020) [67]^1^	CS, OBS	USA	1051; 0 (0%); 35 (15.83)	100% men who have sex with men; HIV-patients (*n* = 122)	April 2–13, 2020	Anxiety and fear	Single item^a^
						Other outcomes	Quality of life (single item)^a^
Wu W et al. (2020) [141]^1^	CS, OBS	China	4268; 2930^d^ (68.7%^d^); NA HCW: 2110; 1598^d^ (76%^d^); NA Students: 2158; 1332 (62%); NA	students (*n* = 2158), HCW (*n* = 2110)	February 10–21, 2020	Anxiety and fear	Single item^a^
						Sleep-related symptoms	Single item^a^
Yuan S et al. (2020) [142]^1,2^	L, OBS	China	939; 582 (61.98%); NA (mode: 18–39 years [71.5%])	HCW (*n* = 249), students (*n* = 312)	2 survey periods in February, 2020	Sleep-related symptoms	PSQI
						Other outcomes	SRQ
Zhang J et al. (2020) [143]^1^	CS, OBS	China	205; 115 (56.1%^d^); NA (for infected: 46.9 [15.4]; for quarantined: 36.2 [10.9]; for general public: 29.6 [12.7])	P, infected (*n* = 57), GP, quarantined (n = 50), GP, general public (n = 98)	February 15–29, 2020	Anxiety and fear	GAD-7
						Depressive symptoms	PHQ-9
Zhang WR et al. (2020) [144]^1,2^	CS, OBS	China	2182; 1401 (64.2%); NA (mode: 18–60 years [96.3%])	HCW (*n* = 927), GP (*n* = 1255)	February 19–March 6, 2020	Anxiety and fear	GAD-2
						Depressive symptoms	PHQ-2
						Sleep-related symptoms	ISI
						Other outcomes	SCL-90-R subscales
Zhu S et al. (2020) [145]^1^	CS, OBS	China	2279^d^; 1361 ^d^; NA	HCW (*n* = 858), GP (*n* = 1421)	Feb 12–Mar 17, 2020	Anxiety and fear	GAD-7
						Depressive symptoms	PHQ-9
						Psychological distress	SRQ-20

Abbreviations: *AIS* Athens Insomnia Scale, *BAI* Beck Anxiety Inventory, *BDI* Beck Depression Inventory, *BDI(−II)* Beck Depression Inventory(−II), *BIP-Q5* Brief Illness Perception Questionnaire 5, *BRCS* Brief Resilience Coping Scale, *CD-RISC* Connor-Davidson Resilience Scale, *CES-D* Center for Epidemiologic Studies Depression Scale, *CoVGAD-7* Generalized Anxiety Disorder Scale-7 for COVID-19 Anxiety, *CPDI* CoViD-19 Peritraumatic Distress Index, *CS* cross-sectional, *DASS-21* Depression Anxiety Stress Scale-21, *DT* Distress Thermometer, *EPDS* Edinburgh Postnatal Depression Scale, *EPDS-3A* Edinburgh Postnatal Depression Scale-Anxiety subscale, *FACIT-Sp12* Functional Assessment of Chronic Illness Therapy-Spiritual Well-Being Scale, *FCV-19S* Fear of COVID-19 scale, *GAD-2(−7)* Generalized Anxiety Disorder Scale-2(/−7), *GP* general population, *GPS-PTSD* Global Psychotrauma Scale-posttraumatic stress disorder subscale, *GSES* General Self-Efficacy Scale, *GSI* Global Severity Index, *HADS* Hospital Anxiety and Depression Scale, *HAI* Health Anxiety Inventory, *HAMA* Hamilton Anxiety Rating Scale, *HAMD* Hamilton Depression Rating Scale, *HCW* healthcare workers, *IES* Impact of Event Scale, *IES-R* Impact of Event Scale-Revised, *IQR* interquartile range, *ISI* Insomnia Severity Index, *IUS-12* Intolerance of Uncertainty Scale-Short Form, *JGLS* De Jong Gierveld Loneliness Scale, *K-6(/− 10)* Kessler Psychological Distress Scale-6(/− 10), *L* longitudinal, *MINI* Mini International Neuropsychiatric Interview, *MSPSS* Multidimensional Scale of Perceived Social Support, *NA* not available, *NRS* Numeric Rating Scale, *OBS* observational, *P* patients, *PANSS* Positive and Negative Syndrome Scale, *PCL-5(−C)* Post-traumatic Stress Disorder Checklist-5(/−Civilian Version), *PHQ-2(/−4/−9/− 15)* Patient Health Questionnaire-2(/−4/−9/− 15), *PROMIS-SFs* Patient Reported Outcomes Measurement Information System short forms, *PSQI* Pittsburgh Sleep Quality Index, *PSS(− 10)* Perceived Stress Scale(− 10), *PTSD-SS* Post-traumatic Stress Disorder Self-rating Scale, *PTSS* post-traumatic stress symptoms, *Ryff’s PWB* Ryff’s Psychological Wellbeing Scales, *SAS* Self-Rating Anxiety Scale, *SASR* Stanford Acute Stress Reaction, *SCL-90* Symptom Checklist-90, *SCS* Self-Compassion Scale, *SD* standard deviation, *SDQ* Strengths and Difficulties Questionnaire, *SDS* Self-Rating Depression Scale, *SF-12(/−36)* Short Form 12 Health Survey, *SHAI* Short Health Anxiety Inventory, *SOS* Stress Overload Scale, *SPEQ* Specific Psychotic Experience Questionnaire, *SRQ* Stress Response Questionnaire, *SRQ-20* 20-item Self-Report Questionnaire, *SSRS* Social Support Rating Scale, *SSS* Somatic Symptom Scale, *STAI-Y* State Trait Anxiety Inventory-Y, *SWLS* Satisfaction With Life Scale, *TAF* Triage Assessment Form, *TEMPS-A* Temperament Evaluation of Memphis, Pisa, Paris and San Diego-Anxious, *VAS* Visual Analogue Scale, *VDAS* Van Dream Anxiety Scale, *WHO-5* World Health Organization- Five Well-Being Index, *WHOQOL-BREF* abbreviated World Health Organization Quality of Life, *4−/5−/7−/11-P LS* 4−/5−/6−/11-point Likert-scale

^a^ developed by study authors

^b^ included in main analyses for general population but considered separately in subgroup-analyses

^c^ in Gao J et al. WHO-5 is used to assess depressive symptoms, in Badahdah et al. it is used to assess psychological distress

^d^ not directly reported

^e^ k-means-clustering method for the 4 tools summarized to ‘mental health’

Although we imposed no restrictions on the age limits, we identified no studies conducted in children but did find some studies in the general population that included participants below the age of 18 years [47, 58]^1^. Thus, the mean age of participants in the pandemic studies ranged from 20 (SD not reported) to 56.9 (SD 7.6) years [49, 99]^1^. The studies covered Asia (67 studies [26, 41–43, 49, 50, 54, 57, 58, 60, 62, 64, 71, 74, 76, 80, 81, 85, 86, 88–91, 93, 98, 101, 102, 104, 109, 111–116, 118–123, 126, 124, 128, 131–133, 136, 137, 140, 141, 143, 145]^1^ [18, 48, 59, 73, 84, 87, 92, 100, 105, 106, 108, 117, 134, 138, 139, 142, 144]^1,2^ [127]^1,^^3^) thereof from China [42, 49, 50, 57, 58, 60, 62, 64, 71, 80, 81, 85, 86, 88–91, 93, 101, 104, 109, 115, 116, 118–120, 122–124, 126, 128, 131–133, 136, 140, 141, 143, 145]^1 ^[18, 48, 59, 84, 87, 92, 100, 105, 106, 108, 117, 134, 138, 139, 142, 144]^1,2 ^[127]^1,3^, Europe (24 studies) [47, 99, 44–46, 51–53, 55, 61, 63, 65, 66, 68, 70, 110, 77, 78, 82, 83, 94, 97, 130, 103, 135]^1^, North America (six studies) [56, 67, 79, 107, 146, 147]^1^, or different continents (seven studies) [69, 72, 75, 95, 96, 125, 129]^1^. For 13 studies investigating more than one population, several samples were considered [130–133, 135, 140, 141, 143, 145]^1^ [134, 138, 139, 144]^1,2^. We identified 47 matching prepandemic comparative studies (eTable 10), including one pandemic study reporting adequate comparative data [127]^1,3^ [148–193]^3^.

Prevalence rates of the six mental health symptoms, that were considered for the review, were available for a varying number of included pandemic studies (Table 2). The proportion of participants beyond a cut-off value in the included studies varied considerably (eg, anxiety in general population: 0.7–64.0%). Based on cut-off values reported in the primary studies (eTable 11), we found increased levels of mental burden during the SARS-CoV-2 pandemic in the general population, healthcare workers, and patients regarding each of the symptoms observed during the current pandemic, that is, without considering the prepandemic situation.

**Table 2:** Narrative synthesis of prevalence based on scores above cut-off values for different mental health outcomes

	Number of studies^**a**^	Lowest reported prevalence (%)	Highest reported prevalence (%)
**General population**			
Anxiety, worries, fear	24 (18 GP, [45, 47, 49, 50, 52, 57, 63, 66, 68, 69, 71, 77, 93]^1^ [73, 84, 87]^1,2^ 6 M [132, 140, 145]^1^ [134, 139, 144]^1,2^)	0.67 (63)	64.0 (46)
Depressive symptoms	18 (13 GP [45, 49, 50, 52, 57, 63, 66, 68, 69, 71, 93]^1^ [84, 87]^1,2^, 5 M [132, 140, 145]^1^ [139, 144]^1,2^)	0.9 (89)	48.3 (48)
PTSS	7 (6 GP [52, 66, 71]^1^ [59, 84, 92]^1,2^, 1 M [132]^1^)	7.0 (51)	53.8 (55)
Sleep-related symptoms	6 (3 GP [71, 83]^1^ [84]^1,2^, 3 M [132]^1^ [134, 144]^1,2^)	0.9 (89)	37.6 (131)
Stress	5 (4 GP [66, 68, 71, 83]^1^, 1 M [132]^1^)	0.9 (89)	67.9 (55)
Psychological distress	7 (5 GP [41, 45, 58, 65, 81]^1^, 2 M [133, 136]^1^)	1.6 (90)	65.2 (112)
**Healthcare workers**			
Anxiety, worries, fear	22 (14 HCW [99, 95, 113, 102, 103, 110, 115, 120, 122, 121]^1^ [18, 100, 105, 106]^1,2^, 6 M [130, 140, 145]^1^ [134, 139, 144]^1,2^ )	7.0 (108)	92.0 (144)
Depressive symptoms	14 (9 HCW [97, 102, 110, 115, 120, 121, 122]^1^ [18, 106]^1,2^, 5 M [140, 145]^1^ [134, 139, 144]^1,2^)	0.6 (110)	50.4 (18)
PTSS	7 (HCW) [102, 110, 119, 120]^1^ [18, 105, 106]^1,2^	3.8 (82)	73.0 (83)
Sleep-related symptoms	9 (7 HCW [99, 102, 110, 115, 120]^1^ [18, 106]^1,2^, 2 M [134]^1^ [144]^1,2^)	8.27 (127)	38.0 (108)
Stress	6 (5 HCW [94, 102, 110, 111]^1 ^[108]^1,2^, 1 M [130]^1^)	5.2 (102)	56.5 (114)
Psychological distress	5 (4 HCW [96, 101, 112, 121]^1^, 1 M [145]^1^)	11.1 (101)	90.4 (145)
**Patients**			
Anxiety, worries, fear	6 (5P [123, 126, 129, 131, 143]^1^, 1 M [132]^1^)	19.5 (99)	80.2 (143)
Depressive symptoms	8 (7 P [123, 125, 126, 128, 131, 143]^1^ [127]^1,3^, 1 M [132]^1^)	27.8 (99)	55.3 (88)
PTSS	2 (1 P [123]^1^, 1 M [132]^1^)	31.0 (84)	43.4 (89)
Sleep-related symptoms	2 (1 P [128]^1^, 1 M [132]^1^)	27.6 (89)	66.3 (97)
Stress	1 (M [132]^1^)	17.0 (89)	
Psychological distress	1 (M [133]^1^)	13.1 (90)	

Abbreviations: *GP* general population, *HCW* healthcare workers, *M* mixed samples, *P* patients, *PTSS* posttraumatic stress symptoms

^a^ reporting prevalence rates for the respective mental health outcome

**Fig. 2:**
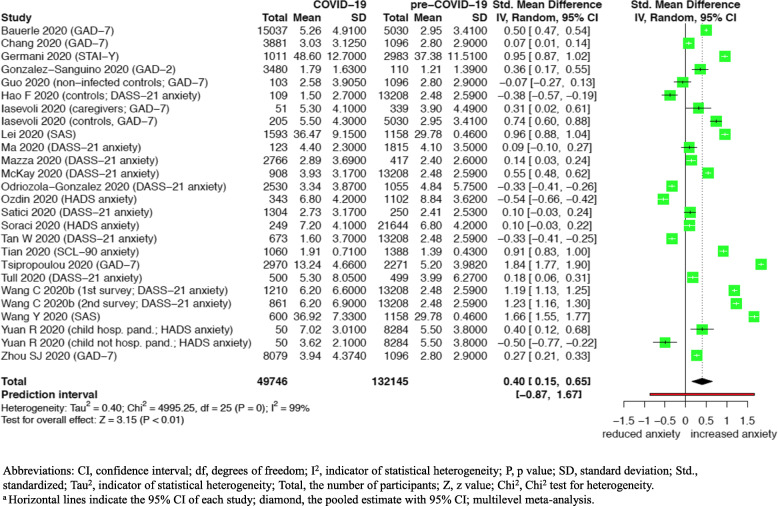
Forest plot main analysis, general population, anxiety

**Fig. 3:**
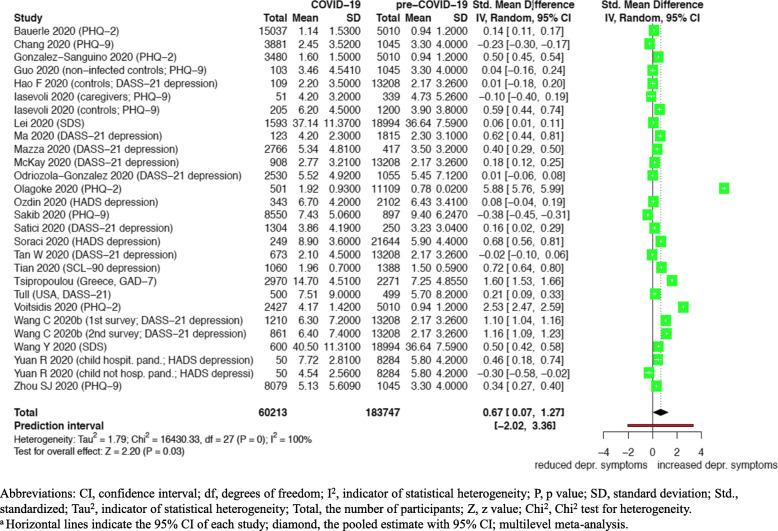
Forest plot main analysis, general population, depression

In pairwise meta-analyses comparing pandemic (early phase) with prepandemic data for the four primary outcomes, however, we found only evidence for a small increase of anxiety (standardized mean difference [SMD] 0.40; 95% CI 0.15–0.65; *p* = .002) and a moderate increase of depressive symptoms (SMD 0.67; 95% CI 0.07–1.27; *p* = .03) in the general population. No evidence for a change in stress or sleep-related symptoms was identified (Table 3). For healthcare workers compared with healthcare staff before the pandemic, the meta-analyses showed no evidence of any effect on the primary outcomes (Table 3). The same was found for patients (Table 3); however, prepandemic data in patients were only available for four samples. Forest plots are presented in Figs. 2, 3, and eResults 1 in the Additional file 2.

**Table 3:** Results of main and sensitivity analyses in three populations

Outcome	Studies (samples)	N (pandemic)	N (comp.)	Standardized mean difference (95% CI)	I^**2**^	95% prediction interval^**a**^
**Main analyses**						
**General population**						
Anxiety	23 (26)	49,746	132,145	0.40 (0.15–0.65)	99%	− 0.87–1.67
Depression	25 (28)	60,213	183,747	0.67 (0.07–1.27)	100%	−2.02–3.36
Stress	11 (13)	11,600	67,386	0.10 (−0.30–0.50)	100%	−1.39–1.60
Sleep-related symptoms	4 (4)	3332	7635	0.74 (−1.47–2.96)	100%	−3.68–5.17
**Healthcare workers**						
Anxiety	13 (14)	5508	22,204	−0.08 (−0.66–0.49)	99%	−1.75–1.58
Depression	7 (8)	2226	4605	−0.16 (− 0.59–0.26)	97%	−1.41–1.09
Stress	3 (3)	1570	2454	0.49 (−0.60–1.57)	99%	/
Sleep-related symptoms	4 (5)	554	20,024	0.83 (−0.14–1.81)	99%	−1.54–3.21
**Patients**						
Anxiety	6 (6)	1845	12,458	0.31 (−0.07, 0.69)	93%	−1.08–1.69
Depression	7 (7)	2138	24,444	0.48 (−0.08–1.04)	98%	−1.58–2.53
Stress	4 (4)	435	10,061	−0.10 (− 0.81–0.61)	98%	−3.54–3.34
Sleep-related symptoms	2 (2)	127	298	−0.61 (−1.75–0.54)	96%	/
**Sensitivity analysis – Quality of included pandemic studies (ie, exclusion of poor-quality studies)**						
**General population**						
Anxiety	16 (17)	38,323	81,350	0.53 (0.19–0.86)	100%	−0.90–1.95
Depression	18 (19)	48,790	136,884	0.83 (0.09–1.57)	100%	−2.17–3.82
Stress	7 (8)	9110	43,747	0.33 (−0.19–0.84)	100%	−1.20–1.85
Sleep-related symptoms	3 (3)	2659	6622	0.80 (−1.34–2.94)	100%	/
**Healthcare workers**						
Anxiety	4 (4)	1655	4124	−0.18 (−0.78–0.41)	97%	−1.30–0.94
Depression	4 (4)	1655	2356	0.03 (−0.42–0.47)	90%	−0.73–0.79
Stress	2 (2)	1376	1872	−0.05 (− 0.37–0.26)	95%	/
Sleep-related symptoms	1 (1)	123	4951	−0.03 (− 0.21–0.15)	/	/
**Patients**						
Anxiety	3 (3)	1461	11,116	0.45 (−0.10–1.01)	92%	/
Depression	3 (3)	1461	21,934	0.21 (−1.08–1.49)	99%	/
Stress	1 (1)	51	51	0.18 (−0.21–0.57)	/	/
Sleep-related symptoms	1 (1)	51	207	−0.03 (− 0.33–0.28)	/	/
**Sensitivity analysis – Level of comparability between included pandemic studies and comparative studies (ie, exclusion of level-3 and level-4 studies)**						
**General population**						
Anxiety	12 (13)	38,461	32,698	0.40 (0.06–0.74)	99%	−0.77–1.57
Depression	14 (15)	38,259	78,619	0.77 (−0.23–1.77)	100%	−2.72–4.25
Stress	7 (8)	8624	12,739	−0.15 (− 0.76–0.46)	99%	−1.84–1.53
Sleep-related symptoms	2 (2)	2550	5609	1.54 (−1.18–4.27)	100%	/
**Healthcare workers**						
Anxiety	7 (8)	3147	9511	−0.54 (−1.23–0.15)	99%	−2.11–1.03
Depression	4 (5)	546	2576	−0.38 (−1.56–0.79)	98%	−2.60–1.84
Stress	/	/	/	/	/	/
Sleep-related symptoms	3 (4)	423	19,804	1.01 (−0.17–2.18)	99%	−1.61–3.63
**Patients**						
Anxiety	4 (4)	1616	3184	0.23 (−0.33–0.79)	92%	−2.47–2.93
Depression	4 (4)	1704	3205	0 (−0.56–0.56)	93%	−2.69–2.70
Stress	2 (2)	127	217	0.15 (−0.08–0.37)	0%	/
Sleep-related symptoms	2 (2)	127	298	−0.61 (−1.75–0.54)	96%	/

Abbreviations: *CI* confidence interval, *comp.* comparative studies, *I*^*2*^ heterogeneity, *N* sample size, *pandemic* included pandemic studies

^a^ 95% prediction interval only calculated for meta-analyses with at least k = 4 studies

Of the 104 studies, 38 studies were judged to be of fair quality and 57 studies of poor quality, with main concerns regarding selection bias, the validity of outcome measures, and the description of the sample and the survey period (eTable 12). From nine high-quality studies, four were representative surveys [44, 47, 77, 88]^1^. From the 85 pairwise comparisons relevant for meta-analyses, 52 comparisons were of level-1 and 33 of level-2 quality (eTable 13). When excluding low-quality pandemic studies (Table 3), the effects on anxiety and depressive symptoms in the general population increased. The effect on anxiety in the general population was stable in the sensitivity analysis when only best comparable data sets (ie, level-1 and level-2 comparability) were included, while there was no longer evidence for an effect on depressive symptoms (Table 3 and eResults 2 in Additional file 2).

Heterogeneity was considerable in main and sensitivity analyses, with I^2^ scores mostly ranging from 90 to 100% and wide prediction intervals (Table 3). We therefore performed subgroup analyses with at least k = 5 studies in the main analyses in attempts to explain this heterogeneity (Table 4; eResults 3 in Additional file 2).

**Table 4:** Results of subgroup analyses for those populations and outcomes with at least k = 4 studies in main analysis

Subgroup analysis (subgroups)	Outcome	Test for subgroup differences^**a**^	Population	Subgroup difference: elevated effect^**b**^	Subgroup difference: reduced effect^**b**^
**Population characteristics (main studies)**					
Age • 30 years • > 30 ≤ 35 years • > 35 ≤ 40 years • > 40 ≤ 45 years • multiple age groups • age not specified	Anxiety	Chi^2^ = 9.5, df = 5 (*p* = .09)	GP	/	/
	Depression	Chi^2^ = 29.3, df = 5 (*p* < .001)	GP	≤30 years; > 40 ≤ 45 years	/
	Stress	Chi^2^ = 1043.3, df = 4 (*p* < .001)	GP	/	> 40 ≤ 45 years
	Anxiety	Chi^2^ = 8.7, df = 4 (*p* = .07)	HCW	/	/
	Depression	Chi^2^ = 2.2, df = 1 (*p* = .14)	HCW	/	/
	Sleep	Chi^2^ = 0.3, df = 1 (*p* = .57)	HCW	/	/
	Anxiety	Chi^2^ = 17.14, df = 4 (*p* = .002)	P	> 40 ≤ 45 years	
	Depression	Chi^2^ = 3.74, df = 4 (*p* = .44)	P	/	/
Stressor exposure • General population • Students • Others • Special exposure	Anxiety	Chi^2^ = 2.8, df = 3 (*p* = .42)	GP	/	/
	Depression	Chi^2^ = 1.9, df = 3 (*p* = .60)	GP	/	/
	Stress	Chi^2^ = 0.12, df = 3 (*p* = .99)	GP	/	/
Covid-19 patient contact • Low contact risk • High contact risk	Anxiety	Chi^2^ = 0, df = 1 (*p* = .95)	HCW	/	/
	Depression	Chi^2^ = 1.0, df = 1 (*p* = .31)	HCW	/	/
	Sleep	Chi^2^ = 0.2, df = 1 (*p* = .69)	HCW	/	/
Subgroup of patients • COVID-19 patients • Pregnant women • Psychiatric patients	Anxiety	Chi^2^ = 0.3, df = 2 (*p* = .88)	P	/	/
	Depression	Chi^2^ = 1.3, df = 2 (*p* = .51)	P	/	/
**Pandemic study characteristics**					
Survey start^c^ • ≤4 weeks • > 4 ≤ 6 weeks • > 6 ≤ 8 weeks • > 8 weeks • not specified	Anxiety	Chi^2^ = 3.55, df = 4 (*p* = .47)	GP	/	/
	Depression	Chi^2^ = 10.15, df = 4 (*p* = .04)	GP	> 8 weeks	/
	Stress	Chi^2^ = 0.31, df = 4 (*p* = .99)	GP	/	/
	Anxiety	Chi^2^ = 7.91, df = 4 (*p* = .10)	HCW	/	/
	Depression	Chi^2^ = 0.95, df = 2 (*p* = .62)	HCW	/	/
	Sleep	Chi^2^ = 4.21, df = 2 (*p* = .12)	HCW	/	/
	Anxiety	Chi^2^ = 4.58, df = 2 (*p* = .10)	P	/	/
	Depression	Chi^2^ = 3.08, df = 3 (*p* = .38)	P	/	/
Study conduction China • China • Non-China	Anxiety	Chi^2^ = 0.10, df = 1 (*p* = .75)	GP	/	/
	Depression	Chi^2^ = 0.60, df = 1 (*p* = .44)	GP	/	/
	Stress	Chi^2^ = 0.10, df = 1 (*p* = .76)	GP	/	/
	Anxiety	Chi^2^ = 2.84, df = 1 (*p* = .09)	HCW	/	/
	Depression	Chi^2^ = 0.08, df = 1 (*p* = .78)	HCW	/	/
	Sleep	Chi^2^ = 0.32, df = 1 (*p* = .57)	HCW	/	/
	Anxiety	Chi^2^ = 3.35, df = 1 (*p* = .07)	P	/	/
	Depression	Chi^2^ = 0.62, df = 1 (*p* = .43)	P	/	/
Outcome measure • AIS • BDI • DASS-21 • EDPS • EPDS-3A • GAD-2; GAD-7 • HADS • HAMA • HAMD • ISI • PHQ-2; PHQ-9 • PSQI • PSS • SAS • SDS • SCL-90 • STAI-Y	Anxiety	Chi^2^ = 10.7, df = 6 (*p* = .10)	GP	/	/
	Depression	Chi^2^ = 11.46, df = 5 (*p* = .04)	GP	PHQ-2	/
	Stress	Chi^2^ = 0.16, df = 1 (*p* = .69)	GP	/	/
	Anxiety	Chi^2^ = 2.80, df = 4 (*p* = .59)	HCW	/	/
	Depression	Chi^2^ = 2.91, df = 3 (*p* = .41)	HCW	/	/
	Sleep	Chi^2^ = 0.32, df = 1 (*p* = .57)	HCW	/	/
	Anxiety	Chi^2^ = 1.18, df = 4 (*p* = .88)	P	/	/
	Depression	Chi^2^ = 16.95, df = 5 (*p* = .005)	P	SDS; PHQ-9	/
Sample size • < 1000 • ≥1000	Anxiety	Chi^2^ = 1.86, df = 1 (*p* = .17)	GP	/	/
	Depression	Chi^2^ = 0.03, df = 1 (*p* = .86)	GP	/	/
	Stress	Chi^2^ = 2.31, df = 1 (*p* = .13)	GP	/	/
	Anxiety	Chi^2^ = 2.83, df = 1 (*p* = .09)	HCW	/	/
	Depression	Chi^2^ = 0, df = 1 (*p* = .96)	HCW	/	/
	Sleep	not possible	HCW	/	/
	Anxiety	Chi^2^ = 3.60, df = 1 (*p* = .06)	P	/	/
	Depression	Chi^2^ = 0.09, df = 1 (*p* = .77)	P	/	/
**Comparative study characteristics**					
Sample size • ≤500 • > 1000 ≤ 5000 • > 5000 ≤ 10,000 • > 10,000	Anxiety	Chi^2^ = 0.9, df = 3 (*p* = .83)	GP	/	/
	Depression	Chi^2^ = 3.5, df = 4 (*p* = .48)	GP	/	/
	Stress	Chi^2^ = 8.6, df = 3 (*p* = .03)	GP	/	> 5000 ≤ 10,000 participants
	Anxiety	Chi^2^ = 9.93, df = 3 (*p* = .02)	HCW	> 5000 ≤ 10,000 participants	
	Depression	Chi^2^ = 4.3, df = 2 (*p* = .12)	HCW	/	/
	Sleep	Chi^2^ = 0.3, df = 1 (*p* = .57)	HCW	/	/
	Anxiety	Chi^2^ = 0.1, df = 2 (*p* = .97)	P	/	/
	Depression	Chi^2^ = 3.9, df = 2 (*p* = .14)	P	/	/
Publication year • ≤1 year ago • ≤2 years ago • > 2 ≤ 5 years ago • > 5 ≤ 10 years ago • > 10 years ago	Anxiety	Chi^2^ = 8.0, df = 5 (*p* = .16)	GP	/	/
	Depression	Chi^2^ = 12.4, df = 5 (*p* = .03)	GP	> 10 years ago	/
	Stress	Chi^2^ = 11.6, df = 4 (*p* = .02)	GP	/	≤1 year ago
	Anxiety	Chi^2^ = 14.5, df = 3 (*p* = .002)	HCW	> 10 years ago	≤2 years ago
	Depression	Chi^2^ = 4.6, df = 1 (*p* = .03)	HCW	/	≤2 years ago
	Sleep	not possible	HCW	/	/
	Anxiety	Chi^2^ = 0.1, df = 2 (*p* = .94)	P	/	/
	Depression	Chi^2^ = 17.0, df = 5 (*p* = .005)	P	≤1 year ago; > 5 ≤ 10 years ago	/
**Pandemic and comparative study characteristics**					
Relationship samples sizes^d^ • Ratio ≥ 2 • Ratio ≥ 0.5 < 2 • Ratio ≥ 0.1 < 0.5 • Ratio < 0.1	Anxiety	Chi^2^ = 10.0, df = 3 (*p* = .02)	GP	Ratio ≥ 0.5 < 2	/
	Depression	Chi^2^ = 4.8, df = 3 (*p* = .19)	GP	/	/
	Stress	Chi^2^ = 0.4, df = 2 (*p* = .84)	GP	/	/
	Anxiety	Chi^2^ = 4.2, df = 2 (*p* = .12)	HCW	/	/
	Depression	Chi^2^ = 3.8, df = 2 (*p* = .15)	HCW	/	/
	Sleep	Chi^2^ = 0.32, df = 1 (*p* = .57)	HCW	/	/
	Anxiety	Chi^2^ = 17.7, df = 3 (*p* < .001)	P	Ratio ≥ 0.5 < 2; Ratio < 0.1	/
	Depression	Chi^2^ = 3.0, df = 3 (*p* = .39)	P	/	/

Abbreviations: *AIS* Athens Insomnia Scale, *BDI* Beck Depression Inventory, *DASS-21* Depression Anxiety Stress Scale-21, *df* degrees of freedom, *EPDS* Edinburgh Postnatal Depression Scale, *EPDS-3A* Edinburgh Postnatal Depression Scale-Anxiety subscale, *GAD* Generalized Anxiety Disorder Scale, *GP* general population, *HADS* Hospital Anxiety and Depression Scale, *HAMA* Hamilton Anxiety Rating Scale, *HAMD* Hamilton Depression Rating Scale, *HCW* healthcare workers, *p p* value, *P* patients, *PHQ* Patient Health Questionnaire, *PSS* Perceived Stress Scale, *SAS* Self-Rating Anxiety Scale, *SCL-90* Symptom Checklist-90, *SDS* Zung Self-Rating Depression Scale, *STAI-Y*, State Trait Anxiety Inventory-Y

^a^ Chi^2^ = test for subgroup differences

^b^ ordered by size of effect estimate (SMD)

^c^ since first COVID-19 cases in the respective country or, in case of China, since January 20, 2020

^d^ ratio of sample size in pandemic study vs comparative study

Regarding population characteristics (pandemic studies), age was no consistent risk or protective factor. Within the general population, we identified no evidence for a subgroup difference according to stressor exposure except for elevated sleep symptoms in isolated individuals [62]^1^. In healthcare workers, there was no evidence for a moderating effect of COVID-19 patient contact on mental health. In different groups of patients, we identified no evidence of differences in anxiety or depression. Compared with COVID-19 patients [131]^1^, psychiatric patients reported more stress, with the caveat of few studies [42, 132, 135]^1^.

Among general characteristics of the pandemic studies, we found no (consistent) evidence of differences depending on when the surveys started, whether they were conducted in China, or the sample size. We found evidence of an elevated level of depressive symptoms in the general population and patients depending on the specific outcome measure employed (eg, Patient Health Questionnaire [PHQ], Zung Self-Rating Depression Scale [SDS]).

In subgroup analyses for comparative study characteristics, there was no evidence of a consistent moderation of comparison sample sizes.

Across the three populations, we identified a higher level of anxiety and depressive symptoms if included studies were compared to prepandemic data published five or more years before versus a smaller burden in comparison to prepandemic data of less than 2 years ago.

The relationship of sample sizes explained the heterogeneity of the psychological impact of the SARS-CoV-2 pandemic in the general population and patients, with evidence for elevated symptoms of anxiety if similar sample sizes were compared.

The risk and protective factors narratively identified for each population are presented in Table 5 and eTables 14 and 15, with most of them being investigated in the general population, and few studies investigating protective factors at all. Most frequently named risk factors across the populations were pre-existing mental disorders, female sex, and concerns about COVID-19 infection, whereas most frequently reported protective factors were older age, good economic situation, and higher education.

**Table 5:** Risk and protective factors in three populations (mostly frequently reported factors)

	Risk factors^**a**^	Protective factors^**b**^
**General population**	- Mental disorder/or symptoms [44, 49, 51, 52, 58, 64, 69, 74, 78, 82, 83, 116, 132, 135]^1^) - Worries about relatives or oneself [51, 57, 64, 66, 74, 75, 80, 89, 82, 83]^1^ [48]^1,2^ - Being female [49, 52, 63, 66, 69, 72, 74, 79, 82, 83, 93]^1^ [vs 1x being male] - Previous (chronic) medical disease [52, 55, 63, 64, 69, 85, 135]^1^ - Being a student [52, 57, 60, 72, 146]^1^ - Personal/social worries about COVID-19 [51, 85, 86, 145]^1^ [48]^1,2^ - Physical symptoms [52, 66, 80, 85, 132]^1^ - Reduced perceived health [50, 57, 80, 85, 132]^1^ - No current relationship [57, 80, 81, 146]^1^ - Current local outbreak severity [57, 88, 93, 141]^1^ - History of stressful situations [52, 58, 63, 147]^1^ - Vulnerability to COVID-19 [53, 85, 146]^1^ - Health profession [66, 81, 141]^1^ - Own or close person’s quarantine [57, 62, 85]^1^	- Older age [49, 52, 63, 65, 66, 79, 91, 140, 147]^1^ - Good economic situation [52, 79, 88, 140, 146]^1^ [48]^1,2^ - Satisfaction with/level of information on COVID-19 [45, 49, 52, 85, 88, 93]^1^ - Not being single [ 66, 80, 88,86]^1^ - Higher education [50, 52, 66, 146]^1^ - Social support [52, 140]^1^ [48]^1,2^ - Being male [54, 65, 85]^1^
**Healthcare workers**	- Mental disorder/or symptoms [97, 115, 116, 119, 122]^1^ - Being female [98, 110, 119, 121]^1^ - Concern about infection with COVID-19 [103, 109, 120, 121]^1^ - Exposure to COVID-19 patients [94, 110, 115, 119]^1^ - Current local COVID-19 severity [94, 118, 141, 107]^1^	- Older age [98, 110]^1^
**Patients**	- (Suspected) COVID-19 [42, 131, 143]^1^ - Inflammatory markers in blood [42, 131]^1^ - Physical symptoms [132]^1^	- Higher education [127]^1,3^ - Good economic situation [127]^1,3^ - Higher lymphocyte ratio in blood [42]^1^ - Concomitant medical diseases [135]^1^

^a^ most frequently reported risk factors: general population: factor was reported as statistically significant risk factor in at least k = 3 studies; healthcare workers: factor reported in at least k = 4 studies; patients: factor reported in at least k = 2 studies

^b^ most frequently reported protective factors: general population: factor was reported as statistically significant protective factor in at least k = 3 studies; healthcare workers: factor reported in at least k = 2 studies (limited number of studies reporting protective factors in this group); patients: factor reported in k = 1 study (limited number of studies reporting protective factors in this group)

## Discussion

To our knowledge, this is the first systematic review and meta-analysis to assess the mental health impact of the SARS-CoV-2 pandemic in the general population, healthcare workers, and patients, by contrasting data from the early phase of the current pandemic with prepandemic data. We identified 104 independent studies, mainly in the general population, that suggest an increased prevalence of mental burden due to the SARS-CoV-2 pandemic. This finding is in line with previous reviews and meta-analyses that merely pooled the prevalence of or calculated the risk for mental burden in either one or several of these groups [20, 24, 29, 32, 33, 36].

On the other hand, the pairwise meta-analyses for 43 studies across the four primary outcomes revealed different results. Compared with prepandemic data, we only found an elevated level of some mental symptoms (anxiety, depression) due to the SARS-CoV-2 pandemic in the general population, but not of stress or sleeping problems.

Although healthcare workers were found to be a group at risk for mental health problems during the SARS-CoV-2 pandemic [18]^1,2^ [20, 29, 31, 32], we identified no evidence for an increased mental burden during the early phase when comparing them with healthcare staff prior to the pandemic. Because of a (chronic) work-related risk exposure in daily life [194], as a kind of ‘stress inoculation’, healthcare professionals might have learned effective strategies (eg, self-efficacy) helping them to cope more professionally with crises than other groups. In contrast to previous findings [20, 195], the level of COVID-19 patient contact did not affect the mental health impact.

Overall, the results of this review paint a more nuanced picture of the mental health consequences of the SARS-CoV-2 pandemic than previous reviews – an observation in line with stress resilience research that identified different trajectories of psychological adaptation after potentially traumatic events, ranging from no mental burden to severe mental illness [196, 197]. Indeed, a recent analysis of 523 healthy subjects from the German LORA study showed a decrease of perceived stress and stressor load while mental health improved during the eight-week measurement after lockdown, indicating that the pandemic and pandemic response may also have positive effects [198]. The number of studies reporting on protective factors in this review was rather limited, especially in healthcare workers and patients. However, these factors might also partly explain the heterogeneity of findings regarding mental health consequences. This is in line with positive aspects (eg, improved social relationships with close social contacts such as families) that were likewise reported for previous infectious disease outbreaks. The importance of taking a ‘resilience perspective’ in SARS-CoV-2 mental health research and investigating resilience factors has been pointed out previously [19, 22, 197, 199].

Several aspects must be considered when interpreting the results. First, the absence of evidence of effects in healthcare workers and patients in this review does not necessarily mean that there is evidence for the absence of effects of the SARS-CoV-2 pandemic on mental health in these groups. Second, for healthcare workers, the mental burden on individuals probably depends on the location of survey (eg, country, region) and how heavily the respective healthcare systems were burdened in the pandemic timeline (eg, number of hospitalized COVID-19 patients). Among the 13 included studies in meta-analyses for healthcare staff, we could only include a few studies from heavily burdened countries (eg, Italy: k = 2; Spain: k = 0; USA: k = 0). However, nine studies in these meta-analyses had been conducted in China, which, compared internationally, was less affected by the SARS-CoV-2 pandemic [4]. In the subgroup analysis regarding the level of COVID-19 patient contact, we assigned studies to the subgroup ‘high level of contact’ if at least 50% of the sample had close contact to COVID-19 patients (ie, ‘frontline healthcare workers’). However, the nature of contact was insufficiently described in the included studies.

Strengths of this review compared with previous publications include the systematic search for comparative prepandemic data for inclusion in pairwise meta-analyses, the stepwise selection of prepandemic studies to ensure best available comparability, and the population-specific analysis of risk and protective factors. One limitation refers to the search methods for pandemic studies (eg, no preprints; no reference lists of reviews) and comparative data (eg, subgroups in general population only partially searched). We had no restrictions regarding the publication format except for the exclusion of preprints which might be viewed as limitation. This restriction might have affected the evidence found in this review compared to others (eg, Cochrane reviews) where preprint articles are included.

The large between-study heterogeneity, a problem shared by previous meta-analyses [20, 24, 32, 33], could not be fully explained by subgroup analyses. This heterogeneity probably resulted from differences between the pandemic studies (eg, countries, sociocultural differences in the perception of mental burden, pandemic outbreak severity, subpopulations, outcome measures) and variability between the comparative studies (eg, study design, outcome measures), respectively. Among the pandemic studies, especially the specific outcome measures used were an important source of heterogeneity. Furthermore, the pandemic and comparative data were heterogeneous (eg, country, population), which could be partially captured by our self-developed tool for the level of comparability and was controlled for by the corresponding sensitivity analysis. We cannot preclude that moderators of effects are present that we, though our best efforts, did not identify and therefore could not control for. Besides, comparative studies with larger sample sizes were preferred, leading to small 95% CIs and a lack of CI overlap with pandemic study findings. Despite the comprehensiveness of this review compared to previous publications, the small number of studies in certain subgroups potentially limited the statistical power (eg, surveys including students).

Apart from specific outcome measures, less recent comparative data, and homogenous sample sizes, the subgroup analyses indicated no consistent determinants of heterogeneity. An elevated level of depression based on the assessment with the PHQ and SDS might – at least for the PHQ-9 – be explained by the high sensitivity to change of this instrument and its usefulness to monitor treatment outcomes [200, 201]. Given the increased mental burden if pandemic studies were compared to older prepandemic data, cohort effects cannot be excluded.

Discrepancies between subgroup analyses and the narrative synthesis of risk and protective factors (eg, COVID-19 patient contact) might be due to methodological differences. Because of the primary use of screening but not diagnostic tools to determine mental burden in the included pandemic studies, this review does not allow any conclusions concerning a putative increase of diagnoses of mental disorders during the early phase of the SARS-CoV-2 pandemic. Consistent with the synthesis of risk factors, the meta-analyses partly showed an increased level of mental symptoms in young and middle-aged groups, in line with previous studies [12]. However, more studies including elderly would be needed to clearly investigate age differences, and whether the pandemic works as a ‘burning lens’ for the already increased mental burden in young people [202]. Finally, given the pandemic timeline, the evidence is substantially based on Chinese studies thus potentially limiting the transferability of findings to other contexts.

Further research in other countries (eg, USA), that started later on during the pandemic, could change the findings. The latter is also supported by the wide prediction intervals identified in this review, which indicate uncertainty in our conclusions about whether the pandemic and related stressors do affect mental health [203].

The review has several implications for research and practice. There is an urgent need for representative surveys, in order to allow fair comparisons between the mental burden caused by SARS-CoV-2 in different countries and to examine other risk and protective factors (eg, cultural context). Representative surveys in the general population might also serve to identify specific subgroups at risk for which further studies would be needed. From a public mental health perspective, a stronger focus on (psychosocial) protective factors for mental health would be desirable to derive appropriate contents for preventive measures (eg, pandemic preparedness plans) or health-promoting interventions (eg, resilience training) prior to, during, and after a pandemic [199]. By further investigating the mental health impact of specific stressors – in line with Brooks and colleagues [13] – researchers and practitioners might gain further knowledge about when (eg, in pandemic timeline) and for whom (eg, after exposure to which stressors) interventions should be implemented to buffer negative mental health effects of SARS-CoV-2.

## Conclusions

In conclusion, compared with prepandemic data, this review shows different adverse mental health consequences of the early phase of the SARS-CoV-2 pandemic in the examined population groups in contrast to previous research, with healthcare workers being more resilient than expected. The quality of studies varies. High-quality, representative surveys in the general population and specific subpopulations, longitudinal studies, and further research efforts on protective factors are needed to better understand the psychological impacts of the SARS-CoV-2 pandemic and to help design effective preventive measures and interventions that are tailored to the needs of specific population groups.

## Supplementary Information

**Additional file 1: **Methods of the systematic review with meta-analyses. **eTable 1.** MOOSE Checklist. **eTable 2.** Differences between protocol and review. **eMethods 1.** Search strategies for SARS-CoV-2 (‘pandemic’) studies. **eMethods 2.** Search strategy for prepandemic comparative studies. **eTable 3.** Eligibility criteria for SARS-CoV-2 pandemic studies. **eTable 4.** Eligibility criteria for prepandemic comparative studies. **eTable 5.** Eligibility criteria for pairwise meta-analyses. **eTable 6.** Customized data extraction sheet. **eTable 7.** Modified quality assessment tool. **eTable 8.** Rating of comparability between pandemic and prepandemic comparative studies. **eMethods 3.** Further methodological details of this systematic review and meta-analyses.

**Additional file 2: **Results of the systematic review with meta-analyses. **eTable 9.** Details on number of included (pandemic and comparative) studies. **eTable 10.** Study characteristics of the prepandemic comparative studies. **eTable 11.** Cut-off values reported in included pandemic studies. **eResults 1.** Forest plots of main analyses. **eTable 12.** Quality assessment of included pandemic studies. **eTable 13.** Assessment of level of comparability between pandemic and prepandemic comparative studies. **eResults 2.** Forest plots of sensitivity analyses. **eResults 3.** Detailed results of subgroup analyses. **eTable 14.** Risk factors in the general population, healthcare workers, and patients. **eTable 15.** Protective factors in the general population, healthcare workers, and patients.

## Data Availability

All data generated or analyzed during this study are included in this published article and its supplementary information files. Additional data (eg, detailed extracted data) are available from the corresponding author on request.

## Abbreviations

COVID-19: Coronavirus disease 2019
LORA: Longitudinal Resilience Assessment
MOOSE: Meta-analyses Of Observational Studies in Epidemiology
NIH: National Institutes of Health
PHQ: Patient Health Questionnaire
PRISMA: Preferred Reporting Items for Systematic Reviews and Meta-Analysis
PROSPERO: International Prospective Register of Systematic Reviews
SARS-CoV(− 2): Severe Acute Respiratory Syndrome Coronavirus (− 2)
SD: Standard deviation
SDS: Zung Self-Rating Depression Scale
SMD: Standardized mean difference

## Footnotes

1 included pandemic studies.

2 studies already considered in previous systematic review (Gilan, Röthke and colleagues) [19].

3 prepandemic comparative studies.
