# Supplementary material for: Mental burden and its risk and protective factors during the early phase of the SARS-CoV-2 pandemic: systematic review and meta-analyses
Source: Global Health. 2021 Mar 29;17:34. doi: 10.1186/s12992-021-00670-y (PMC8006628; doi:10.1186/s12992-021-00670-y)
Supplement: Supplementary file 1 — Additional file 1: Methods of the systematic review with meta-analyses. eTable 1. MOOSE Checklist. eTable 2. Differences between protocol and review. eMethods 1. Search strategies for SARS-CoV-2 (‘pandemic’) studies. eMethods 2. Search strategy for prepandemic comparative studies. eTable 3. Eligibility criteria for SARS-CoV-2 pandemic studies. eTable 4. Eligibility criteria for prepandemic comparative studies. eTable 5. Eligibility criteria for pairwise meta-analyses. eTable 6. Customized data extraction sheet. eTable 7. Modified quality assessment tool. eTable 8. Rating of comparability between pandemic and prepandemic comparative studies. eMethods 3. Further methodological details of this systematic review and meta-analyses. [file 12992_2021_670_MOESM1_ESM.docx]

**Additional file 1**

Mental burden and its risk and protective factors during the early phase of the SARS-CoV-2 pandemic: systematic review and meta-analyses

Angela M Kunzler*, Nikolaus Röthke*, Lukas Günthner, Jutta Stoffers-Winterling, Oliver Tüscher, Michaela Coenen, Eva Rehfuess, Guido Schwarzer, Harald Binder, Christine Schmucker, Jörg Meerpohl, Klaus Lieb

**eTable 1.** MOOSE Checklist

**eTable 2.** Differences between protocol and review

**eMethods 1.** Search strategies for SARS-CoV-2 (‘pandemic’) studies

**eMethods 2.** Search strategy for prepandemic comparative studies

**eTable 3.** Eligibility criteria for SARS-CoV-2 pandemic studies

**eTable 4.** Eligibility criteria for prepandemic comparative studies

**eTable 5.** Eligibility criteria for pairwise meta-analyses

**eTable 6.** Customized data extraction sheet

**eTable 7.** Modified quality assessment tool

**eTable 8.** Rating of comparability between pandemic and prepandemic comparative studies

**eMethods 3.** Further methodological details of this systematic review and meta-analyses

**eTable 1. MOOSE Checklist for Meta-analyses of Observational Studies in Epidemiology^1^**

| **Item No** | **Recommendation** | **Reported on Page No** |
| --- | --- | --- |
| Reporting of background should include | | |
| 1 | Problem definition | 2-3 |
| 2 | Hypothesis statement | 3 |
| 3 | Description of study outcome(s) | 3 |
| 4 | Type of exposure or intervention used | 3, S1.13 (eTable 3) |
| 5 | Type of study designs used | 3, S1.13 (eTable 3) |
| 6 | Study population | 3, S1.13 (eTable 3) |
| Reporting of search strategy should include | | |
| 7 | Qualifications of searchers (eg, librarians and investigators) | 25 |
| 8 | Search strategy, including time period included in the synthesis and key words | S1.6-12 (eMethods 1+2) |
| 9 | Effort to include all available studies, including contact with authors | S1.16-17 (eTable 6), S1.23-25 (eMethods 3) |
| 10 | Databases and registries searched | 3, S1.6-9 (eMethods 1) |
| 11 | Search software used, name and version, including special features used (eg, explosion) | 3, S1.6-9 (eMethods 1) |
| 12 | Use of hand searching (eg, reference lists of obtained articles) | S1.6-9 (eMethods 1) |
| 13 | List of citations located and those excluded, including justification | 4 (Figure 1) |
| 14 | Method of addressing articles published in languages other than English | S1.13 (eTable 3) |
| 15 | Method of handling abstracts and unpublished studies | S1.6-9 (eMethods 1) |
| 16 | Description of any contact with authors | S1.16-17 (eTable 6), S1.23-26 (eMethods 3) |
| Reporting of methods should include | | |
| 17 | Description of relevance or appropriateness of studies assembled for assessing the hypothesis to be tested | 3, S1.16-17 (eTable 6), S1.23-26 (eMethods 3) |
| 18 | Rationale for the selection and coding of data (eg, sound clinical principles or convenience) | 3, S1.16-17 (eTable 6), S1.23-26 (eMethods 3) |
| 19 | Documentation of how data were classified and coded (eg, multiple raters, blinding and interrater reliability) | 3, S1.6-9 (eMethods 1) |
| 20 | Assessment of confounding (eg, comparability of cases and controls in studies where appropriate) | S2.27-29 (eTable 13) |
| 21 | Assessment of study quality, including blinding of quality assessors, stratification or regression on possible predictors of study results | S2.19-26 (eTable 12) |
| 22 | Assessment of heterogeneity | 4-5, S1.23-26 (eMethods 3) |
| 23 | Description of statistical methods (eg, complete description of fixed or random effects models, justification of whether the chosen models account for predictors of study results, dose-response models, or cumulative meta-analysis) in sufficient detail to be replicated | S1.23-26 (eMethods 3) |
| 24 | Provision of appropriate tables and graphics | 4 (Figure 1), 6-23 (Table 1-5, Figure 23, S1.2-5 (eTable 1-2), S1.13-22 (eTable 3-8), S1.25 (Table eMethods 3), S2.1-59 (eTable 9-15, eFigure 1-35) |
| Reporting of results should include | | |
| 25 | Graphic summarizing individual study estimates and overall estimate | 17-20 (Table 2-3, Figure 2-3), S2.14-18 (eFigure 1-12) |
| 26 | Table giving descriptive information for each study included | 6-16 (Table 1) |
| 27 | Results of sensitivity testing (eg, subgroup analysis) | 18-19 (Table 3), 21-23 (Table 4), S2.30-50 (Tables eResults 3, eFigure 13-35) |
| 28 | Indication of statistical uncertainty of findings | 5, 18-19 (Table 3) |

**eTable 2. Differences between protocol (PROSPERO registration no. CRD42020193249) and final review**

|  | **Protocol registration** | **Reason for changes and changes made in final review** |
| --- | --- | --- |
| Meta-analyses | Pairwise meta-analyses using odds ratios as effect estimate based on studies reporting the prevalence of mental symptoms as dichotomous variable | In a pilot search for prepandemic comparative prevalence rates, the lack of prevalence data for most of the outcome measures became evident. Instead, we tried to contact the authors of included pandemic studies reporting prevalence rates to request the respective descriptive statistics (ie, means and standard deviations), in order to calculate standardized mean differences. |
| Meta-analyses | Inclusion of outcome posttraumatic stress (symptoms) in pairwise meta-analyses | We did not find any prepandemic comparative data for posttraumatic stress symptoms meeting our criteria (see eTable 4). |
| Meta-analyses | Meta-analyses for anxiety | Meta-analyses were only performed for (state) scales of anxiety. Therefore, the following outcome measures were not considered for the pairwise meta-analyses in the final publication:   - COVID-19-related fear scales (FCV-19S): not possible to find prepandemic comparative data; not state anxiety - Somatic Symptom Scale (SSS) with subscales for depressive and anxiety scales: not possible to find prepandemic comparative data - “Anxiety scale”: no further details regarding the scale by Xu J et al^81^ - BIP-Q5: Brief Illness Perception Questionnaire 5: not state anxiety - Symptom Checklist-90 (SCL-90) phobic anxiety: not state anxiety - VDAS: Van Dream Anxiety Scale: not state anxiety - Temperament Evaluation of Memphis, Pisa, Paris and San Diego-auto questionnaire version (TEMPS-A): not state anxiety - Visual Analogue Scale (VAS) for anxiety (and stress) in Gan et al^70^: not possible to find comparative data (means) - VAS for anxiety: Shevlin et al^128^ also used the General Anxiety Disorder Scale-7 (GAD-7); however, in case of studies using multiple scales for one outcome, we considered the outcome measure which was most frequently reported among all eligible (ie, validated) scales - Numeric Rating Scale (NRS) for fear: fear and not anxiety |
| Subgroup analyses | Planned subgroup analyses for issues regarding the methodological quality (eg, assessments methods, statistical methods used) | The subgroup analyses in the final review differed from the planned analyses as new potential factors became evident during the review development process that might explain the between-study heterogeneity (eg, age, country, characteristics of comparative data). |
| Quality assessment | Quality assessment tool | The quality assessment tool that was stated in the registration was adapted for this review. |

**eMethods 1. Search strategies for SARS-CoV-2 (“pandemic”) studies**

The search strategy for the three bibliographic databases PubMed, PsycINFO, and Web of Science (Core Collection) comprised three key sets of terms: 1) terms associated with mental health, psychological distress, and resilience (eg, “mental health”, “burden”, “resilien*”), 2) pandemic-related terms (eg, “COVID-19”, “coronavirus”), and 3) the populations of interest including the general population, healthcare workers, and patients (eg, “community”, “health* personnel”, “patients”). As appropriate for each database, various search terms for each of the clusters were used (eg, MeSH terms, keywords, title/abstract), combined by Boolean operators and using truncation.

In order to allow further systematic reviews at later time points, the search included other epidemic- and pandemic-related terms (eg, “ebola”), that were not relevant for the current review. For the same reason, the original search, performed on April 16, 2020, was limited to the time period January 1, 1990 to April 16, 2020 (manually for PubMed and PsycINFO and directly in Web of Science). A search update was performed on May 29, 2020. Subsequently, due to the focus of this review on the *SARS-CoV-2 pandemic*, the search results from the three databases were then manually restricted to the period from January 1, 2019 to May 29, 2020 for the current review. We decided to limit the search results to this period given that first infections with SARS-CoV-2 occurred in December 2019 and studies concerning the associated mental health impact and potential risk or protective factors had only been conducted after this date.

The reference lists of included studies were inspected to identify any additional eligible studies. We did not consider unpublished or preprint articles for this review and did not inspect the reference lists of previous systematic reviews or meta-analyses.

PubMed

Searched 29 May 2020 [1920 records]

#1 "Mental health"[mh]

#2 "mental health"[tw]

#3 psychological[tw]

#4 Resilience, psychological[mh]

#5 resilien*[tiab] or hardiness*[tiab]

#6 "post-traumatic growth"[tiab] or "posttraumatic growth"[tiab] or "stress-related growth"[tiab]

#7 "resilience factor*"[tiab] or "protective factor*"[tiab] or resource*[tiab]

#8 burden[tiab]

#9 #1 or #2 or #3 or #4 or #5 or #6 or #7 or #8

#10 SARS[tiab] OR influenza[tiab] or flu[tiab] or MERS[tiab] or ebola[tiab]

#11 Pandemic[mh] or pandem*[tw]

#12 coronavirus[tw] or COVID-19[tw] or 2019-nCoV[tw] or SARS-CoV-2[tw]

#13 Quarantine[mh] or quarantine[tw]

#14 #10 or #11 or #12 or #13

#15 #9 and #14

#16 "health personnel"[mh]

#17 "health* personnel"[tiab] or "health* profession*"[tiab] or "health* worker*"[tiab] or "health* practitioner*"[tiab] or "health* provider*"[tiab] or "health* staff"[tiab]

#18 "care personnel"[tiab] or “care profession*"[tiab] or “care worker*"[tiab] or “care practitioner*"[tiab] or “care provider*"[tiab] or “care staff"[tiab]

#19 "intensive care"[tiab] or ICU[tiab]

#20 emergency[tiab]

#21 ambulance[tiab] or paramedic*[tiab]

#22 "hospital personnel" [tiab] or "hospital staff" [tiab]

#23 physician*[tiab] or doctor*[tiab]

#24 nurse*[tiab] or "nursing staff"[tiab]

#25 #16 or #17 or #18 or #19 or #20 or #21 or #22 or #23 or #24

#26 Patients[mh] or patients[tiab]

#27 older[tiab] or geriatric[tiab]

#28 #26 or #27

#29 "population health"[mh]

#30 public[tiab]

#31 society[tiab] or social[tiab]

#32 community[tiab]

#33 "general population"[tiab]

#34 nationwide[tiab]

#35 #29 or #30 or #31 or #32 or #33 or #34

#36 #25 or #28 or #35

#37 #15 and #36

PsycINFO Ovid

Searched 29 May 2020 [233 records]

S1 MA Mental health

S2 TX Mental Health

S3 TX psychological

S4 MA Resilience, psychological

S5 TI resilien* OR AB resilien* OR TI hardiness* OR AB hardiness*

S6 TI posttraumatic growth OR AB posttraumatic growth OR TI post-traumatic growth OR AB post-traumatic growth OR TI stress-related growth OR AB stress-related growth

S7 TI resilience factor* OR AB resilience factor* OR TI protective factor* OR AB protective factor* OR TI resource* OR AB resource*

S8 TI burden OR AB burden

S9 S1 OR S2 OR S3 OR S4 OR S5 OR S6 OR S7 OR S8

S10 TI SARS OR AB SARS OR TI Influenza OR AB Influenza OR TI Flu OR AB Flu OR TI MERS OR AB MERS OR TI Ebola OR AB Ebola

S11 MA pandemic OR TX pandem*

S12 TX coronavirus OR TX COVID-19 OR TX 2019-nCoV OR TX SARS-CoV-2

S13 MA quarantine OR TX quarantine

S14 S10 OR S11 OR S12 OR S13

S15 S9 AND S14

S16 MA health personnel

S17 TI health* personnel OR AB health* personnel OR TI health* profession* OR AB health* profession* OR TI health* worker* OR AB health* worker* OR TI health* practitioner OR AB health* practitioner OR TI health* provider* OR AB health* provider* OR TI health* staff OR AB health* staff

S18 TI care personnel OR AB care personnel OR TI care profession* OR AB care profession* OR TI care worker* OR AB care worker* OR TI care practitioner* OR AB care practitioner* OR TI care provider* OR AB care provider* OR TI care staff OR AB care staff

S19 TI intensive care OR AB intensive care OR TI ICU OR AB ICU

S20 TI emergency OR AB emergency

S21 TI ambulance OR AB ambulance OR TI paramedic* OR AB paramedic*

S22 TI hospital personnel OR AB hospital personnel OR TI hospital staff OR AB hospital staff

S23 TI physician* OR AB physician* OR TI doctor* OR AB doctor*

S24 TI nurse* OR AB nurse* OR TI nursing staff OR AB nursing staff

S25 S16 OR S17 OR S18 OR S19 OR S20 OR S21 OR S22 OR S23 OR S24

S26 MA patients OR TI patients OR AB patients

S27 TI older OR AB older OR TI geriatric OR AB geriatric

S28 S26 OR S27

S29 MA population health

S30 TI public OR AB public

S31 TI society OR AB society OR TI social OR AB social

S32 TI community OR AB community

S33 TI general population OR AB general population

S34 TI nationwide OR AB nationwide

S35 S29 OR S30 OR S31 OR S32 OR S33 OR S34

S36 S25 OR S28 OR S35

S37 S15 AND S36

Web of Science Core Collection

Searched 2019 to 29 May 2020 [276 records]

# 36 #33 AND #13

Refined by: WEB OF SCIENCE CATEGORIES: (PUBLIC ENVIRONMENTAL OCCUPATIONAL HEALTH OR PSYCHOLOGY OR PSYCHOLOGY CLINICAL OR PRIMARY HEALTH CARE OR PSYCHIATRY OR NURSING OR PSYCHOLOGY MULTIDISCIPLINARY)

Indexes=SCI-EXPANDED, SSCI, A&HCI, CPCI-S, CPCI-SSH, BKCI-S, BKCI-SSH, ESCI, CCR-EXPANDED, IC Timespan=1990-2020

# 35 #33 AND #13

Refined by: WEB OF SCIENCE CATEGORIES: (INFECTIOUS DISEASES OR PUBLIC ENVIRONMENTAL OCCUPATIONAL HEALTH OR IMMUNOLOGY OR PSYCHOLOGY OR VIROLOGY OR PSYCHOLOGY CLINICAL OR PRIMARY HEALTH CARE OR PSYCHIATRY OR NURSING OR SOCIAL SCIENCES INTERDISCIPLINARY OR PSYCHOLOGY MULTIDISCIPLINARY OR SOCIAL WORK)

Indexes=SCI-EXPANDED, SSCI, A&HCI, CPCI-S, CPCI-SSH, BKCI-S, BKCI-SSH, ESCI, CCR-EXPANDED, IC Timespan=1990-2020

# 34 #33 AND #13

Indexes=SCI-EXPANDED, SSCI, A&HCI, CPCI-S, CPCI-SSH, BKCI-S, BKCI-SSH, ESCI, CCR-EXPANDED, IC Timespan=1990-2020

# 33 #32 OR #25 OR #22

Indexes=SCI-EXPANDED, SSCI, A&HCI, CPCI-S, CPCI-SSH, BKCI-S, BKCI-SSH, ESCI, CCR-EXPANDED, IC Timespan=1990-2020

# 32 #31 OR #30 OR #29 OR #28 OR #27 OR #26

Indexes=SCI-EXPANDED, SSCI, A&HCI, CPCI-S, CPCI-SSH, BKCI-S, BKCI-SSH, ESCI, CCR-EXPANDED, IC Timespan=1990-2020

# 31 TS=nationwide

Indexes=SCI-EXPANDED, SSCI, A&HCI, CPCI-S, CPCI-SSH, BKCI-S, BKCI-SSH, ESCI, CCR-EXPANDED, IC Timespan=1990-2020

# 30 TS="general population"

Indexes=SCI-EXPANDED, SSCI, A&HCI, CPCI-S, CPCI-SSH, BKCI-S, BKCI-SSH, ESCI, CCR-EXPANDED, IC Timespan=1990-2020

# 29 TS=community

Indexes=SCI-EXPANDED, SSCI, A&HCI, CPCI-S, CPCI-SSH, BKCI-S, BKCI-SSH, ESCI, CCR-EXPANDED, IC Timespan=1990-2020

# 28 TS=(society or social)

Indexes=SCI-EXPANDED, SSCI, A&HCI, CPCI-S, CPCI-SSH, BKCI-S, BKCI-SSH, ESCI, CCR-EXPANDED, IC Timespan=1990-2020

# 27 TS=public

Indexes=SCI-EXPANDED, SSCI, A&HCI, CPCI-S, CPCI-SSH, BKCI-S, BKCI-SSH, ESCI, CCR-EXPANDED, IC Timespan=1990-2020

# 26 TS="population health"

Indexes=SCI-EXPANDED, SSCI, A&HCI, CPCI-S, CPCI-SSH, BKCI-S, BKCI-SSH, ESCI, CCR-EXPANDED, IC Timespan=1990-2020

# 25 #24 OR #23

Indexes=SCI-EXPANDED, SSCI, A&HCI, CPCI-S, CPCI-SSH, BKCI-S, BKCI-SSH, ESCI, CCR-EXPANDED, IC Timespan=1990-2020

# 24 TS=(older or geriatric)

Indexes=SCI-EXPANDED, SSCI, A&HCI, CPCI-S, CPCI-SSH, BKCI-S, BKCI-SSH, ESCI, CCR-EXPANDED, IC Timespan=1990-2020

# 23 TS=patients

Indexes=SCI-EXPANDED, SSCI, A&HCI, CPCI-S, CPCI-SSH, BKCI-S, BKCI-SSH, ESCI, CCR-EXPANDED, IC Timespan=1990-2020

# 22 #21 OR #20 OR #19 OR #18 OR #17 OR #16 OR #15 OR #14

Indexes=SCI-EXPANDED, SSCI, A&HCI, CPCI-S, CPCI-SSH, BKCI-S, BKCI-SSH, ESCI, CCR-EXPANDED, IC Timespan=1990-2020

# 21 TS=(nurse* or "nursing staff")

Indexes=SCI-EXPANDED, SSCI, A&HCI, CPCI-S, CPCI-SSH, BKCI-S, BKCI-SSH, ESCI, CCR-EXPANDED, IC Timespan=1990-2020

# 20 TS=(physician* or doctor*)

Indexes=SCI-EXPANDED, SSCI, A&HCI, CPCI-S, CPCI-SSH, BKCI-S, BKCI-SSH, ESCI, CCR-EXPANDED, IC Timespan=1990-2020

# 19 TS=("hospital personnel" or "hospital staff")

Indexes=SCI-EXPANDED, SSCI, A&HCI, CPCI-S, CPCI-SSH, BKCI-S, BKCI-SSH, ESCI, CCR-EXPANDED, IC Timespan=1990-2020

# 18 TS=(ambulance or paramedic*)

Indexes=SCI-EXPANDED, SSCI, A&HCI, CPCI-S, CPCI-SSH, BKCI-S, BKCI-SSH, ESCI, CCR-EXPANDED, IC Timespan=1990-2020

# 17 TS=emergency

Indexes=SCI-EXPANDED, SSCI, A&HCI, CPCI-S, CPCI-SSH, BKCI-S, BKCI-SSH, ESCI, CCR-EXPANDED, IC Timespan=1990-2020

# 16 TS=("intensive care" or ICU)

Indexes=SCI-EXPANDED, SSCI, A&HCI, CPCI-S, CPCI-SSH, BKCI-S, BKCI-SSH, ESCI, CCR-EXPANDED, IC Timespan=1990-2020

# 15 TS=("care personnel" or "care profession*" or "care worker*" or "care practitioner*" or "care provider*" or "care staff")

Indexes=SCI-EXPANDED, SSCI, A&HCI, CPCI-S, CPCI-SSH, BKCI-S, BKCI-SSH, ESCI, CCR-EXPANDED, IC Timespan=1990-2020

# 14 TS=("health* personnel" or "health* profession*" or "health* worker*" or "health* practitioner*" or "health* provider*" or "health* staff")

Indexes=SCI-EXPANDED, SSCI, A&HCI, CPCI-S, CPCI-SSH, BKCI-S, BKCI-SSH, ESCI, CCR-EXPANDED, IC Timespan=1990-2020

# 13 #12 AND #7

Indexes=SCI-EXPANDED, SSCI, A&HCI, CPCI-S, CPCI-SSH, BKCI-S, BKCI-SSH, ESCI, CCR-EXPANDED, IC Timespan=1990-2020

# 12 #11 OR #10 OR #9 OR #8

Indexes=SCI-EXPANDED, SSCI, A&HCI, CPCI-S, CPCI-SSH, BKCI-S, BKCI-SSH, ESCI, CCR-EXPANDED, IC Timespan=1990-2020

# 11 TS=quarantine

Indexes=SCI-EXPANDED, SSCI, A&HCI, CPCI-S, CPCI-SSH, BKCI-S, BKCI-SSH, ESCI, CCR-EXPANDED, IC Timespan=1990-2020

# 10 TS=(coronavirus or COVID-19 or 2019-nCoV or SARS-CoV-2)

Indexes=SCI-EXPANDED, SSCI, A&HCI, CPCI-S, CPCI-SSH, BKCI-S, BKCI-SSH, ESCI, CCR-EXPANDED, IC Timespan=1990-2020

# 9 TS=pandem*

Indexes=SCI-EXPANDED, SSCI, A&HCI, CPCI-S, CPCI-SSH, BKCI-S, BKCI-SSH, ESCI, CCR-EXPANDED, IC Timespan=1990-2020

# 8 TS=(SARS or influenza or flu or MERS or ebola)

Indexes=SCI-EXPANDED, SSCI, A&HCI, CPCI-S, CPCI-SSH, BKCI-S, BKCI-SSH, ESCI, CCR-EXPANDED, IC Timespan=1990-2020

# 7 #6 OR #5 OR #4 OR #3 OR #2 OR #1

Indexes=SCI-EXPANDED, SSCI, A&HCI, CPCI-S, CPCI-SSH, BKCI-S, BKCI-SSH, ESCI, CCR-EXPANDED, IC Timespan=1990-2020

# 6 TS=burden

Indexes=SCI-EXPANDED, SSCI, A&HCI, CPCI-S, CPCI-SSH, BKCI-S, BKCI-SSH, ESCI, CCR-EXPANDED, IC Timespan=1990-2020

# 5 TS=("resilience factor*" or "protective factor*" or resource*)

Indexes=SCI-EXPANDED, SSCI, A&HCI, CPCI-S, CPCI-SSH, BKCI-S, BKCI-SSH, ESCI, CCR-EXPANDED, IC Timespan=1990-2020

# 4 TS=("post traumatic growth" or "posttraumatic growth" or "stress related growth")

Indexes=SCI-EXPANDED, SSCI, A&HCI, CPCI-S, CPCI-SSH, BKCI-S, BKCI-SSH, ESCI, CCR-EXPANDED, IC Timespan=1990-2020

# 3 TS=(resilien* or hardiness*)

Indexes=SCI-EXPANDED, SSCI, A&HCI, CPCI-S, CPCI-SSH, BKCI-S, BKCI-SSH, ESCI, CCR-EXPANDED, IC Timespan=1990-2020

# 2 TS=psychological

Indexes=SCI-EXPANDED, SSCI, A&HCI, CPCI-S, CPCI-SSH, BKCI-S, BKCI-SSH, ESCI, CCR-EXPANDED, IC Timespan=1990-2020

# 1 TS="mental health"

Indexes=SCI-EXPANDED, SSCI, A&HCI, CPCI-S, CPCI-SSH, BKCI-S, BKCI-SSH, ESCI, CCR-EXPANDED, IC Timespan=1990-2020

**eMethods 2. Search strategy for prepandemic comparative studies**

Systematic literature searches were conducted between July 13 and September 23, 2020 to find prepandemic comparative studies in the two bibliographic databases PubMed and PsycINFO. The searches comprised four key sets of terms in case of comparative data for studies conducted in the general population and five key sets for studies conducted among healthcare workers, patients, caregivers, and quarantined persons: 1) the name and shortcut of the respective assessment tool (eg, "Generalized Anxiety Disorder-7" OR "GAD-7"), 2) the respective country and demonym (eg, “Italy” OR “Italian”), 3) the term “representative”, 4) terms related to validation or psychometric studies, and 5) subgroup-specific terms. Students as another specific subgroup of the general population were not considered separately since a sufficient number of comparative studies could be found using the search terms for the general population. The terms were combined by the Boolean operators “AND” or “OR”, as appropriate.

Searches were performed in the order step 1 to step 7 until at least one matching result was found. Square brackets present an additional cluster for specific subgroups:

- Step 1: a search with all four [five in case of healthcare workers, patients, caregivers, or quarantined persons] key sets of search terms;
- Step 2: a search with the key sets 1), 2), 3) [and 5)];
- Step 3: a search with the key sets 1), 2), 4) [and 5)];
- Step 4: a search with the key sets 1), 2) [and 5)];
- Step 5: a search with the key sets 1), 3), 4) [and 5];
- Step 6: a search with the key sets 1), 3) [and 5];
- Step 7: a search with the key set 1) [and 5]

If no suitable result was found for subgroups (eg, healthcare workers) in this way, the data were compared with comparative data from the general population.

**General population (example search strategy for DASS-21, China, general population)**

Step 1:

- PubMed: ("DASS-21" OR "Depression Anxiety Stress Scale-21") AND (Chinese OR China) AND (psychometric OR norm OR validation OR validity) AND representative
- PsycINFO: ("DASS-21" OR "Depression Anxiety Stress Scale-21") AND (Chinese OR China) AND (psychometric OR norm OR validation OR validity) AND representative

Step 2:

- PubMed: ("DASS-21" OR "Depression Anxiety Stress Scale-21") AND (Chinese OR China) AND representative
- PsycINFO: ("DASS-21" OR "Depression Anxiety Stress Scale-21") AND (Chinese OR China) AND representative

Step 3:

- PubMed: ("DASS-21" OR "Depression Anxiety Stress Scale-21") AND (Chinese OR China) AND (psychometric OR norm OR validation OR validity)
- PsycINFO: ("DASS-21" OR "Depression Anxiety Stress Scale-21") AND (Chinese OR China) AND (psychometric OR norm OR validation OR validity)

Step 4:

- PubMed: ("DASS-21" OR "Depression Anxiety Stress Scale-21") AND (Chinese OR China)
- PsycINFO: ("DASS-21" OR "Depression Anxiety Stress Scale-21") AND (Chinese OR China)

**Healthcare workers (example search strategy for DASS-21, Singapore & India, healthcare workers)**

Step 1:

- PubMed: (“DASS-21” OR "Depression Anxiety Stress Scale-21") AND (Singapore OR Singaporean OR India OR Indian) AND (psychometric OR norm OR validation OR validity) AND representative AND ("health personnel" OR "health profession" OR "health worker" OR "health practitioner" OR "health provider" OR "health staff" OR physician OR doctor OR nurse] OR “nursing staff”)
- PsycINFO: (“DASS-21” OR "Depression Anxiety Stress Scale-21") AND (Singapore OR Singaporean OR India OR Indian) AND (psychometric OR norm OR validation OR validity) AND representative AND ("health personnel" OR "health profession" OR "health worker" OR "health practitioner" OR "health provider" OR "health staff" OR physician OR doctor OR nurse OR “nursing staff”)

Step 2:

- PubMed: (“DASS-21” OR "Depression Anxiety Stress Scale-21") AND (Singapore OR Singaporean OR India OR Indian) AND representative AND ("health personnel" OR "health profession" OR "health worker" OR "health practitioner" OR "health provider" OR "health staff" OR physician OR doctor OR nurse] OR “nursing staff”)
- PsycINFO: (“DASS-21” OR "Depression Anxiety Stress Scale-21") AND (Singapore OR Singaporean OR India OR Indian) AND representative AND ("health personnel" OR "health profession" OR "health worker" OR "health practitioner" OR "health provider" OR "health staff" OR physician OR doctor OR nurse OR “nursing staff”)

Step 3:

- PubMed: (“DASS-21” OR "Depression Anxiety Stress Scale-21") AND (Singapore OR Singaporean OR India OR Indian) AND (psychometric OR norm OR validation OR validity) AND ("health personnel" OR "health profession" OR "health worker" OR "health practitioner" OR "health provider" OR "health staff" OR physician OR doctor OR nurse] OR “nursing staff”)
- PsycINFO: (“DASS-21” OR "Depression Anxiety Stress Scale-21") AND (psychometric OR norm OR validation OR validity) AND (Singapore OR Singaporean OR India OR Indian) AND ("health personnel" OR "health profession" OR "health worker" OR "health practitioner" OR "health provider" OR "health staff" OR physician OR doctor OR nurse OR “nursing staff”)

Step 4:

- PubMed: (“DASS-21” OR "Depression Anxiety Stress Scale-21") AND (Singapore OR Singaporean OR India OR Indian) AND ("health personnel" OR "health profession" OR "health worker" OR "health practitioner" OR "health provider" OR "health staff" OR physician OR doctor OR nurse] OR “nursing staff”)
- PsycINFO: (“DASS-21” OR "Depression Anxiety Stress Scale-21") AND (Singapore OR Singaporean OR India OR Indian) AND ("health personnel" OR "health profession" OR "health worker" OR "health practitioner" OR "health provider" OR "health staff" OR physician OR doctor OR nurse OR “nursing staff”)

**Patients (example search strategy for DASS-21, China, psychiatric patients)**

Step 1:

- PubMed: (“DASS-21” OR " Depression Anxiety Stress Scale-21") AND (China OR Chinese) AND (validation OR validity OR psychometric OR norm) AND representative AND ("psychiatric patient" OR "mentally ill" OR "mental disease" OR "psychiatric disease" OR "psychiatric disorder" OR "mentally ill person" OR depressive OR depression OR "affective disorder" OR schizophrenia OR schizophrenic OR psychotic OR "anxiety disorder")
- PsycINFO: (“DASS-21” OR " Depression Anxiety Stress Scale-21") AND (China OR Chinese) AND (validation OR validity OR psychometric OR norm) AND representative AND ("psychiatric patient" OR "mentally ill" OR "mental disease" OR "psychiatric disease" OR "psychiatric disorder" OR "mentally ill person" OR depressive OR depression OR "affective disorder" OR schizophrenia OR schizophrenic OR psychotic OR "anxiety disorder")

Step 2:

- PubMed: (“DASS-21” OR " Depression Anxiety Stress Scale-21") AND (China OR Chinese) AND representative AND ("psychiatric patient" OR "mentally ill" OR "mental disease" OR "psychiatric disease" OR "psychiatric disorder" OR "mentally ill person" OR depressive OR depression OR "affective disorder" OR schizophrenia OR schizophrenic OR psychotic OR "anxiety disorder")
- PsycINFO: (“DASS-21” OR " Depression Anxiety Stress Scale-21") AND (China OR Chinese) AND representative AND ("psychiatric patient" OR "mentally ill" OR "mental disease" OR "psychiatric disease" OR "psychiatric disorder" OR "mentally ill person" OR depressive OR depression OR "affective disorder" OR schizophrenia OR schizophrenic OR psychotic OR "anxiety disorder")

Step 3:

- PubMed: (“DASS-21” OR " Depression Anxiety Stress Scale-21") AND (China OR Chinese) AND (validation OR validity OR psychometric OR norm) AND ("psychiatric patient" OR "mentally ill" OR "mental disease" OR "psychiatric disease" OR "psychiatric disorder" OR "mentally ill person" OR depressive OR depression OR "affective disorder" OR schizophrenia OR schizophrenic OR psychotic OR "anxiety disorder")
- PsycINFO: (“DASS-21” OR " Depression Anxiety Stress Scale-21") AND (China OR Chinese) AND (validation OR validity OR psychometric OR norm) AND ("psychiatric patient" OR "mentally ill" OR "mental disease" OR "psychiatric disease" OR "psychiatric disorder" OR "mentally ill person" OR depressive OR depression OR "affective disorder" OR schizophrenia OR schizophrenic OR psychotic OR "anxiety disorder")

Step 4:

- PubMed: (“DASS-21” OR " Depression Anxiety Stress Scale-21") AND (China OR Chinese) AND ("psychiatric patient" OR "mentally ill" OR "mental disease" OR "psychiatric disease" OR "psychiatric disorder" OR "mentally ill person" OR depressive OR depression OR "affective disorder" OR schizophrenia OR schizophrenic OR psychotic OR "anxiety disorder")
- PsycINFO: (“DASS-21” OR " Depression Anxiety Stress Scale-21") AND (China OR Chinese) AND ("psychiatric patient" OR "mentally ill" OR "mental disease" OR "psychiatric disease" OR "psychiatric disorder" OR "mentally ill person" OR depressive OR depression OR "affective disorder" OR schizophrenia OR schizophrenic OR psychotic OR "anxiety disorder")

**eTable 3. Eligibility criteria for SARS-CoV-2 pandemic studies**

| **Criterion** | **Description** |
| --- | --- |
| **Population** | **Inclusion:**  general population, healthcare workers (eg, physicians, nurses), and different patients (eg, COVID-19 patients, geriatric patients, psychiatric patients, pregnant women) exposed to the SARS-CoV-2 pandemic (ie, survey period after the first officially registered SARS-CoV-2 case in the respective country based on national infection dates published by the World Health Organization)^2^; all ages and sexes; irrespective of health status, employment status, and country; diverse settings of study conduction eligible  **Exclusion:**  due to focus of this review on the SARS-CoV-2 pandemic, individuals exposed to other epidemic or pandemic infectious disease outbreaks (eg, SARS, MERS, Ebola, HIV, influenza); chronic infectious diseases (eg, HIV/AIDS, tuberculosis, hepatitis B and C, malaria) |
| **Intervention** | Intervention studies were not considered for this review (see Study design) |
| **Comparison** | If appropriate, available comparators within a survey study were selected for the pair-wise meta-analysis according to the eligibility criteria in eTable 4. |
| **Outcome** | Mental burden or psychological distress, with a broad range of eligible mental health outcomes, for example:   - anxiety symptoms and worrying; - depressive symptoms; - stress symptoms and perceived stress; - sleep-related symptoms; - general psychological distress; - posttraumatic stress symptoms; - etc. |
| **Study design** | **Inclusion:**  observational cross-sectional and longitudinal surveys investigating the effects of the SARS-CoV-2 pandemic on mental burden  **Exclusion:**  intervention studies |
| **Publication date** | No restrictions |
| **Publication language** | No restrictions (translation of non-English articles) |
| **Publication format** | No restrictions (except for exclusion of preprint articles) |

**eTable 4. Eligibility criteria for prepandemic comparative studies (for pairwise meta-analyses)**

| **Criterion** | **Description** |
| --- | --- |
| **Population** | **Inclusion:**  individuals not exposed to epidemic or pandemic infectious disease outbreaks or other macrostressors (eg, natural disasters like earthquake, tsunami); sample should be as comparable as possible to the respective sample in the pandemic study, with **four levels of comparability (step-wise selection process)**:   - level 1: representative sample in the same country; - level 2: identical population (general population, healthcare workers, or respective patient group) or, if applicable, identical subgroup (eg, university students as subgroup of general population) in the same country; - level 3: for the general population: sample from a primary-care setting, students with mean age ≥18 years (unless the pandemic study also contains individuals aged < 18 years), older individuals; for healthcare workers and patient groups: data from the general population in the same country; or - level 4: best available data in a similar country (identical continent; if possible, similar distribution of demographic and socioeconomic variables)   In case of two comparative studies sharing the same level of comparability and quality (see also eTable 8), but with different sample sizes, the study with larger sample size was preferred.  **Exclusion:**  individuals exposed to other epidemic or pandemic infectious disease outbreaks or other macrostressors (eg, disaster) |
| **Intervention** | Not relevant for comparative data |
| **Comparison^a^** | Control groups of included pandemic studies or control groups of intervention studies were also eligible if they fulfilled the criteria for types of populations and outcomes |
| **Outcome** | **Inclusion:**  assessment of the same outcome and use of the same outcome measure as in the pandemic study (eg, depression subscale of Depression, Anxiety, and Stress Scales-21 [DASS-21] if survey data in the pandemic study were measured using this tool); as the pairwise meta-analyses focused on four primary outcomes (see eTable 5), especially the assessment of these outcomes in the comparative studies was relevant  **Exclusion:**  other outcome or outcome measure than in the pandemic study |
| **Study design** | No restrictions |
| **Publication date** | No restrictions |
| **Publication language** | No restrictions |
| **Publication format** | No restrictions |

^a^ All prepandemic comparative studies selected for this review can be viewed as historical control groups. However, if available, comparative data reported within an included pandemic study, that were collected before the SARS-CoV-2 pandemic, were also eligible in case they fulfilled the above criteria.

**eTable 5. Eligibility criteria for inclusion in pairwise meta-analyses**

| **Criterion** | **Description** |
| --- | --- |
| Outcome | assessment of one of the four primary outcomes (anxiety symptoms, depressive symptoms, stress, sleep-related symptoms) |
| Assessment tool | validated assessment tool in the included pandemic study |
| Reported data | mean value, standard deviation and sample size for relevant outcome reported (or received on request from study authors) |
| Comparative data | prepandemic comparative data available (either as part of the pandemic study or found in a systematic search for comparative data) |

**eTable 6. Customized data extraction sheet for included pandemic and prepandemic comparative studies**

| **Category** | **Extracted data^a^** |
| --- | --- |
| Source | - full citation and contact details |
| Methods | - study design - survey period and modalities |
| Participants | - total number - country - demographic data (eg, age, gender) - studied population (eg, general population, healthcare workers, patients) and potential subgroups (eg, students within general population; specific groups of patients) - representativeness of the sample |
| Outcomes | - outcomes and time points (1) collected; (2) reported - for each outcome of interest: - cut-off value and scale range, if appropriate |
| Results | - number of participants analyzed - for the outcomes of interest for the descriptive analysis of risk and protective factors for mental health in each target group (ie, general population, healthcare workers, patients): - results on evidence and non-evidence for associations between demographic and psychosocial risk and protective factors for mental health (ie, anxiety and worrying, depression, posttraumatic stress, sleep, stress, general psychological distress) - with risk/protective factors being clustered by: - demographic factors (eg, age), - work-related factors (eg, work from home), - pandemic-specific factors (eg, COVID-related thoughts and behaviors), - factors associated with information/communication (eg, knowledge about COVID-19), - psychosocial factors (eg, history of stressful events), or - health-related factors (eg, self-perceived health) - analysis method used by the primary studies to investigate the respective risk or protective factor, clustered by: - correlation analysis, - regression analysis, - General Linear Model, or - comparison of means - statistical values found in the respective analysis (eg, odds ratio from regression analysis, correlation coefficient) - for the six mental health outcomes of interest that were considered in the narrative synthesis of prevalence rates (anxiety and worrying, depression, posttraumatic stress sleep, stress, general psychological distress): - prevalence rate as proportion above reported cut-off-value; in case of no reported cut-off-values, prevalence of symptoms considered at least of “moderate” severity; for binary answer modalities: rate of participants answering “yes” for presence of the respective mental health outcome - in case of several reported prevalence rates, extraction of the highest and the lowest reported rate - summary of prevalence rates reporting the highest and the lowest reported prevalence across all studies regarding the respective outcome and population (general population, healthcare workers, or patients, respectively) - for the four primary outcomes of interest that were considered in meta-analysis (anxiety and worrying, depression, stress, sleep-related symptoms): - sample size, - summary data (means, standard deviations [SDs])^b^, - whether high or low score is good (ie, by considering potential recoding of scales in the primary studies) |
| Miscellaneous aspects | - correspondence required |
| **For prepandemic comparative data** | |
| Source | - full citation and contact details |
| Methods | - study design |
| Participants | - total number - country - demographic data (eg, age, gender) - studied population (eg, general population, healthcare workers, patients) or eligible subgroup for comparison with pandemic data - representativeness of the sample |
| Outcomes | - for each outcome of interest: - means, SDs, and sample size* |

^a^ The data extraction sheet was pretested in five studies. In addition to Table 1, a detailed data extraction sheet can be requested from the corresponding author.

^b^ Contact with study authors to ask for summary outcome data if these were not reported in the publication.

**eTable 7. Modified quality assessment tool for included pandemic studies**

| Original item | Abbreviation of this item in eTable 12 | Reason for modification/reason for omission of the item in the table | Description of answer options |
| --- | --- | --- | --- |
| 1. Was the research question or objective in this paper clearly stated? | 1. Research question? | not modified/omitted | - Yes: goal in conducting this research described and easy to understand - No: goal in conducting this research not described or not easy to understand - Not reported: no research question stated - Not available: does not apply to this item |
| 2. Was the study population clearly specified and defined? | 2. Study population clearly defined? | not modified/omitted | - Yes: description of the sample population using demographics, location, and time period - No: no sufficient description - Not reported: does not apply to this item - Not available: does not apply to this item |
| 3. Was the participation rate of eligible persons at least 50%? | 3. Participation rate ≥ 50%? | not modified/omitted | - Yes: participation rate of eligible persons ≥ 50% - No: participation rate of eligible persons < 50% - Not reported: participation rate of eligible persons not reported - Not available: the participants were recruited through convenience sampling |
| 4. Were all the subjects selected or recruited from the same or similar populations (including the same time period)? Were inclusion and exclusion criteria for being in the study prespecified and applied uniformly to all participants? | 4. Selection criteria? | not modified/omitted | - Yes: inclusion and exclusion criteria were developed prior to recruitment or selection of the study population; the same underlying criteria were used for all subjects involved - No: inclusion and exclusion criteria were not developed prior to recruitment or selection of the study population; the underlying criteria were not used in the same way for all subjects involved - Not reported: no in- or exclusion criteria reported - Not available: does not apply to this item |
| 5. Was a sample size justification, power description, or variance and effect estimates provided? | 5. Sample size justification, power description, variance and effect estimates? | not modified/omitted | - Yes: a sample size justification, power description, or variance and effect estimates were provided - No: a sample size justification, power description, or variance and effect estimates were not provided - Not reported: sample size justification, power description, or variance and effect estimates were calculated but not reported - Not available: no sample size justification, power description, or variance and effect estimates were possible |
| 6. For the analyses in this paper, were the exposure(s) of interest measured prior to the outcome(s) being measured? | omitted | Since the exposure was not predictable, this question is not applicable. |  |
| 7. Was the time frame sufficient so that one could reasonably expect to see an association between exposure and outcome if it existed? | omitted | This question was not applicable either, because the psychological burden probably varies over time (eg, by increase or decrease of infection rates, aggravation or relaxation of containment measures et cetera). In any case it is not known whether a longer exposure leads to greater psychological burden, which is the starting point of the question. Furthermore, this item is only applicable to studies with repeated measurements, but not to other comparisons such as those of exposed groups with comparative samples. |  |
| 8. For exposures that can vary in amount or level, did the study examine different levels of the exposure as related to the outcome (eg, categories of exposure, or exposure measured as continuous variable)? | 8. Was the exposure (independent variable) clearly specified? | This item is based on the assumption of a linear relationship between exposure and effect. Normally, a study receives a better evaluation if it is conducted at several points in time or exposure levels, because the correlation becomes more visible. However, since in a linear relationship cannot be assumed for studies included in this review, the item was modified. | - Yes: exposure to the SARS-CoV-2 pandemic was clearly stated, that is, the study was conducted after the first SARS-CoV-2 infection was publicly reported (or in case of China, after (or before and after) the 20.01.2020) - No: exposure to the SARS-CoV-2 pandemic was not clearly reported - Not reported: does not apply to this item - Not available: does not apply to this item |
| 9. Were the exposure measures (independent variables) clearly defined, valid, reliable, and implemented consistently across all study participants? | 9. Was the exposure consistent across all study participants? | This question was modified in the sense that the heterogeneity of exposure is assessed, since in the case of the COVID-19 pandemic as exposure, significant differences over a longer period might occur, for example, through loosening or tightening of the initial restrictions or if the number of infections increases or decreases. A smaller survey period is therefore desirable in the sense of a more homogeneous sample. | - Yes: survey period < 4 weeks - No: survey period ≥ 4 weeks - Not reported: survey period was not clearly defined - Not available: does not apply to this item |
| 10. Was the exposure(s) assessed more than once over time? | omitted | The application of this question to the included studies would require assessing several times whether the SARS-CoV-2 pandemic exists. A reasonable use of this item is not possible. |  |
| 11. Were the outcome measures (dependent variables) clearly defined, valid, reliable, and implemented consistently across all study participants? | 11. Outcome measures clearly defined, valid, reliable, and implemented consistently? | not modified/omitted | - Yes: the outcome measures were clearly defined, valid, reliable, and implemented consistently - No: the outcome measures were not clearly defined, valid, reliable, and implemented consistently - Not reported: no outcome measures were reported - Not available: does not apply to this item |
| 12. Were the outcome assessors blinded to the exposure status of participants? | omitted | Blinding the studies with the SARS-CoV-2 pandemic (exposure) is not possible, that is, the question is not applicable. |  |
| 13. Was loss to follow-up after baseline 20% or less? | omitted | Since none of the studies reassessed the same sample, this question is not applicable. |  |
| 14. Were key potential confounding variables measured and adjusted statistically for their impact on the relationship between exposure(s) and outcome(s)? | omitted | Since in the included pandemic studies no direct comparison of exposed and non-exposed participants was conducted, a collection of confounding variables does not make sense and, thus, the question is not applicable. |  |

**eTable 8. Rating of comparability between pandemic and prepandemic comparative studies**

| **Quality rating^a^** | **Comparative data** |
| --- | --- |
| Level 1 | Representative data in the same country and using the same outcome measure |
| Level 2 | Data for the identical population (general population, healthcare workers, or respective patient group) or the respective subgroup (eg, university students as subgroup of general population) in the same country and using the same outcome measure |
| Level 3 | - For a pandemic study in the *general population*, data for - a sample from the primary-care setting; - students with mean age ≥ 18 years (unless the pandemic study also contains individuals aged < 18 years); or - older individuals   in the same country and using the same outcome measure   - For a pandemic study in *healthcare workers* or *patients*, data for the general population in the same country and using the same outcome measure |
| Level 4 | Best available comparative data in a similar country (identical continent; similar distribution of demographic and socioeconomic variables, if possible) |

^a^ Self-developed tool for sensitivity analysis; based on eligibility criteria for comparative data.

**eMethods 3. Further methodological details of this systematic review and meta-analyses**

**Study selection**

The study selection process for pandemic studies was performed in duplicate by two independent reviewers (NR, LG). Titles and abstracts were screened to immediately exclude irrelevant records before inspecting the eligibility of studies at full-text level. Any disagreements were resolved by discussion or by consulting a third reviewer (KL).

The study selection procedure for the prepandemic comparative studies was pretested for the comparative data of ten randomly selected pandemic studies. For ten studies, comparative data were selected by two independent reviewers (NR, LG), working independently. Based on large agreements, the further selection of prepandemic comparative studies according to the above-described eligibility criteria (see eTable 4) was performed by a single reviewer (NR or LG), respectively.

**Data analysis**

Based on the extracted data, a narrative synthesis of the included pandemic studies was carried out describing information about the sample (country, sample size, proportion of female participants, age, potential subgroups), the study conduction (eg, survey period), and the measured outcomes. Risk and protective factors for mental health were also narratively summarised.

*Pairwise meta-analyses* were performed for pandemic studies reporting validated assessment tools, comparing the reported measures of mental health (anxiety symptoms, depressive symptoms, stress, or sleep-related symptoms) during the SARS-CoV-2 pandemic with prepandemic data (see eTable 5), in order to objectify the mental burden attributable to the SARS-CoV-2 pandemic as stressor. In case of pandemic studies reporting prevalence rates of mental burden (as dichotomous variables), the respective continuous (summary) outcome data (ie, means and SDs) were requested from the study authors. If possible, mixed studies were included in the quantitative analysis if relevant subgroup data (eg, healthcare workers for respective meta-analysis) could be extracted separately or were obtained by contacting the study authors. The decision to perform pooled analyses of the included pandemic studies depended on the number of studies found (ie, at least two pandemic studies measuring the same outcome in the same population) and the clinical and methodological homogeneity of included studies (eg, same population; same outcome assessed).

Based on previous systematic reviews and meta-analyses concerning the mental health impact of the SARS-CoV-2 pandemic^3-5^, a considerable range of outcome measures was anticipated. Therefore, we calculated standardised mean differences (Hedges’ g). Given the limited availability of adequate comparative data in the general population and healthcare workers, we used identical comparators for several pairwise comparisons (eg, use of Zhou et al^192^ as comparative study for SAS-measured anxiety in China for seven pairwise comparisons). To account for this nested data structure (level 1: participants; level 2: pandemic studies; level 3: prepandemic comparators), we used three-level meta-analytic models (ie, multilevel meta-analyses) with the pandemic studies being clustered within prepandemic comparative studies.^6,7^ Based on the single use of comparators in the main analyses for patients across the primary outcomes, pairwise meta-analyses were conducted according to a classic random-effects model which constitutes a special case of the multilevel model.

In addition to the main analyses, two planned *sensitivity analyses* apiece were performed for the primary outcomes in the general population, healthcare workers, and patients, in order to test the robustness of the findings. One sensitivity analysis (1) referred to the quality of the included pandemic studies, by excluding studies rated with “poor quality” in the modified quality assessment tool (see eTable 7). Second, a sensitivity analysis (2) according to the level of comparability was conducted, by excluding pairwise comparisons rated with level-3 and level-4 quality (see eTable 8).

In order to explain the between-study heterogeneity, several *subgroup analyses* were performed for the general population, healthcare workers, and patients, respectively, if the data were available. First, subgroup analyses concerning the characteristics of study populations in the pandemic studies were performed:

1. age (ie, different age groups based on available data),
2. special risk or stressor exposure within the general population,
3. COVID-19 patient contact of healthcare workers (ie, low contact vs high contact),
4. different groups of patients.

Regarding the study characteristics of the included pandemic studies, the following subgroup analyses were performed:

1. study conduction in China vs outside of China,
2. sample size,
3. survey period (ie, survey start depending on when the first case of SARS-CoV-2 had been registered in the respective country; eg, Germany: January 28, 2020; for China: January 20, 2020 as date of first confirmed human-to-human transmission), and the
4. outcome measures used to assess the respective primary outcome of anxiety, depressive symptoms, stress, or sleep-related symptoms.

With respect to comparative study characteristics, subgroup analyses comprised the

1. sample size and the
2. publication year (ie, in order to control for potential cohort effects) of prepandemic studies.

Finally, a subgroup analysis was conducted concerning the

1. relationship of sample sizes between the pandemic and comparative studies.

Relevant subgroups were selected during the review development process depending on the available data. Depending on the multiple uses of prepandemic comparative studies in the main analyses, subgroup analyses for the general population and healthcare workers were also performed based on multilevel modelling. Due to the limited number of studies per subgroups, which might lead to an imprecise estimation of heterogeneity within subgroups, subgroup meta-analyses were conducted assuming a common-between study variance (Tau^2^) within the subgroups.^8^

All statistical analyses were performed using R 4.0.3 (package meta, version 4.15-1).^9^ For the general population and healthcare workers, we performed both classic random-effects pairwise meta-analyses as well as multilevel meta-analyses and compared their findings. However, the respective tables (see Table 3 and 4 in Results) and forest plots (see Results and Supplementary material 4 and 5) only contain the results of multilevel modelling. Following the recommendations in the literature^10,11^, statistical heterogeneity was described with I^2^, Tau^2^, and Chi^2^ test as well as 95% prediction intervals (only conducted for meta-analyses with at least k=4 studies) to present the extent of between-study variation.

Confidence intervals (CIs) from a meta-analysis describe the uncertainty in the location of the mean (ie, centre of distribution) of effects in different studies, but do not refer to the width of distribution, that is, the heterogeneity among studies (eg, tight 95% CI around the effect estimate due to many studies in a meta-analysis even if there is a large heterogeneity).^10^ Prediction intervals, however, describe the level of heterogeneity among studies and allow to give a more comprehensive summary of a meta-analysis when being presented together with the effect estimate and their 95% CI.^10^ They *predict* the possible effect in a new study that is similar to the studies in a meta-analysis and, in this review, can be interpreted as the expected range of true effects of the SARS-CoV-2 pandemic on mental burden in similar studies.^10^ In all analyses (main, subgroup, and sensitivity analyses), the between-study variation (Tau^2^) was estimated using a restricted maximum likelihood (REML) approach which has been recommended by Cochrane.^10^

**Quality assessment of included pandemic studies**

To assess the quality of the included pandemic studies, the Quality Assessment Tool for Observational Cohort and Cross-sectional Studies of the National Heart, Lung, and Blood Institutes^12^, adapted for this review, was used. The assessment was performed by two independent reviewers (NR, LG). The exposure (independent

variable) was defined as the presence of the stress factor "SARS-CoV 2 pandemic", that is, a survey period *after* the public report of the first nationwide COVID-19 patient (for China: after January 20, 2020, as human-to-human transmission was officially confirmed).^13^

Some items of the quality assessment tool were modified to meet the requirements of the studies included in the review. Items, that were not applicable to the included studies, are not listed.

The following items were added:

1. Selection bias/possible selection bias because of insufficient information on the sample recruitment: A selection bias exists, for example, if the recruitment of study participants was carried out using snowball or other convenience sampling methods. In this case, it must be assumed that only particularly stressed persons may have responded.
2. No/insufficient details on survey period: If there were no details on the survey period, this comment was added.
3. No cut-off values and/or scale range for the outcome assessment reported: If there were no details on the scale range and/or the cut-off value for the relevant outcome measure, this comment was added. If the scale-range was reported but no cut-off value, the comment was provided with an asterisk (*).
4. No validated assessment measure for the outcome/outcome measure not clearly defined: If the used assessment tool for the relevant outcome(s) was not a previously validated assessment tool (ie, before the current SARS-CoV-2 pandemic), this comment was added.
5. Insufficient description of the study sample: If there was no or not sufficient information on the study

sample, that is, on the in- and exclusion criteria or on demographic information (age, gender, region,

occupation), this comment was added.

1. Insufficient justification of the summary of the outcomes and g) reporting bias: for some studies, additional comments were added suiting only for one or very few studies.

The quality of studies was rated as high, fair, or poor, with the criteria for the overall rating presented in the following.

**Table eMethods 3. Criteria for overall quality rating of pandemic studies**

| **Overall Rating** | **Criteria** |
| --- | --- |
| HIGH | - no selection bias + validated assessment tool AND - <2 of the original items rated as “not reported” or “no” |
| FAIR | - selection bias and/or no validated assessment tool AND - <2 of the original items rated as “not reported” AND - <3 of the original items rated as “no” AND - <3 of the original items rated as other than “yes” or “not available” |
| POOR | - selection bias and/or no validated assessment tool + ≥3 of the original items rated as “not reported”/”no” OR - >1 of the original items rated as “not reported” OR - ≥3 of the original items rated as “no” OR - ≥3 of the original items rated as other than “yes” or “not available” |

Changes were made concerning the following items of the NIH tool^11^:

- Question 8 ("For exposures that can vary in amount or level, did the study examine different levels of the exposure as related to the outcome [eg, categories of exposure, or exposure measured as continuous variable]?”): This item was modified, since it is based on the assumption of a linear relationship between exposure and effect. Usually, a study receives a better evaluation if it is conducted at several points in time or exposure levels, because the correlation becomes more visible. However, since in a linear relationship cannot be assumed for studies included in this review, the item was modified to "Was the exposure (independent variable) clearly specified?".
- Question 9 ("Were the exposure measures [independent variables] clearly defined, valid, reliable, and implemented consistently across all study participants?"): This question was modified in the sense that the heterogeneity of exposure is assessed. In the case of the SARS-CoV-2 pandemic as exposure, significant differences over a longer period might occur, for example, through loosening or tightening of the initial restrictions or if the number of infections increases or decreases. Therefore, a smaller survey period in the sense of a more homogeneous sample is more desirable and the question was changed to: "Was the exposure consistent across all study participants?".

The following questions are not listed in eTable 7.

- Question 6 (“For the analyses in this paper, were the exposure[s] of interest measured prior to the outcome[s] being measured?”): Since the exposure was not predictable, this question is not applicable.
- Question 7 (“Was the timeframe sufficient so that one could reasonably expect to see an association between exposure and outcome if it existed?”): This question was not applicable either because the psychological burden probably varies over time (eg, by increase of decrease of infection rates, aggravation or relaxation of containment measures etc.). In any case, it is not known whether a longer exposure leads to greater psychological burden, which is the starting point of the question. Furthermore, this item is only applicable to studies with repeated measurements but not to other comparisons, such as those of exposed groups with comparative samples.
- Question 10 (“What the exposure(s) assessed more than once over time?”): The application of this question to the included pandemic studies would require a repeated assessment of the existence of the SARS-CoV-2 pandemic exists. Therefore, a reasonable use of this item is not possible.
- Question 12 (“Were the outcome assessors blinded to the exposure status of participants?”): Blinding the studies with the SARS-CoV-2 pandemic as exposure is not possible, thus, the question is not applicable.
- Question 13 (“Was the loss to follow-up after baseline 20% or less?”): Since none of the included studies reassessed the same sample, this question is not applicable.
- Question 14 (“Were key potential confounding variables measured and adjusted statistically for their impact on the relationship between exposure[s] and outcome[s]?”): Since no direct comparison of exposed and non-exposed participants was conducted in the included pandemic studies, the collection of confounding variables seems not reasonable and, thus, the question is not applicable.

A more detailed guideline for the application of the modified instrument is available from the authors on request.

**References**

The reference numbers for the included pandemic studies and the prepandemic comparative studies are identical to the reference numbers used in the publication.

1. Stroup DF, Berlin JA, Morton SC, et al, for the Meta-analysis Of Observational Studies in Epidemiology (MOOSE) Group. Meta-analysis of observational studies in epidemiology. A proposal for reporting. *JAMA*. 2000;283(15):2008-2012. doi: 10.1001/jama.283.15.2008.
2. World Health Organization. COVID-19 global data. 2020. https://covid19.who.int/WHO-COVID-19-global-data.csv. Accessed August 20, 2020.
3. Gilan D, Röthke N, Blessin M, et al. Psychomorbidity, resilience, and exacerbating and protective factors during the SARS-CoV-2-pandemic. A systematic literature review and results from the German COSMO-PANEL. *Dtsch Arztebl Int.* 2020;117:625-632.
4. Krishnamoorthy Y, Nagarajan R, Saya GK, Menon V. Prevalence of psychological morbidities among general population, healthcare workers and COVID-19 patients amidst the COVID-19 pandemic: a systematic review and meta-analysis. *Psychiatry Res.* 2020;293:113382.
5. Luo M, Guo L, Yu M, Jiang W, Wang H. The psychological and mental impact of coronavirus disease 2019 (COVID-19) on medical staff and general public - a systematic review and meta-analysis. *Psychiatry Res.* 2020;291:113190.
6. Van den Noortgate W, López-López JA, Marín-Martínez F, Sánchez-Meca J. Three-level meta-analysis of dependent effect sizes. *Behav Res.* 2013;45(2):576-594.
7. Assink M, Wibbelink CJM. Fitting three-level meta-analytic models in R: a step-by-step tutorial. *TQMP.* 2016;12(3):154-174.
8. Schwarzer G, Carpenter JR, Rücker G. Heterogeneity and meta-regression. In: Schwarzer G, Carpenter JR, Rücker G, eds. *Meta-Analysis with R.* Basel, Switzerland: Springer International Publishing; 2015: 85-104.
9. Balduzzi S, Rücker G, Schwarzer G. How to perform a meta-analysis with {R}: a practical tutorial. *Evidence-Based Mental Health*. 2019;22:153-160.
10. IntHout J, Ioannidis JPA, Rovers MM, Goeman JJ. Plea for routinely presenting prediction intervals in meta-analysis. *BMJ Open.* 2016;6(7):e010247.
11. Deeks JJ, Higgins JPT, Altman DG. Chapter 10: analysing data and undertaking meta-analyses. In: Higgins JPT, Thomas J, Chandler J, et al., eds. *Cochrane Handbook for Systematic Reviews of Interventions version 6.1* (updated September 2020): Cochrane, 2020.
12. National Heart, Lung, and Blood Institute. Quality Assessment Tool for Observational Cohort and Cross-Sectional Studies. 2014. [https://www.nhlbi.nih.gov/health-topics/study-quality-assessment-tools](about:blank). Accessed October 2, 2020.
13. National Health Commission of the People’s Republic of China. Timeline of China releasing information on COVID-19 and advancing international cooperation (Jan 20). 2020. http://en.nhc.gov.cn/2020-04/06/c_78861_3.htm. Accessed October 10, 2020.
