# Supplementary material for: Mental burden and its risk and protective factors during the early phase of the SARS-CoV-2 pandemic: systematic review and meta-analyses
Source: Global Health. 2021 Mar 29;17:34. doi: 10.1186/s12992-021-00670-y (PMC8006628; doi:10.1186/s12992-021-00670-y)
Supplement: Supplementary file 2 — Additional file 2: Results of the systematic review with meta-analyses. eTable 9. Details on number of included (pandemic and comparative) studies. eTable 10. Study characteristics of the prepandemic comparative studies. eTable 11. Cut-off values reported in included pandemic studies. eResults 1. Forest plots of main analyses. eTable 12. Quality assessment of included pandemic studies. eTable 13. Assessment of level of comparability between pandemic and prepandemic comparative studies. eResults 2. Forest plots of sensitivity analyses. eResults 3. Detailed results of subgroup analyses. eTable 14. Risk factors in the general population, healthcare workers, and patients. eTable 15. Protective factors in the general population, healthcare workers, and patients. [file 12992_2021_670_MOESM2_ESM.docx]

**Additional file 2**

Mental burden and its risk and protective factors during the early phase of the SARS-CoV-2 pandemic: systematic review and meta-analyses

Angela M Kunzler*, Nikolaus Röthke*, Lukas Günthner, Jutta Stoffers-Winterling, Oliver Tüscher, Michaela Coenen, Eva Rehfuess, Guido Schwarzer, Harald Binder, Christine Schmucker, Jörg Meerpohl, Klaus Lieb

**eTable 9.** Details on number of included (pandemic and comparative) studies

**eTable 10.** Study characteristics of the prepandemic comparative studies

**eTable 11.** Cut-off values reported in included pandemic studies

**eResults 1.** Forest plots of main analyses

**eTable 12.** Quality assessment of included pandemic studies

**eTable 13.** Assessment of level of comparability between pandemic and prepandemic comparative studies

**eResults 2.** Forest plots of sensitivity analyses

**eResults 3.** Detailed results of subgroup analyses

**eTable 14.** Risk factors in the general population, healthcare workers, and patients

**eTable 15.** Protective factors in the general population, healthcare workers, and patients

**eTable 9. Details on number of included (pandemic and comparative) studies**

| **Number^a^** | **General population** | **Healthcare workers** | | | **Patients** | **Mixed^b^** | **Total** |
| --- | --- | --- | --- | --- | --- | --- | --- |
| **Systematic review** | | | | | | | |
| **Pandemic studies** | | | | | | | |
| Studies | 50 | 30 | | | 7 | 17 | 104 (108 reports) |
| Samples^c^ | 64 | 42 | | | 14 | 4 | 124 |
| Participants: studies (samples)^d^ | 150455 (163191) | 26101 (35075) | | | 5159 (5998) | 26546 (3997) | 208261 |
| **Pairwise meta-analyses** | | | | | | | |
| **Pandemic studies** | | | | | | | |
| Studies | 23 | 13 | | 4 | | 3 | 43 (47 reports) |
| Samples^c^ | 29 | 14^e^ | | 7^f^ | |  | 50 |
|  | General population: 20 | COVID-19 patient exposure: 7 | | Psychiatric patient: 3 | |  |  |
|  | Students: 5 |  |  | COVID-19 patients: 2 | |  |  |
|  | Risk groups (eg, caregiver): 3 | No COVID-19 patient exposure: 7 | | Pregnant women: 2 | |  |  |
|  | Others: 1 |  |  |  |  |  |  |
| Participants^g^ | 61160 | 5508 | | 4945 | |  | 71613 |
| **Comparative studies** | | | | | | | |
|  |  | |  |  | | **GP studies (samples) used for comparison with HCW/P** |  |
| Studies | 29 | 10 | | 9 | | 1/6 | 48 (48 reports)^h^ |
| Samples | 29 | 10 | | 10^h^ | | 1/6 | 49^h,i^ |
| Participants | 118068 | 10589 | | 11911 | | 37209 | 140568^h^ |

^a^ Number of included studies, samples, and participants in review and meta-analyses for pandemic studies (each population) and comparative studies.

^b^ Mixed: eg, general population and healthcare workers.

^c^ Mixed samples were immediately allocated to samples in healthcare workers or patients, if possible.

^d^ Participants of mixed samples were also immediately allocated to samples in healthcare workers or patients, if possible (ie, n=3997 participants of mixed samples in systematic review refer to the four remaining samples that could not be assigned).

^e^ two samples in Cai W et al^101^ were combined to one.

^f^ two samples in Liu X et al^42^ were combined to one.

^g^ across four primary outcomes for which pairwise meta-analyses were performed (anxiety, depression, stress, sleep-related symptoms) with single counting.

^h^ total number only refers to three first columns.

^i^ two different samples used in case of Wang et al^185^.

**eTable 10. Study characteristics of the prepandemic comparative studies**

| Comparative study | **Pandemic study to compare with** | **Country** | **Study sample used: sample size; female: No (%); age: M (SD)** | **Study design** | **Survey period** | **Study population** | **Study subsample/**  **data used** | **Relevant assessment tool(s)** |
| --- | --- | --- | --- | --- | --- | --- | --- | --- |
| Alosaimi et al (2018)^148^ | Badahdah et al (2020)^98^ | Saudi Arabia | 582; 168 (29%); 46.9 (7.9) | cross-sectional, observational | November 2014-March 2015 | physicians | NA | PSS |
| Balestrieri et al (2010)^149^ | Soraci et al (2020)^78^ | Italy | 21644; 13203 (61%); NA (mode: 45-65 years) | cross-sectional, observational | NA | primary care sample | NA | HADS |
| Basta et al (2019)^150^ | Tsipropoulou et al (2020)^82^ | Greece | 2771; 1496 (54%); NA (Md=21, IQR=18-23 years) | cross-sectional, observational | March-April 2016 | young general population (representative) | NA^a^ | GAD-7, PHQ-9 |
| Bilgel et al (2010)^151^ | Özdin et al (2020)^69^ | Turkey | 1102; 642 (58%); 20.0 (1.5) | cross-sectional, observational | NA | students | NA | HADS |
| Bonfiglio et al (2016)^152^ | Germani et al (2020)^51^; Iasevoli et al (2020, controls)^135^;  Iasevoli et al (2020, caregivers)^135^ | Italy | 337; 249 (74%); 28.0 (9.6) | cross-sectional, observational (validation study) | NA | general population | non-clinical sample | PSS |
| Bottesi et al (2015)^153^ | Mazza et al (2020)^63^ | Italy | 417; 238 (57%); 36.4 (13.7) | cross-sectional, observational | NA | general population | NA | DASS-21 |
| Cai S et al (2018)^154^ | Cai W et al (2020)^101^ | China | 1608; 1608 (100%); 32.3 (8.6) | cross-sectional, observational | March-April 2015 | healthcare workers | NA | SCL-90 |
| Carlucci et al (2018)^155^ | Germani et al (2010)^51^ | Italy | 2938; 1667 (56%), 36.3 (20.3) | cross-sectional, observational | NA | general population | NA | STAI-Y |
| Carta et al (2013)^156^ | Iasevoli et al (2020, control group)^135^; Iasevoli et al (2020, patient group)^135^ | Italy | 1200; 618 (52%), NA (mode: 30-44 years) | cross-sectional, observational | November 2011-August 2012 | general population | NA | PHQ-9 |
| Choueiry et al (2016)^157^ | Babahdah et al (2020)^98^ | Lebanon | 462; 322 (70%); 21.2 (1.8) | cross-sectional, observational | September 2013-May 2014 | students | NA | GAD-7 |
| Chung et al (2010)^158^ | Hao F et al (2020)^132^ | China | 91; 73 (80%); 48.3 (9.5) | longitudinal, observational | NA | patients with major depressive disorder | baseline data | ISI |
| Dadfar et al (2019)^159^ | Zhang SX et al (2020c)^121^ | Iran | 157; 61 (39%); 25.5 (8.0) | cross-sectional, observational (validation study) | NA | students | NA | PHQ-2 |
| Dong et al (2017)^160^ | Xiao et al (2020)^117^; Wang S (2020)^115^; Wu K (2020)^116^ | China | 4951; 4667 (94%); 28 (6) | cross-sectional, observational | May-December 2015 | nurses | NA | PSQI |
| Ertekin et al (2018)^161^ | Durankus et al (2020)^125^ | Turkey | 99; 99 (100%); 27.9 (5.1) | randomized controlled intervention study | October 2014-December 2015 | pregnant women | control group | BDI |
| Fleishman et al (2007)^162^ | Olagoke et al (2020)^146^ | USA | 11109; 7854 (70%); NA (mode: 18-40 years) | cross-sectional, observational | 2004 | general population (representative) | NA | PHQ-2 |
| García-Campayo et al (2012)^163^ | González-Sanguino et al (2020)^52^ | Spain | 110; 80 (73%); 48.0 (16.1) | cross-sectional, observational, controlled | NA | primary care sample | control group | GAD-2 |
| Ho R et al (2016)^164^ | Liu X et al (2020a)^42^ | China | 51; 20 (39%); 52.4 (9.6) | randomized controlled intervention study | N/A | schizophrenia patients | Tai-chi group, baseline data | PSS |
| Hossain et al (2019)^165^ | Sakib et al (2020)^74^ | Bangladesh | 1140; 479 (42%); 19.5 (0.9) | longitudinal, observational | April 2016-July 2016 | students | follow-up data^a^ (baseline data not available) | PHQ-9 |
| Huang F et al (2020)^166^ | Guo et al (2020)^131^ | China | 9507; 4658 (49%); 47.5 (14.1) | cross-sectional, observational | 2015 | general population | NA | PSS |
| Ivziku et al (2019)^167^ | Iasevoli et al (2020, patients)^135^ | Italy | 80; 35 (44%); 76.4 (7.3) | cross-sectional, observational | November 2016-October 2017 | COPD patients | patient group | GAD-7 |
| Jeyagurunathan et al (2017)^168^ | Iasevoli et al (2020, caregivers)^135^ | Singapore | 339; 229 (70%); 49.7 (13.2) | cross-sectional, observational | July 2014-May 2015 | caregivers of mentally ill people | NA | GAD-7, PHQ-9 |
| Jin et al (1986)^169, b^ | Tian et al (2020)^81, b^ | China | 1388; NA; NA | cross-sectional, observational | NA | general population | NA | SCL-90 |
| Lee K et al (2017)^170^ | Yuan R et al (2020, parents of children hospitalized during pandemic)^89^; Yuan R et al (2020, parents of children not hospitalized during pandemic)^89^ | China | 8284; 4208 (51%), NA (mode: ≥60 years) | cross-sectional, observational | NA | general population | NA | HADS |
| Lin R et al (2018)^171^ | Hao F (2020, controls)^132^; Tan W et al (2020)^80^ | China | 1013; 625 (62%); 20.4 (1.5) | cross-sectional, observational | NA | students | sample 2 | ISI |
| Liu H et al (2009)^172^ | Liu X et al (2020a)^42^ | China | 101; 35 (35%); 34.1 (10.0) | cross-sectional, observational | January-May 2007 | schizophrenia patients | NA | HAMD |
| Liu R et al (2016)^173^ | Ma et al (2020)^62^ | China | 4604; NR; NR | cross-sectional, observational | 2012-2013 | general population | no hypertension group | PSQI |
| Liu X et al (2020)^174^ | Wang S et al (2020)^115^; Zhu J et al (2020)^122^; Wu K et al (2020, C19-group)^116^, Wu K et al (2020, nC19-group)^116^ | China | 242; NA; NA | cross-sectional, observational | November 2018-January 2019 | nurses | NA | SDS |
| Löwe et al (2008)^175^ | Amerio et al (2020)^97^; Bäuerle et al (2020)^45^; Consolo et al (2020)^103^; Iasevoli et al (2020; control group)^135^ | Germany | 5030; 2696 (54%); 48.4 (18.0) | cross-sectional, observational | May 2006-June 2006 | general population (representative) | NA | GAD-7 |
| Löwe et al (2010)^176^ | Bäuerle et al (2020)^45^; González-Sanguino et al (2020)^52^; Voitsidis et al (2020)^83^ | Germany | 5010; NA; NA | cross-sectional, observational | May 2006-June 2006 | general population (representative) | NA | PHQ-2 |
| Lu S et al (2018)^177^ | Hao F et al (2020; controls)^132^; McKay et al (2020)^64^; Tan W et al (2020)^80^; Wang C et al (2020b)^85^ | China | 13208; 8223 (62%); 19.7 (1.8) | cross-sectional, observational | NA | students | NA | DASS-21 |
| Lu W et al (2017)^178^ | Guo et al (2020, controls)^131^; Guo et al (2020, patients)^131^; Chang et al (2020)^49^; Zhou et al (2020)^93^ | China | 1096; 395 (36%); 18.3 (0.7) | cross-sectional, observational | January-December 2011 | students | NA | GAD-7 |
| Paparrigopoulos et al (2010)^179^ | Voitsidis et al (2020)^83^ | Greece | 1005; 522 (52%); NA (mode: >65 years) | cross-sectional, observational | NA | general population (representative) | NA | AIS |
| Pereira-Lima et al (2014)^180^ | Zhang SX et al (2020c)^121^ | Brazil | 305; 146 (48%); 28 (2.5) | cross-sectional, observational | NA | physicians | NA | GAD-2 |
| Ramón-Arbués et al (2019)^181^ | Odriozola-González et al (2020)^66^ | Spain | 1055; 744 (71%); 21.7 (5.2) | cross-sectional, observational | September 2018-January 2019 | students | NA | DASS-21 |
| Sasaki et al (2020)^182^ | Chew et al (2020)^102^; Tan B et al (2020)^114^ | Vietnam | 933; 806 (85%); 33.1 (6.8) | cross-sectional, observational | August-September 2018 | nurses | NA | DASS-21 |
| Schmidt et al (2015)^183^ | Amerio et al (2020)^97^ | Switzerland | 220; 124 (56%); NA (mode: 30-39 years) | cross-sectional, observational | 2011 | physicians | NA | ISI |
| Sinclair et al (2012)^184^ | Tull et al (2020)^79^ | USA | 499; 259 (52%); 44.7 (16.3) | cross-sectional, observational | NA | general population (representative) | NA | DASS-21 |
| Wang K et al (2016)^185^ | Hao F et al (2020; patient group)^132^; Ma et al (2020)^62^ | China | study 1: 1815; 1124 (62%), 18.8 (0.8);  study 2: 166; 79 (48%); 42.8 (9.6) | cross-sectional, observational, study 2 controlled | NA | students (study 1); schizophrenia patients (study 2) | students sample (study 1); schizophrenia patients (study 2) | DASS-21 |
| Wang W et al (2014)^186^ | Chang et al (2020)^49^; Guo et al (2020, controls)^131^; Guo et al (2020, patients)^131^; Zhou SJ et al (2020)^93^ | China | 1045; 679 (65%); 47.1 (16.2) | cross-sectional, observational | June 2011-January 2012 | general population (validation study) | NA | PHQ-9 |
| Wang WL et al (2020)^187^ | Liu X et al (2020a)^42^ | China | 207; 70 (34%); 42.3 (10.0) | cross-sectional, observational | July 2017-May 2018 | schizophrenia patients | NA | PSQI |
| Wang X et al (1999)^188^ | Lei et al (2020)^57^; Wang Y et al (2020)^87^ | China | 1158; NR; NR | cross-sectional, observational | NA | general population | NA | SAS |
| Wang Y et al (2019)^189^ | Liu X et al (2020a)^42^ | China | 99; 28 (28%); 39.8 (10.1) | randomized controlled intervention study | January 2017-February 2018 | schizophrenia patients | Buspirone group (larger sample) | HAMA |
| Wu Y et al (2020)^127, c^ | Wu Y et al (2020)^127, c^ | China | 2839; 2839 (100%); NA (mode: age <35 years) | cross-sectional, observational | January 2020 | pregnant women | group 1 | EPDS |
| Yang X et al (2016)^190^ | Cai X et al (2020)^123^ | China | 8178; 5016 (61%); 21.1 (1.4) | cross-sectional, observational | September 2012-July 2013 | students | study 1 | SAS |
| Yildirim et al (2018)^191^ | Satici et al (2020)^75^ | Turkey | 250; 120 (48%); 30 (9.3) | cross-sectional, observational, controlled | NA | general population | controls | SAS |
| Yu B et al (2019)^192^ | Cai X et al (2020)^123^; Lei et al (2020)^57^; Wang Y et al (2020)^87^ | China | 18994; 8661 (46%); 42.2 (11.9) | cross-sectional, observational | January 2013-December 2016 | general population | NA | SDS |
| Zhou et al (2016)^193^ | Mo et al (2020)^108^; Pu et al (2020)^109^;Wang S et al (2020)^115^; Wu K et al (2020, C19 group)^116^; Wu K et al (2020, nC19-group)^116^; Xiao et al (2020)^117^; Zhu J et al (2020)^122^ | China | 1129; 654 (58%); 38.0 (7.7) | cross-sectional, observational | NA | physicians | NA | SAS |

Abbreviations: AIS, Athens Insomnia Scale; BDI, Beck Depression Inventory, C19=COVID-19; DASS-21, Depression Anxiety Stress Scale-21; EPDS, Edinburgh Postnatal Depression Scale; GAD-2/7, Generalized Anxiety Disorder-2/7; HADS, Hospital Anxiety and Depression Scale; HAMA, Hamilton Anxiety Rating Scale; HAMD, Hamilton Depression Rating Scale; IRQ, interquartile range; ISI, Insomnia Severity Index; Md, median; NA, not applicable; nC19, non-COVID-19; NR, not reported; PHQ-2/9, Patient Health Questionnaire-2/9; PSQI, Pittsburgh Sleep Quality Index; PSS, Perceived Stress Scale; SAS, Self-Rating Anxiety Scale; SCL-90, Symptom Checklist-90; SDS, Self-Rating Depression Scale; STAI-Y, State Trait Anxiety Inventory-Y.

**^a^** received on request.

^b^ comparative study mentioned in Tian et al (2020)^81^.

^c^ comparison part of the study.

**eTable 11. Cut-off values reported in included pandemic studies**

| **Assessment tool** | **Study: cut-off value, scale range/scoring** |
| --- | --- |
| **AIS** | Voitsidis et al (2020)^83^: NA, NA |
| **“Anxiety scale“** | Xu J et al (2020)^118^: ≥8, NA |
| **BAI** | Durankus et al (2020)^125^: NA, 0-63 |
| **BDI** | Durankus et al (2020)^125^: NA, 0-63 |
| **BDI-II** | Bacon et al (2020)^44^: NA, 21-63 |
| **BIP-Q5** | Perez-Fuentes et al (2020)^70^: NA, NA |
| **CES-D** | Huang Y et al (2020)^134^: ≥29, 0-60 |
| **CoVGAD-7** | McKay et al (2020)^64^: NA, NA |
| **CPDI** | Jahanshahi et al (2020)^54^: ≥28, 0-100;  Qiu et al (2020)^41^: ≥28, 0-100 |
| **DASS-21 anxiety subscale** | Chew et al (2020)^102^: ≥8, 0-42;  Hao F et al (2020)^132^: ≥10, NA;  Ma et al (2020)^62^: NA, NA;  Mazza et al (2020)^63^: ≥10, 0-42;  McKay et al (2020)^64^: NA, NA;  Odriozola-Gonzalez et al (2020)^66^: NA, NA;  Ozamiz-Etxebarria (2020)^68^: NA, NA;  Satici et al (2020)^75^: NA, NA;  Tan B et al (2020)^114^: ≥8, NA;  Tan W et al (2020)^80^: NA, NA;  Tull et al (2020)^79^: NA, 0-21;  Wang C et al (2020a)^84^: ≥10, 0-42 |
| **DASS-21 depression subscale** | Chew et al (2020)^102^: ≥10, 0-42;  Hao F et al (2020)^132^: ≥14, NA;  Ma et al (2020)^62^: NA, NA;  Mazza et al (2020)^63^: ≥13, 0-42;  McKay et al (2020)^64^: NA, NA;  Odriozola-Gonzalez et al (2020)^66^: NA, NA;  Ozamiz-Etxebarria et al (2020)^68^: NA, NA;  Satici et al (2020)^75^: NA, NA;  Tan B et al (2020)^114^: ≥10, NA  Tan W et al (2020)^80^: NA, NA;  Wang C et al (2020a)^84^: ≥13, 0-42 |
| **DASS-21-stress subscale** | Chew et al (2020)^102^: >14, 0-42;  Hao F et al (2020)^132^: ≥19, NA;  Ma et al (2020)^62^: NA, NA;  Mazza et al (2020)^63^: ≥19, 0-42;  McKay et al (2020)^64^: NA, NA;  Odriozola-Gonzalez et al (2020)^66^: NA, NA;  Ozamiz-Etxebarria et al (2020)^68^: NA, NA;  Satici et al (2020)^75^: NA, NA;  Tan B et al (2020)^114^: >14, NA;  Tan W et al (2020)^80^: NA, NA;  Wang C et al (2020a)^84^: ≥19, 0-42 |
| **“Depression score“** | Xu J et al (2020)^118^: ≥8, NA |
| **Developed by study authors** | Abdessater et al (2020)^94^: NA, NA;  Ahmad et al (2020)^43^: yes, binary item (yes, no);  Ahmed et al (2020)^95^: yes, binary item (yes, no);  Alhaj et al (2020)^96^: yes, binary item (yes, no);  Bäuerle et al (2020)^45^: ≥5, 1-7;  Bohlken et al (2020)^99^: ≥4, 1-5;  Buzzi et al (2020)^47^: NA, NA (nothing, little, moderately, a lot);  Büntzel et al (2020)^130^: NA, NA;  Cai H et al (2020)^100^: ≥2, 0-3;  Chew et al (2020)^102^: ≥„mild”, NA [no, mild, moderate, severe];  Jin YH et al (2020)^136^: yes, multiple choice with binary options;  Khusid et al (2020)^107^: NA, 1-5;  Ko et al (2020)^137^: NA, 1-5;  Lauri Korajlija et al (2020)^55^: NA, 6-30;  Lee SA et al (2020)^56^: NA, 0-4;  Liu S et al (2020)^60^: NA, 1-4;  Lopez et al (2020)^61^: NA, NA;  Roy et al (2020)^73^: ≥4, 1-5;  Sanchez et al (2020)^67^: more, NA [less, no change, more];  Sahu et al (2020)^111^: “definitely stressed out”, NA (happy, not/ only mildly/definitely stressed out)  Shammi et al (2020)^76^: NA, 1-5;  Suleiman et al (2020)^113^: NA, 0-10;  Sutin et al (2020)^147^: NA, 1-5;  Wu W et al (2020)^141^: NA, NA;  Xu H et al (2020)^128^: ≥ „some“, NA [no, some, often, always];  Yassa et al (2020)^129^: yes, ternary item [yes, no, I don’t know] |
| **Dream anxiety score** | Xu J et al (2020)^118^: NA, NA |
| **EPDS** | Durankus et al (2020)^125^: ≥13, 0-30;  Wu Y et al (2020)^127^: ≥10, NA |
| **EPDS-3A** | Wu Y et al (2020)^127^: NA, NA |
| **FCV-19S** | Harper et al (2020)^53^: NA, 7-35;  Reznik et al (2020)^72^: NA, NA;  Satici et al (2020)^75^: NA, 7-35;  Soraci et al (2020)^78^: NA, 7-35;  Tsipropoulou et al (2020)^82^: NA, 7-35 |
| **GAD-2** | González-Sanguino et al (2020)^52^: NA, 0-6;  Ni et al (2020)^140^: ≥3, 0-6;  Zhang WR et al (2020)^144^: ≥3, NA |
| **GAD-7** | Amerio et al (2020)^97^: ≥10, 0-21;  Bacon et al (2020)^44^: NA, 0-28;  Badahdah et al (2020)^98^: ≥10, 0-21;  Bäuerle et al (2020)^45^: ≥5, 0-21;  Cao et al (2020)^48^: NA, NA;  Chang et al (2020)^49^: ≥6, 0-21;  Consolo et al (2020)^103^: ≥10, 0-21;  Gao et al (2020)^50^: ≥10, 0-21;  Guo et al (2020)^131^: ≥5, NA;  Huang Y et al (2020)^134^: ≥11, 0-21;  Iasevoli et al (2020)^135^: ≥11  Kang et al (2020)^106^: NA, NA;  Lai et al (2020)^18^: ≥7, 0-21;  McKay et al (2020)^64^: NA, NA;  Ren et al (2020)^71^: NA, NA;  Rossi et al (2020)^110^: ≥15, NA;  Shevlin et al (2020)^77^: ≥10, NA;  Tsipropoulou et al (2020)^82^: ≥10, 0-21;  Zhang C et al (2020)^120^: ≥5, 0-21;  Zhang J et al (2020)^143^: ≥10, NA;  Zhou SJ et al (2020)^93^: ≥5, 0-21;  Zhu S et al (2020)^145^: ≥5, NA |
| **GPS (posttraumatic stress disorder subscale)** | Rossi et al (2020)^110^: ≥3 of 5 items, NA |
| **HAMA** | Li X et al (2020)^126^: ≥7, NA;  Liu X et al (2020)^174^: NA, NA;  Lu et al (2020)^139^: ≥7, 0-56 |
| **HAMD** | Li X et al (2020)^126^: ≥4, NA;  Liu X et al (2020)^174^: NA, NA;  Lu et al (2020)^139^: ≥7, 0-68 |
| **HADS anxiety subscore** | Özdin et al (2020)^69^: ≥8, 0-21;  Soraci et al (2020)^78^: NA, 0-21;  Yuan R et al (2020)^89^: ≥8, NA |
| **HADS depression subscore** | Özdin et al (2020)^69^: ≥11, 0-21;  Soraci et al (2020)^78^: NA, 0-21;  Yuan R et al (2020)^89^: NA, NA; |
| **HAI** | Özdin et al (2020)^69^: NA, 0-54 |
| **IES** | Odriozola-Gonzalez et al (2020)^66^: ≥26, 0-75;  Zhang Y et al (2020)^92^: ≥26, 0-75 |
| **IES-R** | Chew et al (2020)^102^: ≥24, NA;  Hao F et al (2020)^132^: ≥18, NA;  Kang et al (2020)^106^: NA, NA;  Lai et al (2020)^18^: ≥26, 0-88;  Li Y et al (2020)^58^: ≥24, NA;  Tan B et al (2020)^114^: ≥24, NA;  Tan W et al (2020)^80^: ≥18, NA;  Wang C et al (2020b)^85^: ≥25, NA;  Zhang C et al (2020)^120^: ≥9, 0-88 |
| **ISI** | Amerio et al (2020)^97^: NA, NA;  Hao F et al (2020)^132^: ≥15, 0-28;  Kang et al (2020)^106^: NA, NA;  Lai et al (2020)^18^: ≥14, 0-28;  Ren et al (2020)^71^: NA, NA;  Rossi et al (2020)^110^: ≥22, NA;  Tan W et al (2020)^80^: ≥15, 0-28;  Zhang C et al (2020)^120^: ≥8, 0-28;  Zhang WR et al (2020)^144^: ≥8, NA |
| **K-6** | Hao X et al (2020)^133^: ≥13, 0-24;  Li Y et al (2020)^58^: ≥5, NA;  Shacham et al (2020)^112^: ≥19, 0-30;  Wang H et al (2020)^86^: NA, NA;  Zhang SX et al (2020b)^91^: NA, NA;  Zhang SX et al (2020c)^121^: NA, NA |
| **K-10** | Moccia et al (2020)^65^: ≥25, NA |
| **PCL-C-2** | González-Sanguino et al (2020)^52^: NA, 0-8 |
| **PCL-5** | Guo et al (2020)^131^: NA, NA;  Liu N et al (2020)^59^: ≥2 in 1 criterion-B-item + 1 criterion -C- item + 2 criterion -D-items + 2 criterion -E-items, and, for single items: ≥2;  Ren et al (2020)^71^: NA, NA;  Yin et al (2020)^119^: ≥33, 0-80 |
| **PHQ-2** | Bäuerle et al (2020)^45^: ≥3, range 0-6;  González-Sanguino et al (2020)^52^: NA, 0-6;  Ni et al (2020)^140^: ≥3, 0-6;  Olagoke et al (2020)^146^: NA, NA;  Voitsidis et al (2020)^83^: NA, NA  Zhang WR et al (2020)^144^: ≥3, NA |
| **PHQ-4** | Zhang SX et al (2020c)^121^: NA, NA |
| **PHQ-9** | Amerio et al (2020)^97^: ≥10, 0-27;  Chang et al (2020)^49^: ≥5, 0-27;  Guo et al (2020)^131^: ≥5, NA;  Iasevoli et al (2020)^135^: ≥16, NA;  Kang et al (2020)^106^: NA, NA;  Lai et al (2020)^18^: ≥10, 0-27;  Ren et al (2020)^71^: NA, NA;  Rossi et al (2020)^110^: ≥15, NA;  Sakib et al (2020)^74^: NA, 0-27;  Tsipropoulou et al (2020)^82^: ≥ 10, 0-27;  Zhang C et al (2020)^120^: ≥5, 0-27;  Zhang J et al (2020)^143^: ≥10, NA;  Zhou SJ et al (2020)^93^: ≥5, 0-27;  Zhu S et al (2020)^145^: ≥5, NA |
| **PROMIS-SF anxiety** | Harper et al (2020)^53^: NA, 8-40 |
| **PROMIS-SF depression** | Harper et al (2020)^53^: NA, 7-35 |
| **PSQI** | Huang Y et al (2020)^134^: ≥8, 0-21;  Ma et al (2020)^62^: NA, 0-54;  Liu X et al (2020)^42^: only 4 items, NA;  Xiao et al (2020)^117^: NA, 0-21;  Wang S et al (2020)^115^: ≥8, 0-21;  Wu K et al (2020)^116^: ≥8, 0-21;  Yin et al (2020)^82^: only 4 items, NA;  Yuan S et al (2020)^142^: NA, NA |
| **PSS (-10)** | Badahdah et al (2020)^98^: NA, 0-40;  Germani et al (2020)^51^: ≥14, 10-40;  Guo et al (2020)^131^: NA, NA;  Iasevoli et al (2020)^135^: ≥27, NA;  Liu X et al (2020)^42^: NA, NA  Ren et al (2020)^71^: NA, NA  Rossi et al (2020)^110^: quartile split; no official cut-off for PSS available |
| **PTSD-SS** | Cai X et al (2020)^123^: ≥51, 24-120;  Huang JZ et al (2020)^105^: ≥50, NA |
| **SAS** | Cai X et al (2020)^123^: ≥51, NA;  Huang JZ et al (2020)^105^: ≥49, NA;  Lei et al (2020)^57^: ≥50, NA;  Mo et al (2020)^108^: NA, NA;  Pu et al (2020)^109^: NA, NA;  Wang S et al (2020)^115^: ≥50, NA;  Wang Y et al (2020)^87^: >59, NA;  Wu K et al (2020)^116^: ≥53, NA;  Xiao et al (2020)^117^: NA, NA;  Zhu J et al (2020)^122^: ≥50, NA |
| **SASR** | Xiao et al (2020)^117^: NA, 0-150 |
| **SCL-90** | Cai W et al (2020)^101^: any subscale score ≥3 /positive items ≥ 44 / total score ≥161, NA;  Tian et al (2020)^81^: Global Severity Index≥63, NA;  Wu K et al (2020)^116^: NA, NA |
| **SDS** | Lei et al (2020)^57^: ≥50, NA;  Wang S et al (2020)^115^: ≥50, NA;  Wang Y et al (2020)^87^: ≥63, NA;  Zhu J et al (2020)^122^: ≥50, NA;  Wu K et al (2020)^116^: ≥50, NA;  Yuan B et al (2020)^124^: ≥51, NA;  Cai X et al (2020)^123^: ≥54, NA |
| **SHAI** | Tull et al (2020)^79^: NA, 18-72 |
| **SOS** | Mo et al (2020)^108^: ≥51, 22-110 |
| **SRQ** | Yuan S et al (2020)^142^: NA, NA |
| **SRQ-20** | Zhu S et al (2020)^145^: ≥7, NA |
| **STAI-Y** | Germani et al (2020)^51^: ≥40, 20-80 |
| **TEMPS-A Anxious** | Moccia et al (2020)^65^: NA, NA |
| **TEMPS-A Depressive** | Moccia et al (2020)^65^: NA, NA |
| **VAS** | Gan et al (2020)^104^: NA, 0-100;  Shevlin et al (2020)^77^: NA, 0-100 |
| **VDAS** | Yuan R et al (2020)^89^: NA, NA |
| **WHO-5** | Gao J et al (2020)^50^: <13, 0-25;  Badahdah et al (2020)^98^: < 49, 0-100 |
| **Vicarious Traumatization Questionnaire** | Li Z et al (2020)^138^: NA, NA |

Abbreviations: AIS, Athens Insomnia Scale; BAI, Beck Anxiety Inventory; BDI, Beck Depression Inventory; BDI(-II), Beck Depression Inventory(-II); BIP-Q5, Brief Illness Perception Questionnaire 5; CES-D, Center for Epidemiologic Studies Depression Scale; CoVGAD-7, Generalized Anxiety Disorder Scale-7 for COVID-19 Anxiety; CPDI, CoViD-19 Peritraumatic Distress Index; DASS-21, Depression Anxiety Stress Scale-21; DT, Distress Thermometer; EPDS, Edinburgh Postnatal Depression Scale; EPDS-3A, Edinburgh Postnatal Depression Scale- Anxiety subscale; FCV-19S, Fear of COVID-19 scale; GAD-2(-7), Generalized Anxiety Disorder Scale-2(/-7); GPS-PTSD, Global Psychotrauma Scale-posttraumatic stress disorder subscale; HADS, Hospital Anxiety and Depression Scale; HAI, Health Anxiety Inventory; HAMA, Hamilton Anxiety Rating Scale; HAMD, Hamilton Depression Rating Scale; IES, Impact of Event Scale; IES-R, Impact of Event Scale-Revised; ISI, Insomnia Severity Index; K-6(/-10), Kessler Psychological Distress Scale-6(/-10); MINI, Mini International Neuropsychiatric Interview; NA, not available; NRS, Numeric Rating Scale; PCL-5(-C), Post-traumatic Stress Disorder Checklist-5(/-Civilian Version); PHQ-2(/-4/-9/-15), Patient Health Questionnaire-2(/-4/-9/-15); PROMIS-SFs, Patient Reported Outcomes Measurement Information System short forms; PSQI, Pittsburgh Sleep Quality Index; PSS(-10), Perceived Stress Scale(-10); PTSD-SS, Post-traumatic Stress Disorder Self-rating Scale; SAS, Self-Rating Anxiety Scale; SASR, Stanford Acute Stress Reaction; SCL-90, Symptom Checklist-90; SDS, Self-Rating Depression Scale; SF-12(/-36), Short Form 12 Health Survey; SHAI, Short Health Anxiety Inventory; SOS, Stress Overload Scale; SRQ, Stress Response Questionnaire; SRQ-20, 20-item Self-Report Questionnaire; STAI-Y, State Trait Anxiety Inventory-Y; TEMPS-A, Temperament Evaluation of Memphis, Pisa, Paris and San Diego-Anxious; VAS, Visual Analogue Scale; VDAS, Van Dream Anxiety Scale; WHO-5, World Health Organization- Five Well-Being Index.

**eResults 1. Forest plots of main analyses**

**eFigure 1. Forest plot of main analyses for anxiety, general population**


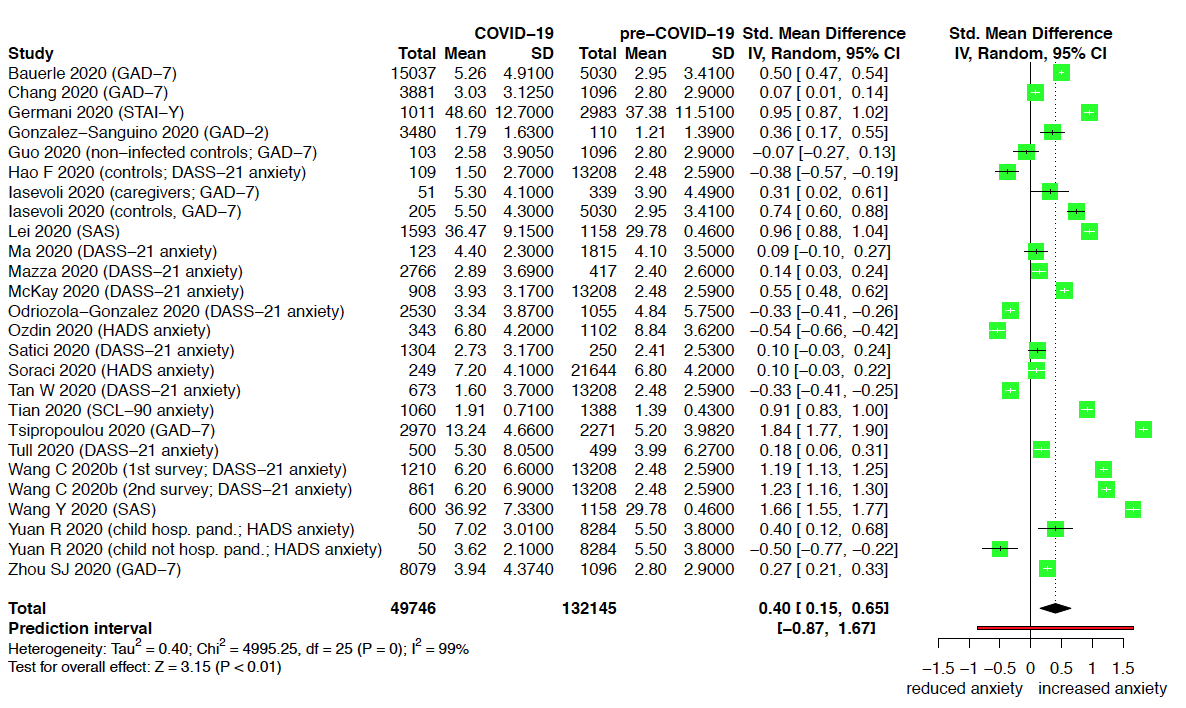


Abbreviations: CI, confidence interval; df, degrees of freedom; I^2^, indicator of statistical heterogeneity; P, p value; SD, standard deviation; Std., standardized; Tau^2^, indicator of statistical heterogeneity; Total, the number of participants; Z, z value; Chi^2^, Chi^2^ test for heterogeneity.

^a^ Horizontal lines indicate the 95% CI of each study; diamond, the pooled estimate with 95% CI; multilevel meta-analysis.

**eFigure 2. Forest plot of main analyses for depression, general population**


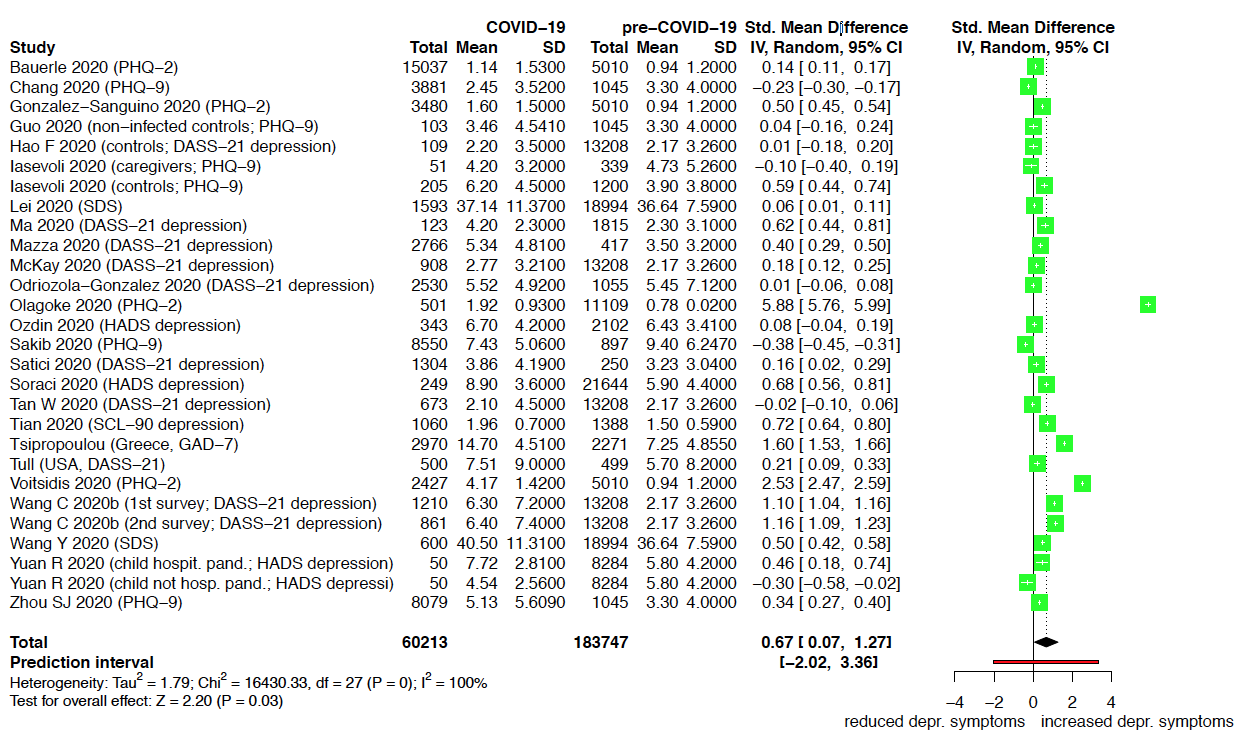


Abbreviations: CI, confidence interval; df, degrees of freedom; I^2^, indicator of statistical heterogeneity; P, p value; SD, standard deviation; Std., standardized; Tau^2^, indicator of statistical heterogeneity; Total, the number of participants; Z, z value; Chi^2^, Chi^2^ test for heterogeneity.

^a^ Horizontal lines indicate the 95% CI of each study; diamond, the pooled estimate with 95% CI; multilevel meta-analysis.

**eFigure 3. Forest plot of main analyses for stress, general population**


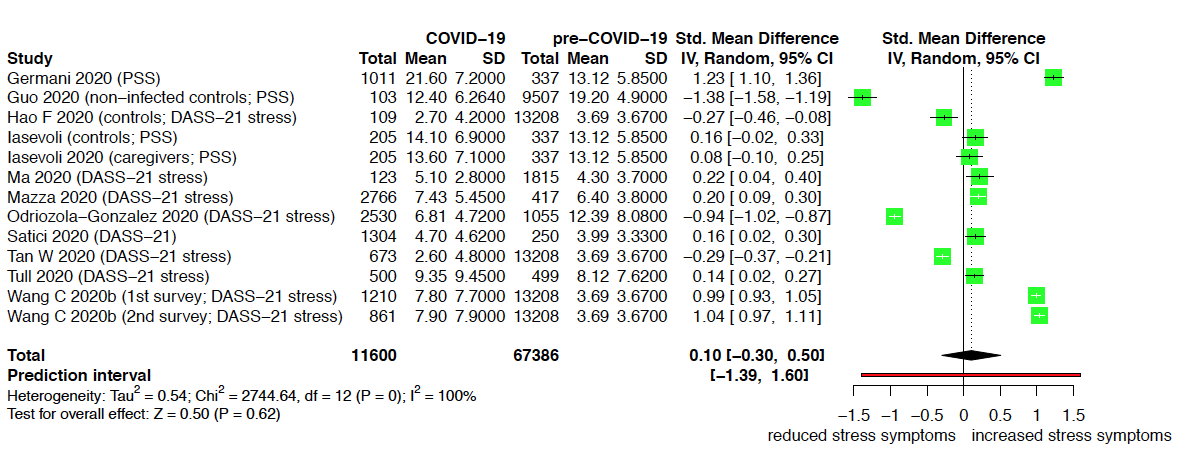


Abbreviations: CI, confidence interval; df, degrees of freedom; I^2^, indicator of statistical heterogeneity; P, p value; SD, standard deviation; Std., standardized; Tau^2^, indicator of statistical heterogeneity; Total, the number of participants; Z, z value; Chi^2^, Chi^2^ test for heterogeneity.

^a^ Horizontal lines indicate the 95% CI of each study; diamond, the pooled estimate with 95% CI; multilevel meta-analysis.

**eFigure 4. Forest plot of main analyses for sleep-related symptoms, general population**


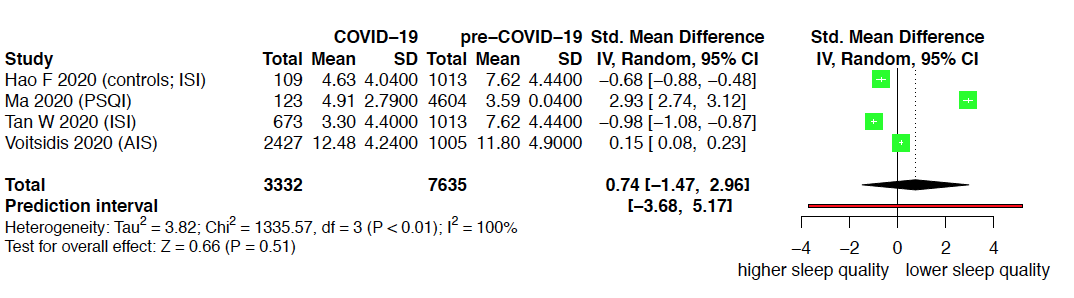


Abbreviations: CI, confidence interval; df, degrees of freedom; I^2^, indicator of statistical heterogeneity; P, p value; SD, standard deviation; Std., standardized; Tau^2^, indicator of statistical heterogeneity; Total, the number of participants; Z, z value; Chi^2^, Chi^2^ test for heterogeneity.

^a^ Horizontal lines indicate the 95% CI of each study; diamond, the pooled estimate with 95% CI; multilevel meta-analysis.

**eFigure 5. Forest plot of main analyses for anxiety, healthcare workers**


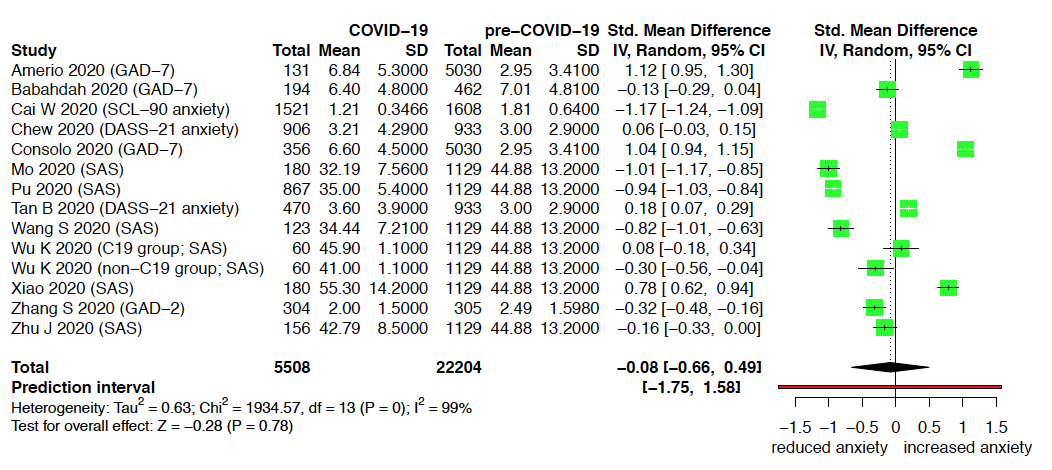


Abbreviations: CI, confidence interval; df, degrees of freedom; I^2^, indicator of statistical heterogeneity; P, p value; SD, standard deviation; Std., standardized; Tau^2^, indicator of statistical heterogeneity; Total, the number of participants; Z, z value; Chi^2^, Chi^2^ test for heterogeneity.

^a^ Horizontal lines indicate the 95% CI of each study; diamond, the pooled estimate with 95% CI; multilevel meta-analysis.

**eFigure 6. Forest plot of main analyses for depression, healthcare workers**


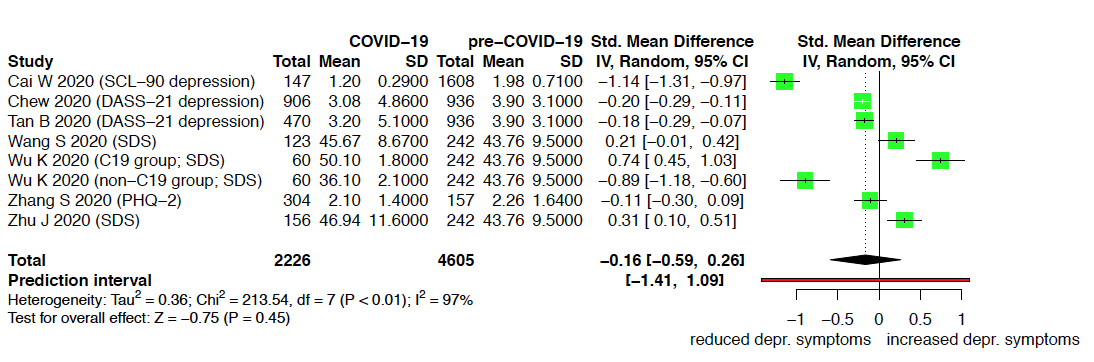


Abbreviations: CI, confidence interval; df, degrees of freedom; I^2^, indicator of statistical heterogeneity; P, p value; SD, standard deviation; Std., standardized; Tau^2^, indicator of statistical heterogeneity; Total, the number of participants; Z, z value; Chi^2^, Chi^2^ test for heterogeneity.

^a^ Horizontal lines indicate the 95% CI of each study; diamond, the pooled estimate with 95% CI; multilevel meta-analysis.

**eFigure 7. Forest plot of main analyses for stress, healthcare workers**


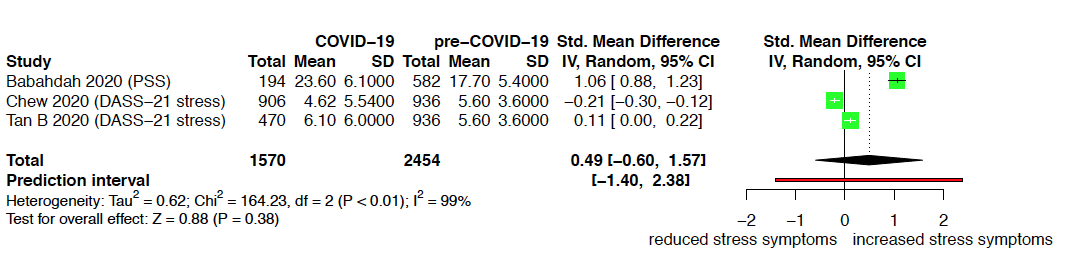


Abbreviations: CI, confidence interval; df, degrees of freedom; I^2^, indicator of statistical heterogeneity; P, p value; SD, standard deviation; Std., standardized; Tau^2^, indicator of statistical heterogeneity; Total, the number of participants; Z, z value; Chi^2^, Chi^2^ test for heterogeneity.

^a^ Horizontal lines indicate the 95% CI of each study; diamond, the pooled estimate with 95% CI; multilevel meta-analysis.

**eFigure 8. Forest plot of main analyses for sleep-related symptoms, healthcare workers**


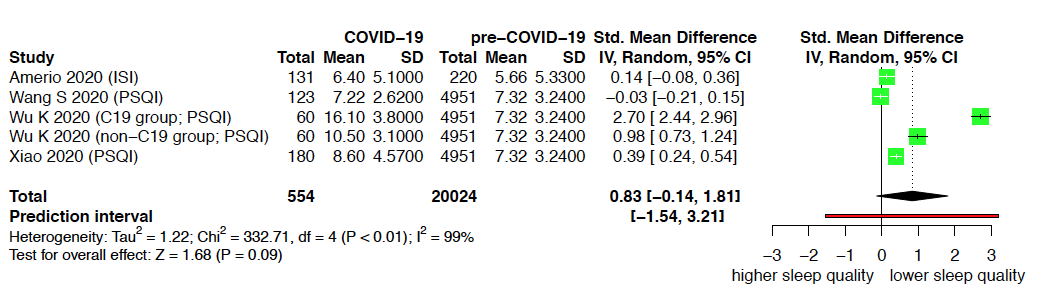


Abbreviations: CI, confidence interval; df, degrees of freedom; I^2^, indicator of statistical heterogeneity; P, p value; SD, standard deviation; Std., standardized; Tau^2^, indicator of statistical heterogeneity; Total, the number of participants; Z, z value; Chi^2^, Chi^2^ test for heterogeneity.

^a^ Horizontal lines indicate the 95% CI of each study; diamond, the pooled estimate with 95% CI; multilevel meta-analysis.

**eFigure 9. Forest plot of main analyses for anxiety, patients**


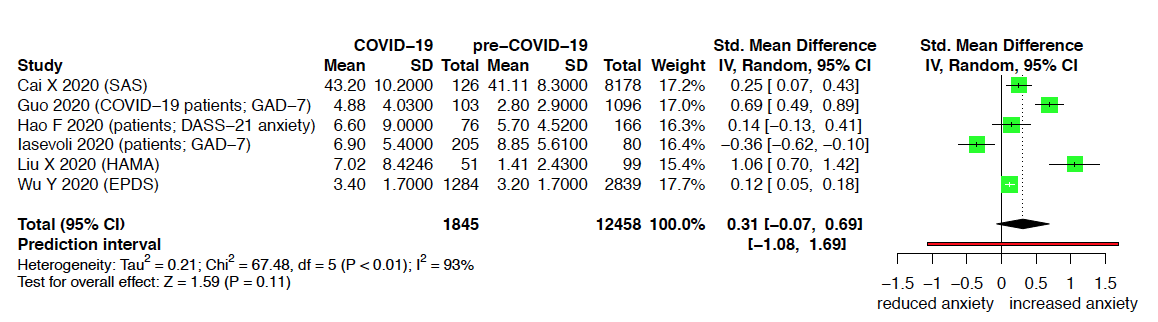


Abbreviations: CI, confidence interval; df, degrees of freedom; I^2^, indicator of statistical heterogeneity; P, p value; SD, standard deviation; Std., standardized; Tau^2^, indicator of statistical heterogeneity; Total, the number of participants; Z, z value; Chi^2^, Chi^2^ test for heterogeneity.

^a^ Horizontal lines indicate the 95% CI of each study; diamond, the pooled estimate with 95% CI; classic random-effects model.

**eFigure 10. Forest plot of main analyses for depression, patients**


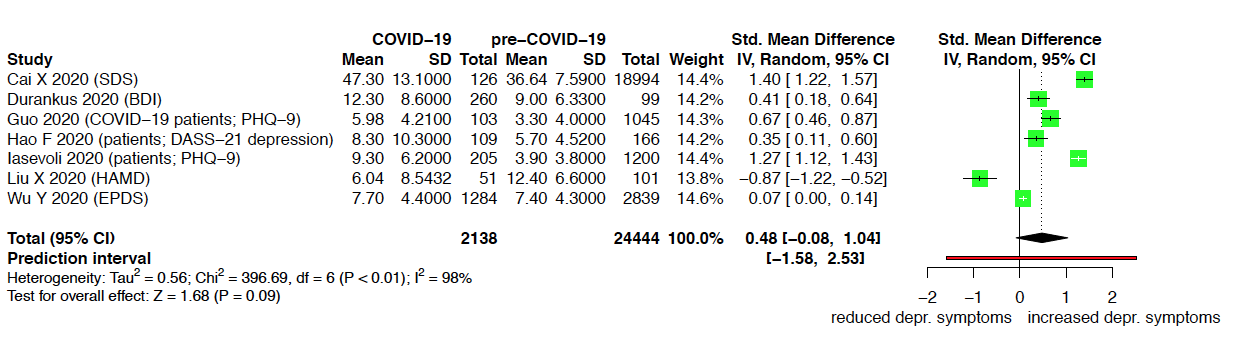


Abbreviations: CI, confidence interval; df, degrees of freedom; I^2^, indicator of statistical heterogeneity; P, p value; SD, standard deviation; Std., standardized; Tau^2^, indicator of statistical heterogeneity; Total, the number of participants; Z, z value; Chi^2^, Chi^2^ test for heterogeneity.

^a^ Horizontal lines indicate the 95% CI of each study; diamond, the pooled estimate with 95% CI; classic random-effects model.

**eFigure 11. Forest plot of main analyses for stress, patients**


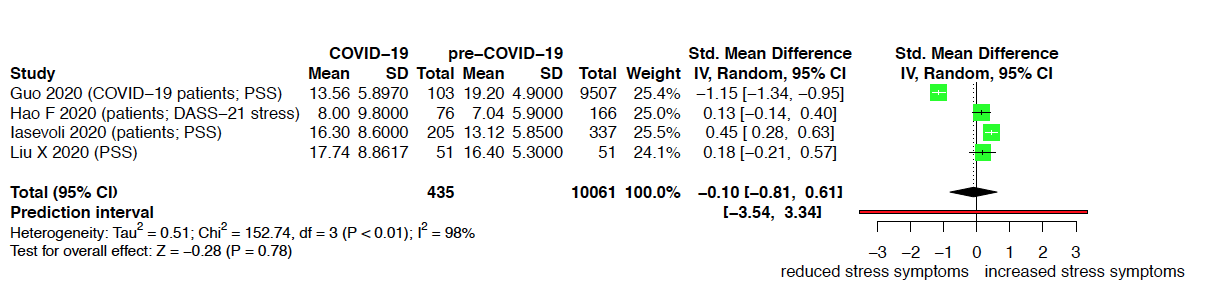


Abbreviations: CI, confidence interval; df, degrees of freedom; I^2^, indicator of statistical heterogeneity; P, p value; SD, standard deviation; Std., standardized; Tau^2^, indicator of statistical heterogeneity; Total, the number of participants; Z, z value; Chi^2^, Chi^2^ test for heterogeneity.

^a^ Horizontal lines indicate the 95% CI of each study; diamond, the pooled estimate with 95% CI; classic random-effects model.

**eFigure 12. Forest plot of main analyses for sleep-related symptoms, patients**


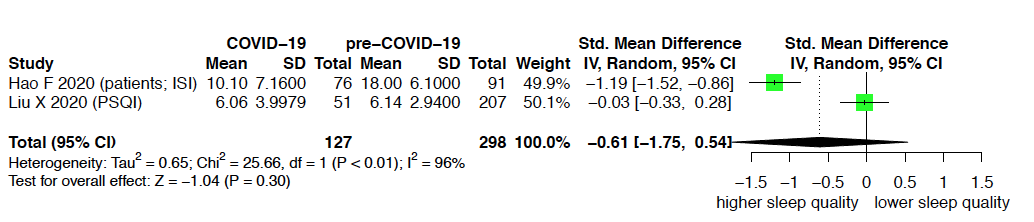


Abbreviations: CI, confidence interval; df, degrees of freedom; I^2^, indicator of statistical heterogeneity; P, p value; SD, standard deviation; Std., standardized; Tau^2^, indicator of statistical heterogeneity; Total, the number of participants; Z, z value; Chi^2^, Chi^2^ test for heterogeneity.

^a^ Horizontal lines indicate the 95% CI of each study; diamond, the pooled estimate with 95% CI; classic random-effects model.

**eTable 12. Quality (risk of bias) assessment of included pandemic studies**

| **Study** | | **1. Research question?** | | **2. Study population clearly defined?** | | **3. Participation rate ≥ 50%?** | | **4. Selection criteria?** | | **5. Sample size justification, power description, variance and effect estimates?** | | | **8. Was the exposure (independent variable) clearly specified?** | | | **9. Was the exposure consistent across all study participants?** | | **11. Outcome measures clearly defined, valid, reliable, and implemented consistently?** | | | **Quality Rating** | | | **Comments** | |
| --- | --- | --- | --- | --- | --- | --- | --- | --- | --- | --- | --- | --- | --- | --- | --- | --- | --- | --- | --- | --- | --- | --- | --- | --- | --- |
| **General population** | | | | | | | | | | | | | | | | | | | | | | | | | |
| Ahmad et al (2020)^43^ | | Y | | N | | NR | | NR | | N | | | | Y | | NR | | N | | | | + | | I, II, III, IV, V | |
| Bacon et al (2020)^44^ | | Y | | Y | | NR | | Y | | N | | | | Y | | Y | | N | | | | + | | IV | |
| Bäuerle et al (2020)^45^; Teufel et al (2020)^46^ | | Y | | Y | | NA | | Y | | N | | | | Y | | N | | Y | | | | ++ | | I | |
| Buzzi et al (2020)^47^ | | Y | | Y | | NR | | Y | | N | | | | Y | | Y | | N | | | | + | | IV | |
| Cao et al (2020)^48^ | | Y | | Y | | Y | | Y | | N | | | | Y | | NR | | Y | | | | ++ | | II, III | |
| Chang et al (2020)^49^ | | Y | | Y | | NA | | Y | | N | | | | Y | | Y | | Y | | | | ++ | | I | |
| Gao J et al (2020)^50^ | | Y | | Y | | NA | | Y | | N | | | | Y | | Y | | Y | | | | ++ | | I | |
| Germani et al (2020)^51^ | | Y | | Y | | NA | | Y | | N | | | | Y | | Y | | Y | | | | ++ | | I | |
| González-Sanguino et al (2020)^52^ | | Y | | Y | | NA | | Y | | N | | | | Y | | Y | | Y | | | | ++ | | I, III | |
| Harper et al (2020)^53^ | | Y | | Y | | NA | | Y | | Y | | | | Y | | Y | | Y | | | | ++ | | I | |
| Jahanshahi et al (2020)^54^ | | Y | | Y | | NA | | N | | N | | | | Y | | Y | | N | | | | + | | I, IV, V | |
| Lauri Korajlija et al (2020)^55^ | | Y | | Y | | NA | | NR | | N | | | | Y | | Y | | N | | | | + | | I, II, IV, V | |
| **Study** | | **1. Research question?** | | **2. Study population clearly defined?** | | **3. Participation rate ≥ 50%?** | | **4. Selection criteria?** | | **5. Sample size justification, power description, variance and effect estimates?** | | | | **8. Was the exposure (independent variable) clearly specified?** | | **9. Was the exposure consistent across all study participants?** | | **11. Outcome measures clearly defined, valid, reliable, and implemented consistently?** | | | | **Quality Rating** | | **Comments** | |
| Lee SA et al (2020)^56^ | | Y | | Y | | NR | | Y | | N | | | | Y | | Y | | N | | | | + | | I, IV, V | |
| Lei et al (2020)^57^ | | Y | | Y | | NA | | Y | | N | | | | Y | | Y | | Y | | | | ++ | | I | |
| Li Y et al (2020)^58^ | | Y | | Y | | Y | | Y | | N | | | | Y | | Y | | Y | | | | +++ | |  | |
| Liu N et al (2020)^59^ | | Y | | N | | NA | | Y | | N | | | | Y | | Y | | Y | | | | + | | I, V | |
| Liu S et al (2020)^60^ | | Y | | Y | | NR | | NR | | N | | | | Y | | NR | | Y | | | | + | | I, II, III | |
| Lopez et al (2020)^61^ | | Y | | N | | NA | | Y | | N | | | | Y | | NR | | N | | | | + | | I, II, IV, V | |
| Ma et al (2020)^62^ | | Y | | Y | | Y | | Y | | N | | | | Y | | NR | | Y | | | | ++ | | II | |
| Mazza et al (2020)^63^ | | Y | | Y | | NA | | Y | | N | | | | Y | | Y | | Y | | | | ++ | | I | |
| McKay et al (2020)^64^ | | Y | | Y | | NA | | NR | | N | | | | Y | | Y | | Y | | | | + | | I, III | |
| Moccia et al (2020)^65^ | | Y | | Y | | NA | | Y | | Y | | | | Y | | Y | | Y | | | | ++ | | I | |
| Odriozola-González et al (2020)^66^ | | Y | | Y | | NA | | Y | | N | | | | Y | | Y | | Y | | | | ++ | | I, III | |
| Olagoke et al (2020)^146^ | | Y | | Y | | NA | | Y | | N | | | | Y | | NR | | Y | | | | + | | I, II | |
| Ozamiz-Etxebarria et al (2020)^68^ | | Y | | Y | | NA | | NR | | N | | | | Y | | Y | | Y | | | | + | | I, III, V | |
| Özdin et al (2020)^69^ | | Y | | Y | | NA | | NR | | Y | | | | Y | | Y | | Y | | | | ++ | | I, V | |
| **Study** | | **1. Research question?** | | **2. Study population clearly defined?** | | **3. Participation rate ≥ 50%?** | | **4. Selection criteria?** | | **5. Sample size justification, power description, variance and effect estimates?** | | | | **8. Was the exposure (independent variable) clearly specified?** | | **9. Was the exposure consistent across all study participants?** | | **11. Outcome measures clearly defined, valid, reliable, and implemented consistently?** | | | | **Quality Rating** | | **Comments** | |
| Perez-Fuentes et al (2020)^70^ | | Y | | Y | | NA | | NR | | N | | | | Y | | Y | | Y | | | | + | | I, III, V | |
| Qiu et al (2020)^41^ | | Y | | Y | | NA | | NR | | N | | | | Y | | Y | | N | | | | + | | I, IV | |
| Ren et al (2020)^71^ | | Y | | N | | NR | | NR | | N | | | | Y | | Y | | Y | | | | + | | I, III, V | |
| Reznik et al (2020)^72^ | | Y | | Y | | NA | | N | | N | | | | Y | | NR | | Y | | | | + | | I, II | |
| Roy et al (2020)^73^ | | Y | | N | | NA | | Y | | N | | | | Y | | Y | | N | | | | + | | I, IV | |
| Sakib et al (2020)^74^ | | Y | | Y | | NA | | Y | | N | | | | Y | | Y | | Y | | | | ++ | | I, III | |
| Satici et al (2020)^75^ | | Y | | Y | | NA | | NR | | N | | | | Y | | NR | | Y | | | | + | | I, II, III, V | |
| Shammi et al (2020)^76^ | | Y | | Y | | NA | | Y | | N | | | | Y | | Y | | N | | | | + | | I, III, IV | |
| Shevlin et al (2020)^77^ | | Y | | Y | | NA | | Y | | N | | | | Y | | Y | | Y | | | | +++ | |  | |
| Soraci et al (2020)^78^ | | Y | | Y | | NA | | Y | | N | | | | Y | | Y | | Y | | | | ++ | | I, iii | |
| Sutin et al (2020)^147^ | | Y | | Y | | NA | | Y | | N | | | | Y | | Y | | N | | | | + | | I, IV | |
| Tan W et al (2020)^80^ | | Y | | Y | | NR | | Y | | N | | | | Y | | Y | | Y | | | | + | | I | |
| Tian et al (2020)^81^ | | Y | | Y | | NA | | Y | | N | | | | Y | | Y | | Y | | | | ++ | | I | |
| Tsipropoulou et al (2020)^82^ | | Y | | Y | | NR | | Y | | Y | | | | Y | | Y | | Y | | | | ++ | | I | |
| Tull et al (2020)^79^ | | Y | | Y | | NA | | Y | | N | | | | Y | | Y | | Y | | | | +++ | | iii | |
| **Study** | | **1. Research question?** | | **2. Study population clearly defined?** | | **3. Participation rate ≥ 50%?** | | **4. Selection criteria?** | | **5. Sample size justification, power description, variance and effect estimates?** | | | | **8. Was the exposure (independent variable) clearly specified?** | | **9. Was the exposure consistent across all study participants?** | | **11. Outcome measures clearly defined, valid, reliable, and implemented consistently?** | | | | **Quality Rating** | | **Comments** | |
| Voitsidis et al (2020)^83^ | | Y | | N | | NR | | N | | N | | | | Y | | Y | | Y | | | | + | | I, III, V | |
| Wang, C et al (2020a)^84^; Wang C et al (2020b)^85^ | | Y | | Y | | NA | | Y | | N | | | | Y | | Y | | Y | | | | ++ | | I | |
| Wang H et al (2020)^86^ | | Y | | Y | | NA | | Y | | N | | | | Y | | Y | | Y | | | | ++ | | I, III | |
| Wang Y et al (2020)^87^ | | Y | | Y | | NA | | Y | | N | | | | Y | | Y | | Y | | | | ++ | | I, V | |
| Yang H et al (2020)^88^ | | Y | | Y | | NA | | NR | | N | | | | Y | | Y | | Y | | | | ++ | | II, III | |
| Yuan R et al (2020)^89^ | | Y | | Y | | NR | | NR | | N | | | | Y | | Y | | Y | | | | + | | I, II, III, V | |
| ^a^ Zhang SX et al (2020a)^90^; Zhang SX et al (2020b)^91^ | | Y | | Y | | NR | | Y | | N | | | | Y | | Y | | Y | | | | ++ | | I, iii | |
| Zhang Y et al (2020)^92^ | | Y | | Y | | Y | | Y | | N | | | | Y | | Y | | Y | | | | ++ | | I | |
| Zhou SJ et al (2020)^93^ | | Y | | Y | | NR | | Y | | N | | | | Y | | Y | | Y | | | | + | | I | |
| **Healthcare workers** | | | | | | | | | | | | | | | | | | | | | | | | | |
| Abdessater et al (2020)^94^ | | Y | | Y | | Y | | Y | | N | | | | Y | | Y | | N | | | | + | | I, IV | |
| Ahmed et al (2020)^95^ | | Y | | Y | | NA | | Y | | N | | | | Y | | Y | | N | | | | + | | I, IV | |
| Alhaj et al (2020)^96^ | | Y | | N | | N | | NR | | N | | | | Y | | Y | | N | | | | + | | I, IV | |
| Amerio et al (2020)^97^ | | Y | | Y | | N | | Y | | N | | | | Y | | N | | Y | | | | + | | I | |
| **Study** | **1. Research question?** | | **2. Study population clearly defined?** | | **3. Participation rate ≥ 50%?** | | **4. Selection criteria?** | | **5. Sample size justification, power description, variance and effect estimates?** | | | **8. Was the exposure (independent variable) clearly specified?** | | | **9. Was the exposure consistent across all study participants?** | | **11. Outcome measures clearly defined, valid, reliable, and implemented consistently?** | | | **Quality Rating** | | | **Comments** | |  |
| Badahdah et al (2020)^98^ | Y | | N | | NR | | N | | N | | | Y | | | NR | | Y | | | + | | | I, II, iii, V | |  |
| Bohlken et al (2020)^99^ | Y | | Y | | N | | Y | | N | | | Y | | | Y | | N | | | + | | | IV, VII | |  |
| Cai H et al (2020)^100^ | Y | | Y | | NR | | NR | | N | | | Y | | | NR | | N | | | + | | | I, II, IV | |  |
| Cai W et al (2020)^101^ | Y | | Y | | NR | | NR | | N | | | Y | | | NR | | Y | | | + | | | I, II, V | |  |
| Chew et al (2020)^102^ | Y | | Y | | NA | | Y | | N | | | Y | | | N | | Y | | | ++ | | | I, V | |  |
| Consolo et al (2020)^103^ | Y | | N | | NA | | NR | | Y | | | Y | | | Y | | Y | | | + | | | I, V | |  |
| Gan et al (2020)^104^ | Y | | Y | | N | | Y | | Y | | | Y | | | Y | | Y | | | ++ | | | I | |  |
| Huang JZ et al (2020)^105^ | Y | | Y | | Y | | Y | | N | | | Y | | | Y | | Y | | | +++ | | |  | |  |
| Kang et al (2020)^106^ | Y | | Y | | NR | | NR | | N | | | Y | | | Y | | Y | | | + | | | I, VI | |  |
| Khusid et al (2020)^107^ | Y | | Y | | N | | Y | | N | | | Y | | | Y | | N | | | + | | | I, III, IV, VII | |  |
| Lai et al (2020)^18^ | Y | | Y | | Y | | Y | | Y | | | Y | | | Y | | Y | | | +++ | | |  | |  |
| Mo et al (2020)^108^ | Y | | Y | | Y | | NR | | N | | | Y | | | NR | | Y | | | + | | | I, II | |  |
| Pu et al (2020)^109^ | Y | | Y | | NR | | NR | | N | | | Y | | | NR | | Y | | | + | | | II, iii | |  |
| Rossi et al (2020)^110^ | Y | | Y | | NA | | Y | | N | | | Y | | | Y | | Y | | | ++ | | | I | |  |
| Sahu et al (2020)^111^ | Y | | N | | NA | | Y | | N | | | Y | | | Y | | N | | | + | | | I, III, IV, V | |  |
| **Study** | **1. Research question?** | | **2. Study population clearly defined?** | | **3. Participation rate ≥ 50%?** | | **4. Selection criteria?** | | **5. Sample size justification, power description, variance and effect estimates?** | | | **8. Was the exposure (independent variable) clearly specified?** | | | **9. Was the exposure consistent across all study participants?** | | **11. Outcome measures clearly defined, valid, reliable, and implemented consistently?** | | | **Quality Rating** | | | **Comments** | |  |
| Shacham et al (2020)^112^ | Y | | Y | | NA | | Y | | N | | | Y | | | Y | | Y | | | ++ | | | I | |  |
| Suleiman et al (2020)^113^ | Y | | Y | | Y | | Y | | N | | | Y | | | Y | | N | | | + | | | I, IV | |  |
| Tan B et al (2020)^114^ | Y | | Y | | Y | | Y | | N | | | Y | | | Y | | Y | | | +++ | | | iii | |  |
| Wang S et al (2020)^115^ | Y | | Y | | Y | | Y | | N | | | Y | | | Y | | Y | | | +++ | | |  | |  |
| Wu K et al (2020)^116^ | Y | | Y | | NR | | Y | | N | | | Y | | | NR | | Y | | | + | | | I, II | |  |
| Xiao et al (2020)^117^ | Y | | Y | | Y | | NR | | N | | | Y | | | NR | | Y | | | + | | | II, III | |  |
| Xu J et al (2020)^118^ | Y | | Y | | NR | | NR | | N | | | Y | | | Y | | N | | | + | | | I, IV, V | |  |
| Yin et al (2020)^119^ | Y | | Y | | NA | | Y | | N | | | Y | | | Y | | Y | | | ++ | | | I | |  |
| Zhang C et al (2020)^120^ | Y | | Y | | NA | | Y | | N | | | Y | | | Y | | Y | | | ++ | | | I | |  |
| Zhang SX et al (2020c)^121^ | Y | | Y | | NR | | NR | | N | | | Y | | | Y | | Y | | | + | | | I, iii | |  |
| Zhu J et al (2020)^122^ | Y | | Y | | NA | | Y | | N | | | Y | | | Y | | Y | | | ++ | | | I | |  |
| **Patients** | | | | | | | | | | | | | | | | | | | | | | | | |  |
| ^b^Cai X et al (2020)^123^; Yuan B et al (2020)^124^ | Y | | Y | | Y | | Y | | N | | | Y | | | Y | | Y | | | +++ | | |  | |  |
| Durankus et al (2020)^125^ | Y | | N | | Y | | Y | | N | | | Y | | | NR | | Y | | | + | | | I, II, V | |  |
| Li X et al (2020)^126^ | Y | | Y | | Y | | Y | | N | | | Y | | | Y | | Y | | | +++ | | |  | |  |
| **Study** | **1. Research question?** | | **2. Study population clearly defined?** | | **3. Participation rate ≥ 50%?** | | **4. Selection criteria?** | | **5. Sample size justification, power description, variance and effect estimates?** | | | **8. Was the exposure (independent variable) clearly specified?** | | | **9. Was the exposure consistent across all study participants?** | | **11. Outcome measures clearly defined, valid, reliable, and implemented consistently?** | | | **Quality Rating** | | | **Comments** | |  |
| Liu X et al (2020a)^42^ | Y | | Y | | Y^c^ | | Y | | N | | | Y | | | Y | | Y | | | +++, ++^d^ | | | I^e^ | |  |
| Wu Y et al (2020)^127^ | Y | | Y | | NA | | Y | | N | | | Y | | | N | | Y | | | ++ | | |  | |  |
| Xu H et al (2020)^128^ | Y | | Y | | NR | | Y | | N | | | Y | | | Y | | N | | | + | | | I, IV | |  |
| Yassa et al (2020)^129^ | Y | | Y | | NR | | Y | | N | | | Y | | | NR | | N | | | + | | | I, II, IV | |  |
| **Mixed groups** | | | | | | | | | | | | | | | | | | | | | | | | |  |
| Büntzel et al (2020)^130^ | Y | | Y | | NA | | NR | | N | | Y | | | | Y | | N | | + | | | | I, IV, V | |  |
| Guo et al (2020)^131^ | Y | | Y | | NR | | Y | | N | | Y | | | | Y | | Y | | + | | | | I | |  |
| Hao F et al (2020)^132^ | Y | | Y | | NA, NR^f^ | | Y | | N | | Y | | | | Y | | Y | | ++, +^g^ | | | | I | |  |
| Hao X et al (2020)^133^ | Y | | Y | | NA | | Y | | N | | Y | | | | Y | | Y | | ++ | | | | I | |  |
| Huang Y et al (2020)^134^ | Y | | Y | | NA | | Y | | N | | Y | | | | Y | | Y | | ++ | | | | I | |  |
| Iasevoli et al (2020)^135^ | Y | | Y | | NR | | Y | | N | | Y | | | | Y | | Y | | + | | | | I | |  |
| Jin YH et al (2020)^136^ | Y | | Y | | Y | | Y | | N | | Y | | | | Y | | N | | ++ | | | | IV | |  |
| Ko et al (2020)^137^ | Y | | Y | | NA | | Y | | N | | Y | | | | Y | | N | | + | | | | I, IV | |  |
| Li Z et al (2020)^138^ | Y | | N | | NA | | Y | | N | | Y | | | | Y | | N | | + | | | | III, V | |  |
| Lu W et al (2020)^139^ | Y | | Y | | Y | | Y | | N | | Y | | | | Y | | Y | | ++, +++^h^ | | | | I^i^ | |  |
| Ni et al (2020)^140^ | Y | | Y | | NA | | Y | | N | | Y | | | | Y | | Y | | ++ | | | | I | |  |
| **Study** | **1. Research question?** | | **2. Study population clearly defined?** | | **3. Participation rate ≥ 50%?** | | **4. Selection criteria?** | | **5. Sample size justification, power description, variance and effect estimates?** | | **8. Was the exposure (independent variable) clearly specified?** | | | | **9. Was the exposure consistent across all study participants?** | | **11. Outcome measures clearly defined, valid, reliable, and implemented consistently?** | | **Quality Rating** | | | | **Comments** | |  |
| Sanchez et al (2020)^67^ | Y | | Y | | NA | | Y | | N | | Y | | | | Y | | N | | + | | | | I, IV | |  |
| Wu W et al (2020)^141^ | Y | | Y | | NR | | Y | | N | | Y | | | | Y | | N | | + | | | | IV | |  |
| Yuan S et al (2020)^142^ | Y | | N | | NR | | NR | | N | | Y | | | | Y | | N | | + | | | | II, III, V | |  |
| Zhang J et al (2020)^143^ | Y | | N | | NR | | NR | | N | | Y | | | | Y | | Y | | + | | | | I, V | |  |
| Zhang WR et al (2020)^144^ | Y | | Y | | NA | | Y | | N | | Y | | | | Y | | Y | | ++ | | | | I, III | |  |
| Zhu S et al (2020)^145^ | Y | | Y | | NA | | NR | | N | | Y | | | | Y | | Y | | + | | | | I, V | |  |

Abbreviations: +, poor; ++, fair; +++, high; Y, Yes; N, No; NA, not applicable; NR, not reported; C19, COVID-19; Comments: I, (possible) selection bias because of insufficient information; II, no or insufficient details on survey period; III, no cut-off-values and/or scale range for the outcome assessment reported (iii, no cut-off); IV, no validated assessment measure for the outcome or outcome measure not clearly defined; V, insufficient description of the study sample; VI, insufficient justification of the summary of the outcomes; VII, reporting bias;

^a^ data description by Zhang XS et al (2020b)^91^ considered.

^b^ data description by Cai X et al (2020)^123^ considered.

^c^ Liu X et al (2020a)^42^: sample 1 (C19 suspected): Y; sample 2 (not C19 suspected): NA.

^d^ Liu X et al (2020a)^42^: sample 1 (C19 suspected): +++; sample 2 (not C19 suspected): ++.

^e^ sample 2 (not C19 suspected): comment a.

^f^ Hao F et al (2020)^132^: sample 1 (control sample): NA; sample 2 (patients): NR.

^g^ Hao F et al (2020)^132^: sample 1 (control sample): ++; sample 2 (patients): +.

^h^ Lu W et al (2020)^139^: sample 1 (general population): ++; sample 2 (healthcare workers): +++.

^i^ Lu W et al (2020)^139^: a for sample 1; for studies including samples with two different overall quality ratings, a more conservative rating was used for the quantitative analysis described in the main text (eg, 57 studies judged at poor quality).

**eTable 13. Assessment of level of comparability between pandemic and prepandemic comparative studies**

| Comparative study | **Pandemic study to compare with** | **Level of comparability^a^** | **Comments: pandemic study vs comparative study** |
| --- | --- | --- | --- |
| Alosaimi et al (2018)^148^ | Badahdah et al (2020)^98^ | **+** | HCW (Oman) vs students (Lebanon) |
| Balestrieri et al (2010)^149^ | Soraci et al (2020)^78^ | **++** | GP (Italy) vs primary care sample (Italy) |
| Basta et al (2019)^150^ | Tsipropoulou et al (2020)^82^ | **++** | GP (Greece) vs young GP (Greece) |
| Bilgel et al (2010)^151^ | Özdin et al (2020)^69^ | **++** | GP (Turkey) vs students (Turkey) |
| Bonfiglio et al (2016)^152^ | Germani et al (2020)^51^ | **+++** |  |
|  | Iasevoli et al (2020, control group)^135^ | **+++** |  |
|  | Iasevoli et al (2020, caregiver group)^135^ | **+++** |  |
|  | Iasevoli et al (2020, patient group)^135^ | **++** | psychiatric patients (Italy) vs COPD patients (Italy) |
| Bottesi et al (2015)^153^ | Mazza et al (2020)^63^ | **+++** |  |
| Cai S et al (2018)^154^ | Cai W et al (2020)^101^ | **+++** |  |
| Carlucci et al (2018)^155^ | Germani et al (2010)^51^ | **+++** |  |
| Carta et al (2013)^156^ | Iasevoli et al (2020, control group)^51^ | **+++** |  |
|  | Iasevoli et al (2020, patient group)^51^ | **++** | P (Italy) vs GP (Italy) |
| Choueiry et al (2016)^157^ | Babahdah et al (2020)^98^ | **+** | HCW (Oman) vs students (Lebanon) |
| Chung et al (2010)^158^ | Hao F et al (2020)^132^ | **+++** |  |
| Dadfar et al (2019)^159^ | Zhang SX et al (2020c)^121^ | **++** | HCW (Iran) vs GP (Iran) |
| Dong et al (2017)^160^ | Xiao et al (2020)^117^ | **++++** |  |
|  | Wang S (2020)^115^ | **++++** |  |
|  | Wu K et al (2020, C19-group)^116^ | **++++** |  |
|  | Wu K et al (2020, nC19-group)^116^ | **++++** |  |
| Ertektin et al (2018)^161^ | Durankus et al (2020)^125^ | **++++** |  |
| Fleishman et al (2007)^162^ | Olagoke et al (2020)^146^ | **++++** |  |
| García-Campayo et al (2012)^163^ | González-Sanguino et al (2020)^52^ | **++** | GP (Spain) vs primary care sample (Spain) |
| Ho R et al (2016)^164^ | Liu X et al (2020a)^42^ | **+++** |  |
| Hossain et al (2019)^165^ | Sakib et al (2020)^74^ | **++** | GP (Bangladesh) vs students (Bangladesh) |
| Huang et al (2020)^166^ | Guo et al (2020)^131^ | **+++** |  |
| Ivziku et al (2019)^167^ | Iasevoli et al (2020, patient group)^135^ | **+++** |  |
| Jeyagurunathan et al (2017)^168^ | Iasevoli et al (2020, caregiver group)^135^ | **+** | caregivers (Italy) vs caregivers (Singapore) |
| Jin et al (1986)^169^ | Tian et al (2020)^81^ | **+++** |  |
| Lee K et al (2017)^170^ | Yuan R et al (2020, group EH)^89^ | **++++** |  |
|  | Yuan R et al (2020, group NEH)^89^ | **++++** |  |
| Lin R et al (2018)^171^ | Hao F et al (2020, controls)^132^ | **++** | GP (China) vs students (China) |
|  | Tan W et al (2020)^80^ | **++** | GP (China) vs students (China) |
| Liu H et al (2009)^172^ | Liu X et al (2020a)^42^ | **+++** |  |
| Liu R et al (2016)^173^ | Ma et al (2020)^62^ | **+++** |  |
| Liu X et al (2020)^174^ | Wang S et al (2020)^115^ | **++++** |  |
|  | Zhu J et al (2020)^122^ | **++++** |  |
|  | Wu K et al (2020, C19 group)^116^ | **++++** |  |
|  | Wu K et al (2020, nC19 group)^116^ | **++++** |  |
| Löwe et al (2008)^175^ | Amerio et al (2020)^97^ | **+** | HCW (Italy) vs GP (Germany) |
|  | Bäuerle et al (2020)^45^ | **++++** |  |
|  | Consolo et al (2020)^103^ | **+** | HCW (Italy) vs GP (Germany) |
|  | Iasevoli et al (2020, control group)^135^ | **+** | GP (Italy) vs GP (Germany) |
| Löwe et al (2010)^176^ | Bäuerle et al (2020)^45^ | **++++** |  |
|  | González-Sanguino et al (2020)^52^ | **+** | GP (Spain) vs GP (Germany) |
|  | Voitsidis et al (2020)^83^ | **+** | GP (Greece) vs GP (Germany) |
| Lu S et al (2018)^177^ | Hao F et al (2020; controls)^132^ | **++** | GP (China) vs students (China) |
|  | McKay et al (2020)^64^ | **++** | GP (China) vs students (China) |
|  | Tan W et al (2020)^80^ | **++** | GP (China) vs students (China) |
|  | Wang C et al (2020b, 1st survey)^85^ | **++** | GP (China) vs students (China) |
|  | Wang C et al (2020b, 2nd survey)^85^ | **++** | GP (China) vs students (China) |
| Lu W et al (2017)^178^ | Guo et al (2020, controls)^131^ | **++** | GP vs. students |
|  | Guo et al (2020, patients)^131^ | **++** | P vs GP |
|  | Chang et al (2020)^49^ | **+++** |  |
|  | Zhou SJ et al (2020)^93^ | **+++** |  |
| Paparrigopoulos et al (2010)^179^ | Voitsidis et al (2020)^83^ | **++++** |  |
| Pereira-Lima et al (2014)^180^ | Zhang SX et al (2020c)^121^ | **+** | HCW (Iran) vs HCW (Brazil) |
| Ramón-Arbués et al (2019)^181^ | Odriozola-González et al (2020)^66^ | **+++** |  |
| Sasaki et al (2020)^182^ | Chew et al (2020)^102^ | **+** | HCW (Singapore, India) vs HCW (Vietnam) |
|  | Tan B et al (2020)^114^ | **+** | HCW (Singapore) vs HCW (Vietnam) |
| Schmidt et al (2015)^183^ | Amerio et al (2020)^97^ | **+** | HCW (Italy) vs HCW (Switzerland) |
| Sinclair et al (2012)^184^ | Tull et al (2020)^79^ | **++++** |  |
| Wang K et al (2016)^185^ | Hao F et al (2020; patient group)^132^ | **+++** |  |
|  | Ma et al (2020)^62^ | **++** | GP (China) vs students (China) |
| Wang W et al (2014)^186^ | Chang et al (2020)^49^ | **++++** |  |
|  | Guo et al (2020, controls)^131^ | **++++** |  |
|  | Guo et al (2020, patients)^131^ | **++** | P (China) vs GP (China) |
|  | Zhou SJ et al (2020)^93^ | **++++** |  |
| Wang WL et al (2020)^187^ | Liu X et al (2020a)^42^ | **+++** |  |
| Wang X et al (1999)^188^ | Lei et al (2020)^57^ | **+++** |  |
|  | Wang Y et al (2020)^87^ | **+++** |  |
| Wang Y et al (2019)^189^ | Liu X et al (2020a)^42^ | **+++** |  |
| Wu Y et al (2020)^127^ | Wu Y et al (2020)^127^ | **++++** |  |
| Yang X et al (2016)^190^ | Cai X et al (2020)^123^ | **++** | P (China) vs students (China) |
| Yildirim et al (2018)^191^ | Satici et al (2020)^75^ | **+++** |  |
| Yu B et al (2019)^192^ | Cai X et al (2020)^123^ | **++** | P (China) vs GP (China) |
|  | Lei et al (2020)^57^ | **+++** |  |
|  | Wang Y et al (2020)^87^ | **+++** |  |
| Zhou et al (2020)^193^ | Mo et al (2020)^108^ | **++++** |  |
|  | Pu et al (2020)^109^ | **++++** |  |
|  | Wang S et al (2020)^115^ | **++++** |  |
|  | Wu K et al (2020)^116^ | **++++** |  |
|  | Wu K et al (2020)^116^ | **++++** |  |
|  | Xiao et al (2020)^117^ | **++++** |  |
|  | Zhu J et al (2020)^122^ | **++++** |  |

Abbreviations: GP, general population; HCW, healthcare workers; P, patients; vs, versus.

^a^ Level of comparability (see eTable 8): +, level 1; ++, level 2; +++, level 3; ++++, level 4.

**eResults 2. Forest plots of sensitivity analyses**

**eFigure 13. Forest plot for sensitivity analysis (quality of pandemic studies) for anxiety, general population**


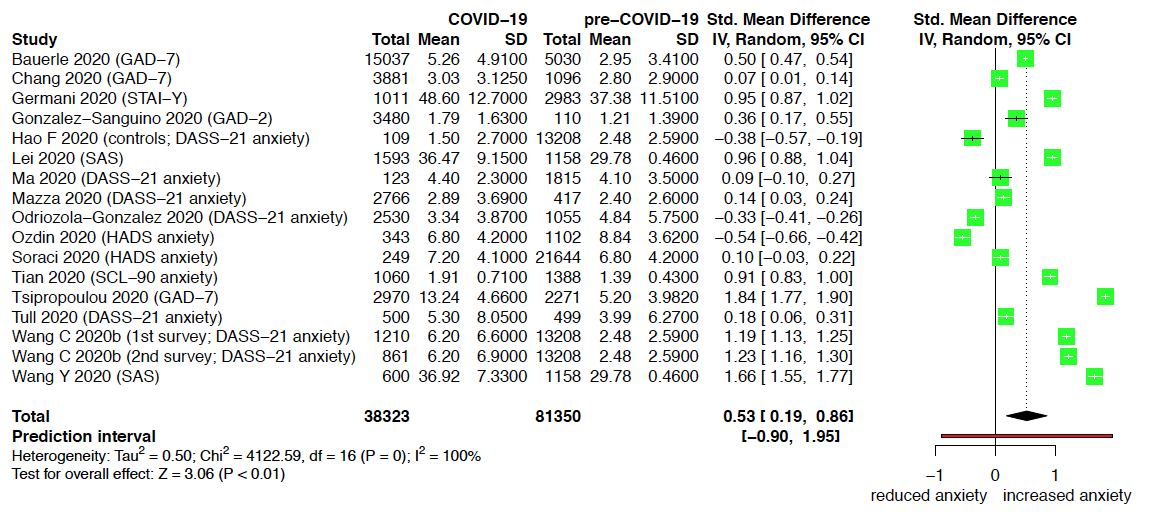


Abbreviations: CI, confidence interval; df, degrees of freedom; I^2^, indicator of statistical heterogeneity; P, p value; SD, standard deviation; Std., standardized; Tau^2^, indicator of statistical heterogeneity; Total, the number of participants; Z, z value; Chi^2^, Chi^2^ test for heterogeneity.

^a^ Horizontal lines indicate the 95% CI of each study; diamond, the pooled estimate with 95% CI; multilevel meta-analysis.

**eFigure 14. Forest plot for sensitivity analysis (quality of pandemic studies) for anxiety, healthcare workers**


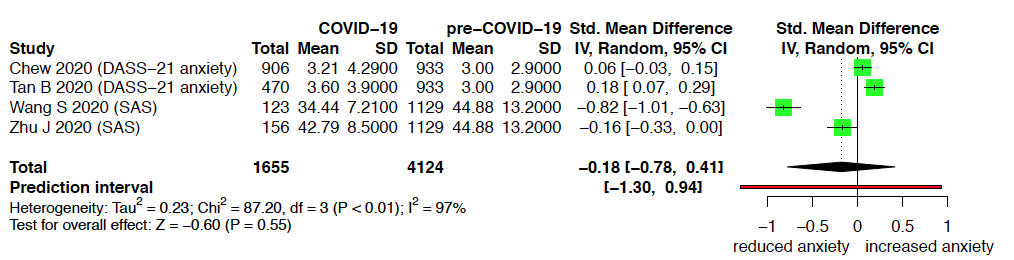


Abbreviations: CI, confidence interval; df, degrees of freedom; I^2^, indicator of statistical heterogeneity; P, p value; SD, standard deviation; Std., standardized; Tau^2^, indicator of statistical heterogeneity; Total, the number of participants; Z, z value; Chi^2^, Chi^2^ test for heterogeneity.

^a^ Horizontal lines indicate the 95% CI of each study; diamond, the pooled estimate with 95% CI; multilevel meta-analysis.

**eFigure 15. Forest plot for sensitivity analysis (quality of pandemic studies) for anxiety, patients**


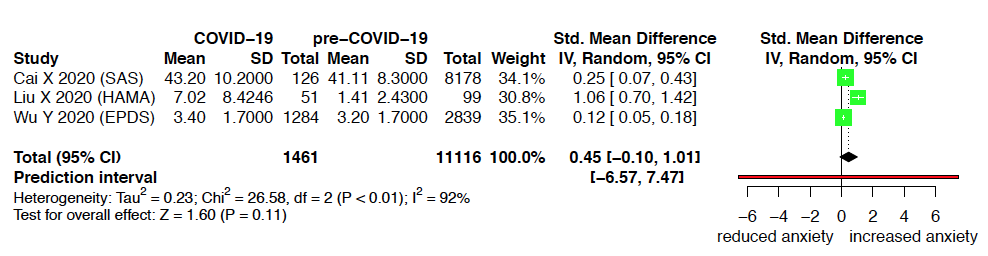


Abbreviations: CI, confidence interval; df, degrees of freedom; I^2^, indicator of statistical heterogeneity; P, p value; SD, standard deviation; Std., standardized; Tau^2^, indicator of statistical heterogeneity; Total, the number of participants; Z, z value; Chi^2^, Chi^2^ test for heterogeneity.

^a^ Horizontal lines indicate the 95% CI of each study; diamond, the pooled estimate with 95% CI; classic random-effects model.

**eFigure 16. Forest plot for sensitivity analysis (quality of pandemic studies) for depression, general population**


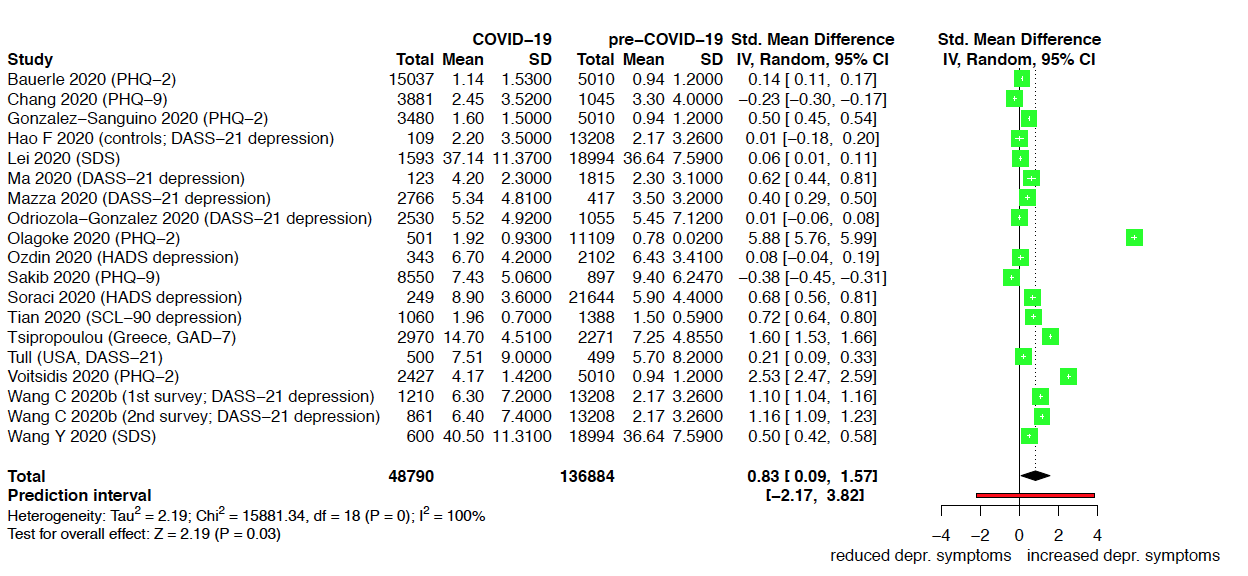


Abbreviations: CI, confidence interval; df, degrees of freedom; I^2^, indicator of statistical heterogeneity; P, p value; SD, standard deviation; Std., standardized; Tau^2^, indicator of statistical heterogeneity; Total, the number of participants; Z, z value; Chi^2^, Chi^2^ test for heterogeneity.

^a^ Horizontal lines indicate the 95% CI of each study; diamond, the pooled estimate with 95% CI; multilevel meta-analysis.

**eFigure17. Forest plot for sensitivity analysis (quality of pandemic studies) for depression, healthcare workers**


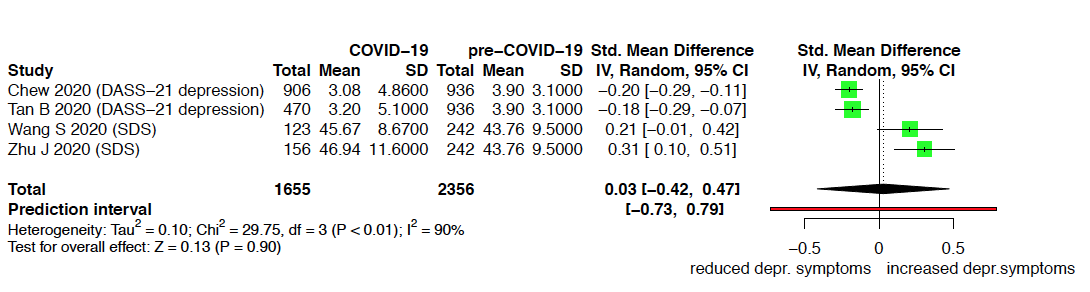


Abbreviations: CI, confidence interval; df, degrees of freedom; I^2^, indicator of statistical heterogeneity; P, p value; SD, standard deviation; Std., standardized; Tau^2^, indicator of statistical heterogeneity; Total, the number of participants; Z, z value; Chi^2^, Chi^2^ test for heterogeneity.

^a^ Horizontal lines indicate the 95% CI of each study; diamond, the pooled estimate with 95% CI; multilevel meta-analysis.

**eFigure 18. Forest plot for sensitivity analysis (quality of pandemic studies) for depression, patients**


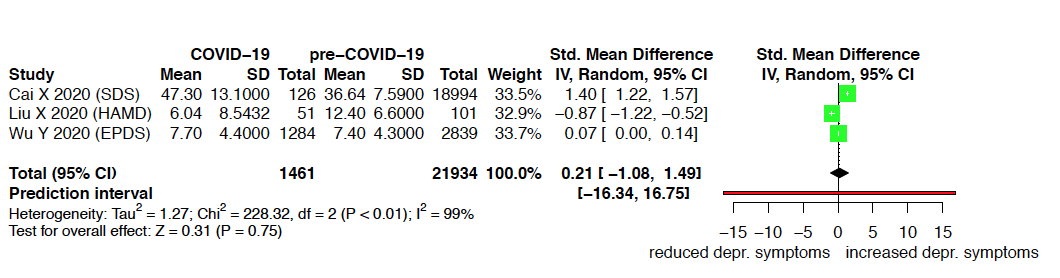


Abbreviations: CI, confidence interval; df, degrees of freedom; I^2^, indicator of statistical heterogeneity; P, p value; SD, standard deviation; Std., standardized; Tau^2^, indicator of statistical heterogeneity; Total, the number of participants; Z, z value; Chi^2^, Chi^2^ test for heterogeneity.

^a^ Horizontal lines indicate the 95% CI of each study; diamond, the pooled estimate with 95% CI; classic random-effects model.

**eFigure 19. Forest plot for sensitivity analysis (quality of pandemic studies) for stress, general population**


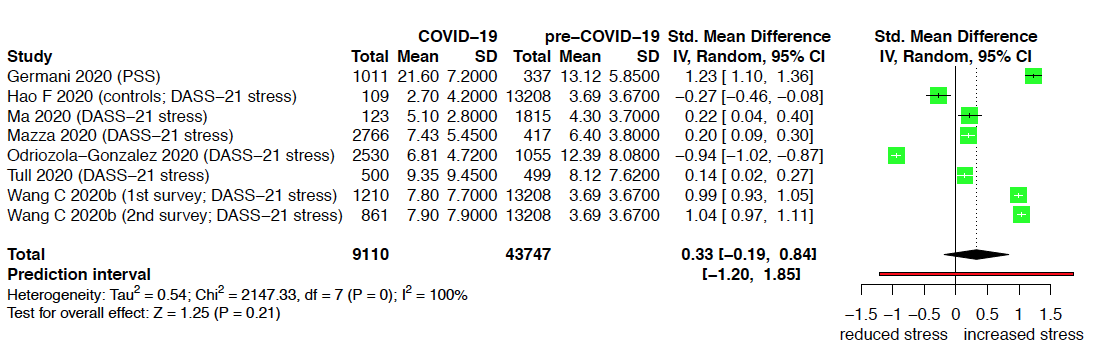


Abbreviations: CI, confidence interval; df, degrees of freedom; I^2^, indicator of statistical heterogeneity; P, p value; SD, standard deviation; Std., standardized; Tau^2^, indicator of statistical heterogeneity; Total, the number of participants; Z, z value; Chi^2^, Chi^2^ test for heterogeneity.

^a^ Horizontal lines indicate the 95% CI of each study; diamond, the pooled estimate with 95% CI; multilevel meta-analysis.

**eFigure 20. Forest plot for sensitivity analysis (quality of pandemic studies) for stress, healthcare workers**


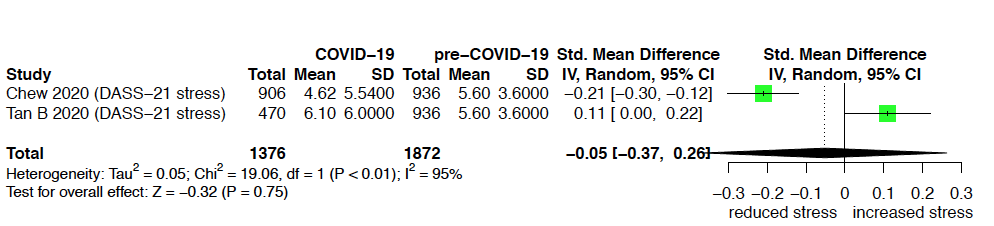


Abbreviations: CI, confidence interval; df, degrees of freedom; I^2^, indicator of statistical heterogeneity; P, p value; SD, standard deviation; Std., standardized; Tau^2^, indicator of statistical heterogeneity; Total, the number of participants; Z, z value; Chi^2^, Chi^2^ test for heterogeneity.

^a^ Horizontal lines indicate the 95% CI of each study; diamond, the pooled estimate with 95% CI; multilevel meta-analysis.

**eFigure 21. Forest plot for sensitivity analysis (quality of pandemic studies) for stress, patients**


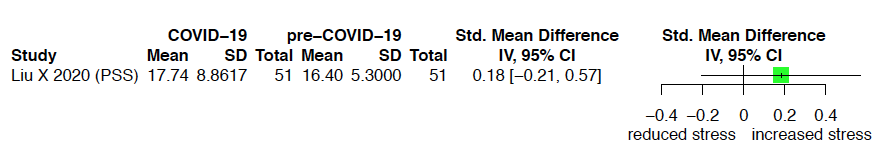


Abbreviations: CI, confidence interval; df, degrees of freedom; I^2^, indicator of statistical heterogeneity; P, p value; SD, standard deviation; Std., standardized; Tau^2^, indicator of statistical heterogeneity; Total, the number of participants; Z, z value; Chi^2^, Chi^2^ test for heterogeneity.

^a^ Horizontal lines indicate the 95% CI of each study; diamond, the pooled estimate with 95% CI; classic random-effects model.

**eFigure 22. Forest plot for sensitivity analysis (quality of pandemic studies) for sleep-related symptoms, general population**


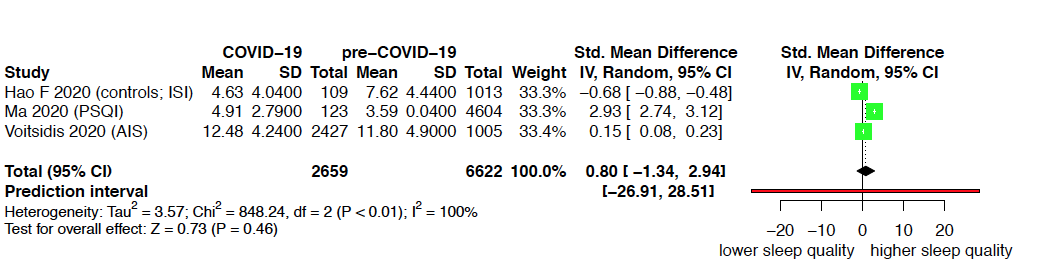


Abbreviations: CI, confidence interval; df, degrees of freedom; I^2^, indicator of statistical heterogeneity; P, p value; SD, standard deviation; Std., standardized; Tau^2^, indicator of statistical heterogeneity; Total, the number of participants; Z, z value; Chi^2^, Chi^2^ test for heterogeneity.

^a^ Horizontal lines indicate the 95% CI of each study; diamond, the pooled estimate with 95% CI; classic random-effects model.

**eFigure 23. Forest plot for sensitivity analysis (quality of pandemic studies) for sleep-related symptoms, healthcare workers**


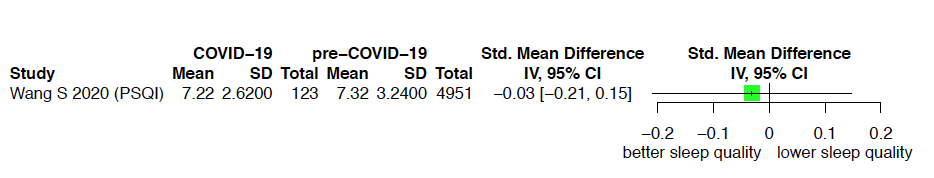


Abbreviations: CI, confidence interval; df, degrees of freedom; I^2^, indicator of statistical heterogeneity; P, p value; SD, standard deviation; Std., standardized; Tau^2^, indicator of statistical heterogeneity; Total, the number of participants; Z, z value; Chi^2^, Chi^2^ test for heterogeneity.

^a^ Horizontal lines indicate the 95% CI of each study; diamond, the pooled estimate with 95% CI; classic random-effects model.

**eFigure 24. Forest plot for sensitivity analysis (quality of pandemic studies) for sleep-related symptoms, patients**


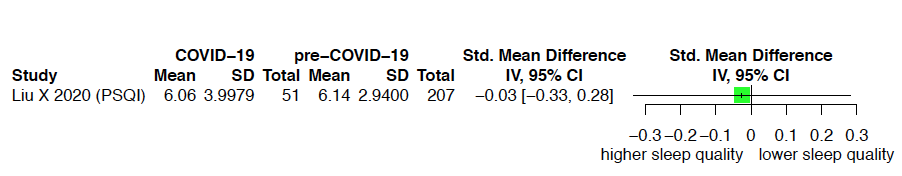


Abbreviations: CI, confidence interval; df, degrees of freedom; I^2^, indicator of statistical heterogeneity; P, p value; SD, standard deviation; Std., standardized; Tau^2^, indicator of statistical heterogeneity; Total, the number of participants; Z, z value; Chi^2^, Chi^2^ test for heterogeneity.

^a^ Horizontal lines indicate the 95% CI of each study; diamond, the pooled estimate with 95% CI; classic random-effects model.

**eFigure 25. Forest plot for sensitivity analysis (level of comparability pandemic vs comparative studies) for anxiety, general population**


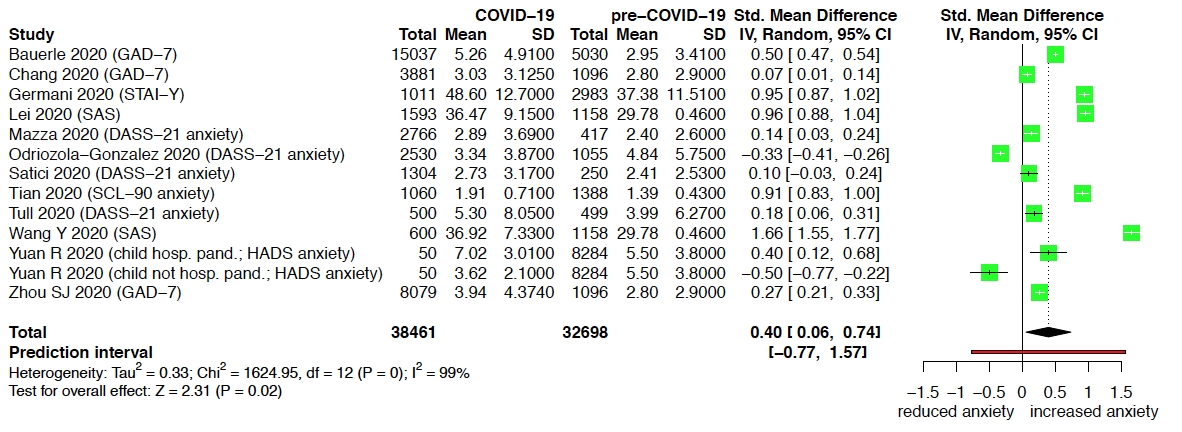


Abbreviations: CI, confidence interval; df, degrees of freedom; I^2^, indicator of statistical heterogeneity; P, p value; SD, standard deviation; Std., standardized; Tau^2^, indicator of statistical heterogeneity; Total, the number of participants; Z, z value; Chi^2^, Chi^2^ test for heterogeneity.

^a^ Horizontal lines indicate the 95% CI of each study; diamond, the pooled estimate with 95% CI; multilevel meta-analysis.

**eFigure 26. Forest plot for sensitivity analysis (level of comparability pandemic vs comparative studies) for anxiety, healthcare workers**


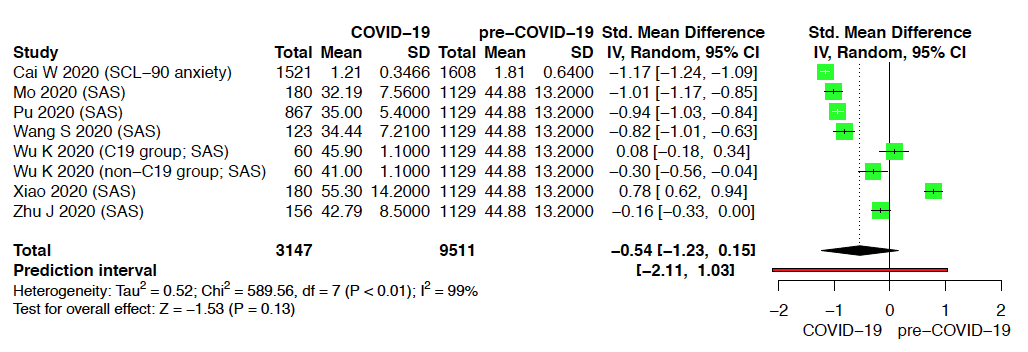


Abbreviations: CI, confidence interval; df, degrees of freedom; I^2^, indicator of statistical heterogeneity; P, p value; SD, standard deviation; Std., standardized; Tau^2^, indicator of statistical heterogeneity; Total, the number of participants; Z, z value; Chi^2^, Chi^2^ test for heterogeneity.

^a^ Horizontal lines indicate the 95% CI of each study; diamond, the pooled estimate with 95% CI; multilevel meta-analysis.

**eFigure 27. Forest plot for sensitivity analysis (level of comparability pandemic vs comparative studies) for anxiety, patients**


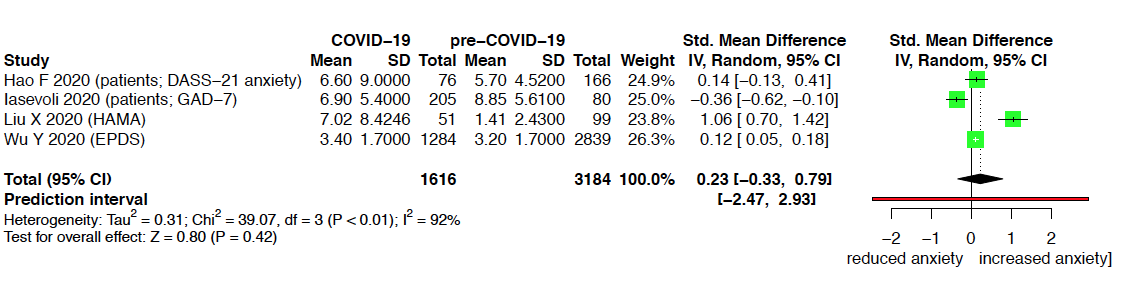


Abbreviations: CI, confidence interval; df, degrees of freedom; I^2^, indicator of statistical heterogeneity; P, p value; SD, standard deviation; Std., standardized; Tau^2^, indicator of statistical heterogeneity; Total, the number of participants; Z, z value; Chi^2^, Chi^2^ test for heterogeneity.

^a^ Horizontal lines indicate the 95% CI of each study; diamond, the pooled estimate with 95% CI; classic random-effects model.

**eFigure 28. Forest plot for sensitivity analysis (level of comparability pandemic vs comparative studies) for depression, general population**


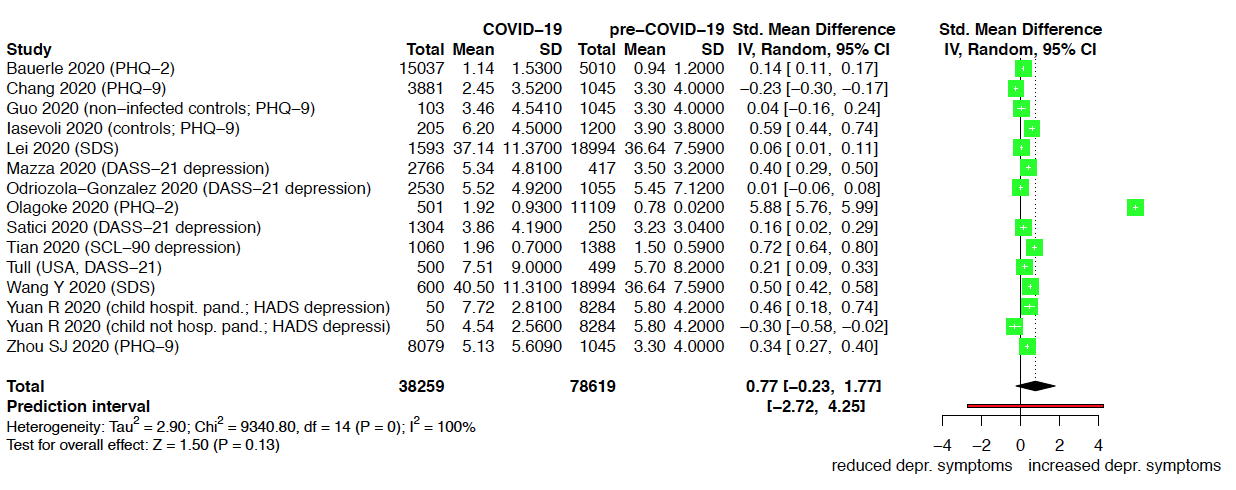


Abbreviations: CI, confidence interval; df, degrees of freedom; I^2^, indicator of statistical heterogeneity; P, p value; SD, standard deviation; Std., standardized; Tau^2^, indicator of statistical heterogeneity; Total, the number of participants; Z, z value; Chi^2^, Chi^2^ test for heterogeneity.

^a^ Horizontal lines indicate the 95% CI of each study; diamond, the pooled estimate with 95% CI; multilevel meta-analysis.

**eFigure 29. Forest plot for sensitivity analysis (level of comparability pandemic vs comparative studies) for depression, healthcare workers**


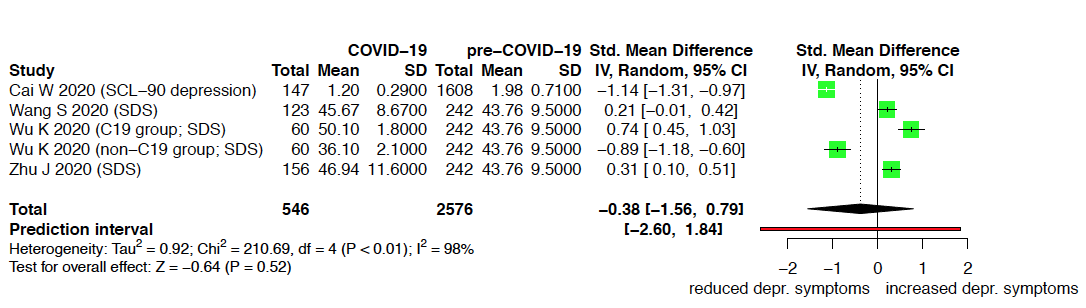


Abbreviations: CI, confidence interval; df, degrees of freedom; I^2^, indicator of statistical heterogeneity; P, p value; SD, standard deviation; Std., standardized; Tau^2^, indicator of statistical heterogeneity; Total, the number of participants; Z, z value; Chi^2^, Chi^2^ test for heterogeneity.

^a^ Horizontal lines indicate the 95% CI of each study; diamond, the pooled estimate with 95% CI; multilevel meta-analysis.

**eFigure 30. Forest plot for sensitivity analysis (level of comparability pandemic vs comparative studies) for depression, patients**


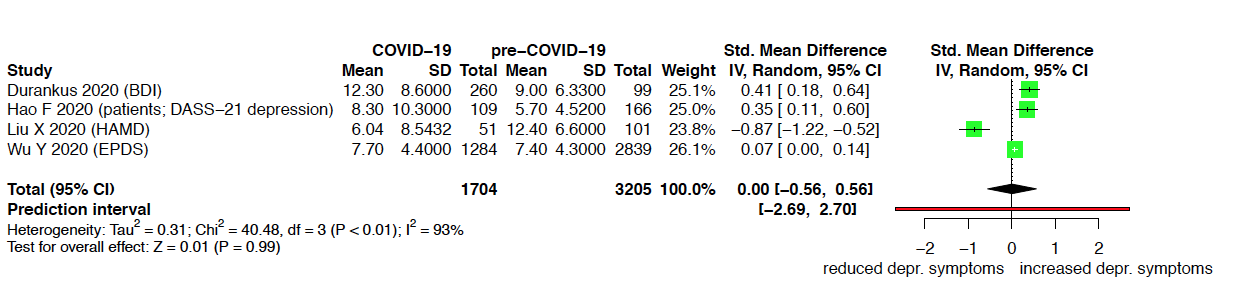


Abbreviations: CI, confidence interval; df, degrees of freedom; I^2^, indicator of statistical heterogeneity; P, p value; SD, standard deviation; Std., standardized; Tau^2^, indicator of statistical heterogeneity; Total, the number of participants; Z, z value; Chi^2^, Chi^2^ test for heterogeneity.

^a^ Horizontal lines indicate the 95% CI of each study; diamond, the pooled estimate with 95% CI; classic random-effects model.

**eFigure 31. Forest plot for sensitivity analysis (level of comparability pandemic vs comparative studies) for stress, general population**


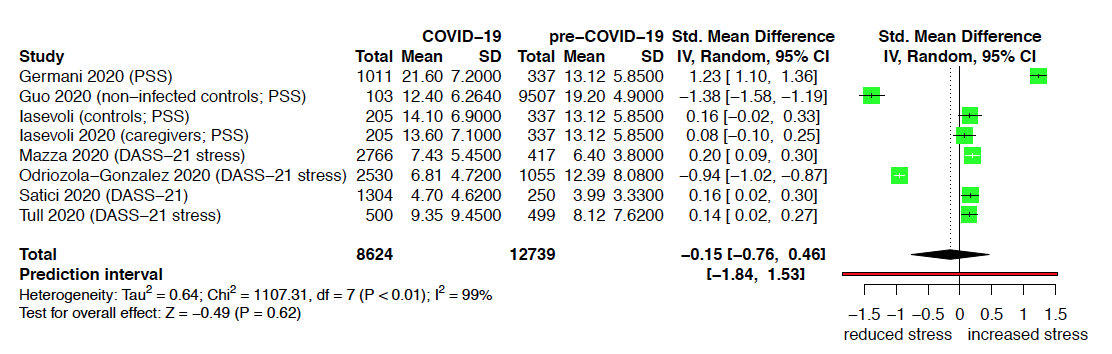


Abbreviations: CI, confidence interval; df, degrees of freedom; I^2^, indicator of statistical heterogeneity; P, p value; SD, standard deviation; Std., standardized; Tau^2^, indicator of statistical heterogeneity; Total, the number of participants; Z, z value; Chi^2^, Chi^2^ test for heterogeneity.

^a^ Horizontal lines indicate the 95% CI of each study; diamond, the pooled estimate with 95% CI; multilevel meta-analysis.

**eFigure 32. Forest plot for sensitivity analysis (level of comparability pandemic vs comparative studies) for stress, patients**


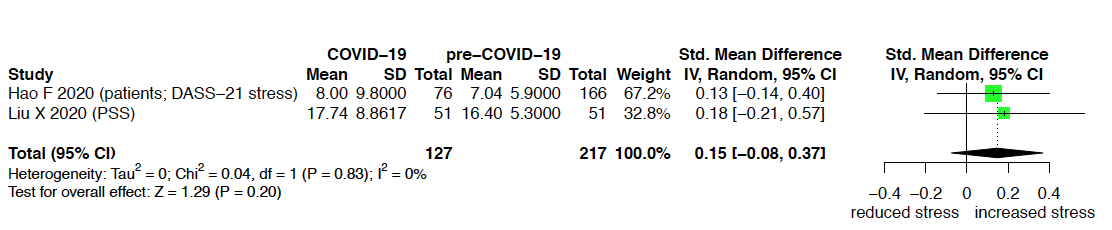


Abbreviations: CI, confidence interval; df, degrees of freedom; I^2^, indicator of statistical heterogeneity; P, p value; SD, standard deviation; Std., standardized; Tau^2^, indicator of statistical heterogeneity; Total, the number of participants; Z, z value; Chi^2^, Chi^2^ test for heterogeneity.

^a^ Horizontal lines indicate the 95% CI of each study; diamond, the pooled estimate with 95% CI; classic random-effects model.

**eFigure 33. Forest plot for sensitivity analysis (level of comparability pandemic vs comparative studies) for sleep-related symptoms, general population**


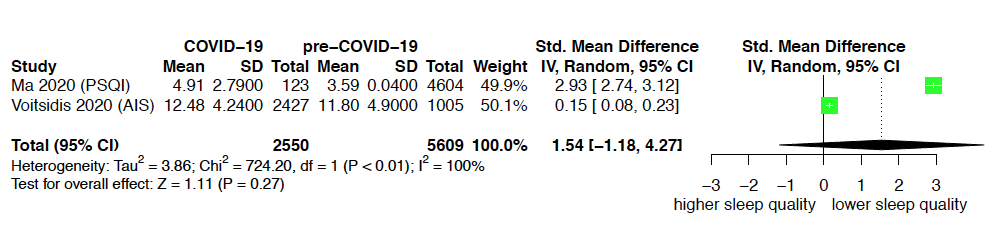


Abbreviations: CI, confidence interval; df, degrees of freedom; I^2^, indicator of statistical heterogeneity; P, p value; SD, standard deviation; Std., standardized; Tau^2^, indicator of statistical heterogeneity; Total, the number of participants; Z, z value; Chi^2^, Chi^2^ test for heterogeneity.

^a^ Horizontal lines indicate the 95% CI of each study; diamond, the pooled estimate with 95% CI; classic random-effects model.

**eFigure 34. Forest plot for sensitivity analysis (level of comparability pandemic vs comparative studies) for sleep-related symptoms, healthcare workers**


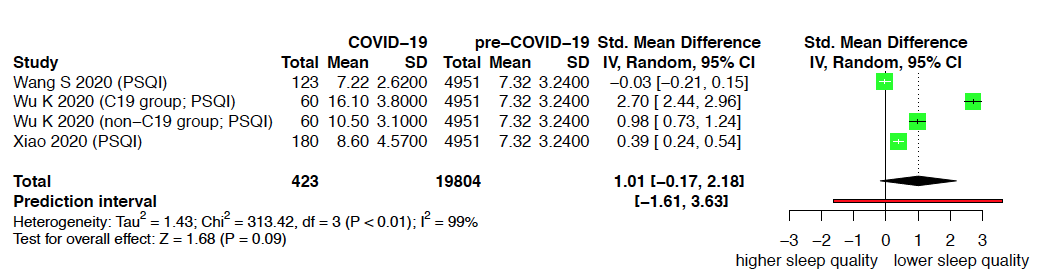


Abbreviations: CI, confidence interval; df, degrees of freedom; I^2^, indicator of statistical heterogeneity; P, p value; SD, standard deviation; Std., standardized; Tau^2^, indicator of statistical heterogeneity; Total, the number of participants; Z, z value; Chi^2^, Chi^2^ test for heterogeneity.

^a^ Horizontal lines indicate the 95% CI of each study; diamond, the pooled estimate with 95% CI; multilevel meta-analysis.

**eFigure 35. Forest plot for sensitivity analysis (level of comparability pandemic vs comparative studies) for sleep-related symptoms, patients**


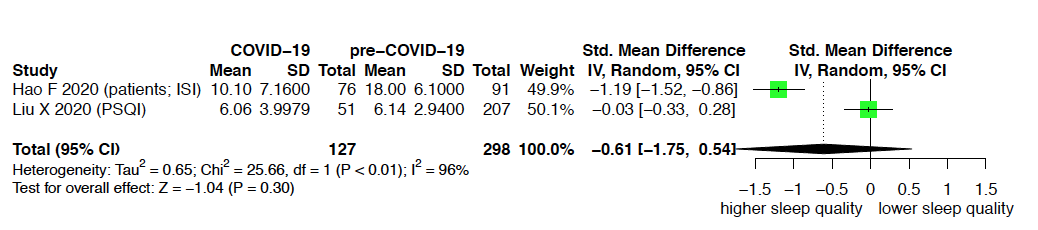


Abbreviations: CI, confidence interval; df, degrees of freedom; I^2^, indicator of statistical heterogeneity; P, p value; SD, standard deviation; Std., standardized; Tau^2^, indicator of statistical heterogeneity; Total, the number of participants; Z, z value; Chi^2^, Chi^2^ test for heterogeneity.

^a^ Horizontal lines indicate the 95% CI of each study; diamond, the pooled estimate with 95% CI; classic random-effects model.

**eResults 3. Detailed results of subgroup analyses**

**Table eResults 3a. Subgroup analyses according to population characteristics**

| **Subgroup** | **Studies (samples)** | **N (pand.)** | **N (comp.)^a^** | **Standardized mean difference (95% CI)** | **Test for subgroup difference^b^** |
| --- | --- | --- | --- | --- | --- |
| **Age** | | | | | |
| **General population** | | | | | |
| **Anxiety** | 23 (26) | 49746 | 132145 (total) | 0.40 (0.15-0.65) | Chi^2^ = 9.5, df = 5 (p=.09) |
| ≤30 years | 7 (8) | 21846 | 20863 | 0.94 (0.43.1.44) |  |
| >30≤35 years | 7 (7) | 7050 | 37815 | 0.11 (-0.42-0.65) |  |
| >35≤40 years | 5 (6) | 4546 | 11810 | 0 (-0.61-0.62) |  |
| >40≤45 years | 2 (2) | 1011 | 14304 | 0.59 (-0.17-1.35) |  |
| multiple groups | 1 (1) | 15037 | 5030 | 0.37 (-0.87-1.61) |  |
| age not specified | 1 (2) | 256 | 5369 | 0.53 (-0.47-1.53) |  |
| **Depression** | 25 (28) | 60213 | 183747 (total) | 0.67 (0.07-1.27) | Chi^2^ = 29.3, df = 5 (p<.001) |
| ≤30 years | 8 (9) | 31812 | 23736 | 1.55 (0.71-2.40) |  |
| >30≤35 years | 8 (8) | 7551 | 66760 | 0.76 (-0.14-1.66) |  |
| >35≤40 years | 5 (6) | 4546 | 17710 | -0.23 (-1.24-0.79) |  |
| >40≤45 years | 2 (2) | 1011 | 14253 | 1.09 (0.10-2.09) |  |
| multiple groups | 1 (1) | 15037 | 5010 | -0.69 (-1.88-0.50) |  |
| age not specified | 1 (2) | 256 | 1539 | 0.24 (-1.95-2.43) |  |
| **Stress** | 11 (13) | 11600 | 67386 (total) | 0.10 (-0.30-0.50) | Chi^2^ = 1043.3, df = 4 (p<.001) |
| ≤30 years | 4 (5) | 6916 | 14850 | 0.59 (-0.19-1.37) |  |
| >30≤35 years | 3 (3) | 3548 | 13625 | -0.71 (-1.49-0.08) |  |
| >35≤40 years | 2 (2) | 623 | 2314 | 0.18 (-1.05-1.42) |  |
| >40≤45 years | 1 (1) | 103 | 9507 | -1.38 (-3.10- -0.07) |  |
| age not specified | 1 (2) | 410 | 337 | -0.52 (-1.32-0.27) |  |
| **Sleep-related symptoms** | 4 (4) | 3332 | 7635 (total) | 0.74 (-1.47-2.96) | Chi^2^ = 188.7, df = 2 (p<.001) |
| ≤30 years | 1 (1) | 2427 | 1005 | 0.15 (-0.25-0.56) |  |
| >30≤35 years | 2 (2) | 782 | 1013 | -0.84 (-1.15- -0.53) |  |
| >35≤40 years | 1 (1) | 123 | 4604 | 2.93 (2.49-3.37) |  |
| **Healthcare workers** | | | | | |
| **Anxiety** | 13 (14) | 5508 | 22204 (total) | -0.08 (-0.66-0.49) | Chi^2^ = 8.7, df = 4 (p=.07) |
| ≤30 years | 2 (2) | 2427 | 2541 | -0.56 (-1.42-0.31) |  |
| >30≤35 years | 8 (9) | 2400 | 2367 | -0.28 (-0.70-0.15) |  |
| >40≤45 years | 1 (1) | 194 | 462 | -0.13 (-1.36-1.10) |  |
| >45 years | 1 (1) | 131 | 5030 | 1.12 (-0.11-2.35) |  |
| several age groups | 1 (1) | 356 | 5030 | 1.04 (-0.18-2.27) |  |
| **Depression** | 7 (8) | 2226 | 4605 (total) | -0.16 (-0.59-0.26) | Chi^2^ = 2.2, df = 1 (p=.14) |
| ≤30 years | 2 (2) | 1053 | 2544 | -0.67 (-1.44-0.11) |  |
| >30≤35 years | 5 (6) | 1173 | 1335 | 0.01 (-0.44-0.46) |  |
| **Stress** | 3 (3) | 1570 | 2454 | 0.49 (-0.60-1.57) | Chi^2^ = 164.2, df = 2 (p<.001) |
| ≤30 years | 1 (1) | 906 | 936 | -0.21 (-0.30- -0.12) |  |
| >30≤ 5 years | 1 (1) | 470 | 936 | 0.11 (0.00-0.22) |  |
| >40≤45 years | 1 (1) | 194 | 582 | 1.06 (0.88-1.23) |  |
| **Sleep-related symptoms** | 4 (5) | 554 | 20024 (total) | 0.83 (-0.14-1.81) | Chi^2^ = 0.3, df = 1 (p=.57) |
| >30≤35 years | 3 (4) | 423 | 4951 | 1.01 (-0.55-2.57) |  |
| >45 years | 1 (1) | 131 | 220 | 0.14 (-2.43-2.71) |  |
| **Patients** | | | | | |
| **Anxiety** | 6 (6) | 1845 | 12458 | 0.31 (-0.07, 0.69) | Chi^2^ = 17.14, df = 4 (p=.002) |
| ≤30 years | 1 (1) | 1284 | 2839 | 0.12 (-0.30-0.54) |  |
| >30≤35 years | 1 (1) | 76 | 166 | 0.14 (-0.35-0.64) |  |
| >40≤45 years | 2 (2) | 154 | 1195 | 0.84 (0.49-1.20) |  |
| >45 years | 1 (1) | 126 | 8178 | 0.25 (-0.20-0.70) |  |
| age not specified | 1 (1) | 205 | 80 | -0.36 (-0.85-0.13) |  |
| **Depression** | 7 (7) | 2138 | 24444 | 0.48 (-0.08-1.04) | Chi^2^ = 3.74, df = 4 (p=.44) |
| ≤30 years | 2 (2) | 1544 | 2938 | 0.24 (-0.83-1.31) |  |
| >30≤35 years | 1 (1) | 109 | 166 | 0.35 (-0.17-1.88) |  |
| >40≤45 years | 2 (2) | 154 | 1146 | -0.09 (-1.17-1.00) |  |
| >45 years | 1 (1) | 126 | 18994 | 1.40 (-1.12-2.91) |  |
| age not specified | 1 (1) | 205 | 1200 | 1.27 (-0.24-2.79) |  |
| **Stress** | 4 (4) | 435 | 10061 | -0.10 (-0.81-0.61) | Chi^2^ = 0.8, df = 2 (p=.68) |
| >30≤35 years | 1 (1) | 76 | 166 | 0.13 (-1.71-1.97) |  |
| >40≤45 years | 2 (2) | 154 | 9558 | -0.49 (-1.80-0.81) |  |
| age not specified | 1 (1) | 205 | 337 | 0.45 (-1.37-2.28) |  |
| **Sleep-related symptoms** | 2 (2) | 127 | 298 | -0.61 (-1.75-0.54) | Chi^2^ = 25.7, df = 1 (p<.001) |
| >30≤35 years | 1 (1) | 76 | 91 | -1.19 (-1.52- -0.86) |  |
| >40≤45 years | 1 (1) | 51 | 207 | -0.03 (-0.33-0.28) |  |
| **Stressor exposure (general population)** | | | | | |
| **Anxiety** | 23 (26)^c^ | 49746 | 132145 (total) | 0.40 (0.15-0.65) | Chi^2^ = 2.8, df = 3 (p=.42) |
| General population | 17 (18) | 33971 | 48173 | 0.51 (0.21-0.80) |  |
| Students | 4 (4) | 15501 | 5134 | 0.24 (-0.38-0.86) |  |
| Others | 1 (1) | 50 | 8284 | -0.50 (-1.77-0.78) |  |
| Special exposure | 3 (3) | 224 | 10438 | 0.27 (-0.47-1.00) |  |
| **Depression** | 25 (28)^c^ | 60213 | 183747 (total) | 0.67 (0.07-1.27) | Chi^2^ = 1.9, df = 3 (p=.60) |
| General population | 19 (20) | 36899 | 79137 | 0.88 (0.20-1.56) |  |
| Students | 4 (4) | 23040 | 2997 | 0.20 (-1.08-1.48) |  |
| Others | 1 (1) | 50 | 8284 | -0.38 (-2.67-1.91) |  |
| Special exposure | 3 (3) | 224 | 10438 | 0.33 (-1.18-1.83) |  |
| **Stress** | 11 (13)^c^ | 11600 | 67386 (total) | 0.10 (-0.30-0.50) | Chi^2^ = 0.12, df = 3 (p=.99) |
| General population | 8 (9) | 7731 | 24218 | 0.01 (-0.60-0.62) |  |
| Students | 2 (2) | 3541 | 1392 | 0.14 (-1.05-1.33) |  |
| Others | 1 (1) | 205 | 337 | -0.10 (-1.77-1.57) |  |
| Special exposure | 1 (1) | 123 | 1815 | 0.22 (-1.48-1.92) |  |
| **Sleep-related symptoms** | 4 (4) | 3332 | 7635 | 0.74 (-1.47-2.96) | Chi^2^ = 14.28, df = 1 (p<.001) |
| Special exposure | 1 (1) | 123 | 4604 | 2.93 (1.53-4.33) |  |
| General population | 3 (3) | 3209 | 2018 | -0.35 (-1.33-0.62) |  |
| **COVID-19 patient contact (healthcare workers)** | | | | | |
| **Anxiety** | 13 (14)^c^ | 5508 | 22204 (total) | -0.08 (-0.66-0.49) | Chi^2^ = 0, df = 1 (p=.95) |
| Low contact risk | 7 (7) | 2818 | 7859 | -0.07 (-0.72-0.58) |  |
| High contact risk | 7 (7) | 2690 | 3670 | -0.09 (-0.83-0.64) |  |
| **Depression** | 7 (8)^1^ | 2226 | 4605 (total) | -0.16 (-0.59-0.26) | Chi^2^ = 1.0, df = 1 (p=.31) |
| Low contact risk | 3 (3) | 1270 | 1335 | -0.50 (-1.23-0.24) |  |
| High contact risk | 5 (5) | 956 | 2786 | -0.06 (-0.70-0.57) |  |
| **Stress** | 3 (3) | 1570 | 2454 (total) | 0.49 (-0.60-1.57) | Chi^2^ = 0, df = 1 (p=1.00) |
| Low contact risk | 2 (2) | 1100 | 1518 | 0.42 (-0.82-1.66) |  |
| High contact risk | 1 (1) | 470 | 936 | 0.42 (-1.22-2.07) |  |
| **Sleep-related symptoms** | 4 (5)^c^ | 554 | 20024 (total) | 0.83 (-0.14-1.81) | Chi^2^ = 0.2, df = 1 (p=.69) |
| Low contact risk | 2 (2) | 191 | 5171 | 0.56 (-1.17-2.29) |  |
| High contact risk | 3 (3) | 363 | 4951 | 1.02 (-0.40-2.43) |  |
| **Subgroup of patients** | | | | | |
| **Anxiety** | 6 (6) | 1845 | 12458 | 0.31 (-0.07, 0.69) | Chi^2^ = 0.3, df = 2 (p=.88) |
| COVID-19 patients | 2 (2) | 229 | 9274 | 0.47 (-0.35-1.29) |  |
| Pregnant women | 1 (1) | 1284 | 2839 | 0.12 (-1.03-1.27) |  |
| Psychiatric patients | 3 (3) | 332 | 345 | 0.27 (-0.41-0.95) |  |
| **Depression** | 7 (7) | 2138 | 24444 | 0.48 (-0.08-1.04) | Chi^2^ = 1.3, df = 2 (p=.51) |
| COVID-19 patients | 2 (2) | 229 | 20039 | 1.03 (-0.08-2.14) |  |
| Pregnant women | 2 (2) | 1544 | 2938 | 0.24 (-0.87-1.35) |  |
| Psychiatric patients | 3 (3) | 365 | 1467 | 0.27 (-0.64-1.18) |  |
| **Stress** | 4 (4) | 435 | 10061 | -0.10 (-0.81-0.61) | Chi^2^ = 44.8, df = 1 (p<.001) |
| COVID-19 patients | 1 (1) | 103 | 9507 | -1.15 (-1.50- -0.80) |  |
| Psychiatric patients | 3 (3) | 332 | 554 | 0.29 (0.06-0.52) |  |

Abbreviations: df, degrees of freedom; p, p value; CI, confidence interval; comp., comparative studies; pand., pandemic studies; N, sample size.

^a^ in meta-analyses with multiply used comparative studies (general population, healthcare workers), the respective study was only counted once to determine the number of control participants.

^b^ Chi^2^=test for subgroup differences.

^c^ Sum of studies does not add up to total number of studies as two samples of the same study were part of different subgroups.

**Table eResults 3b. Subgroup analyses according to pandemic study characteristics**

| **Subgroup** | **Studies (samples)** | **N (pand.)** | **N (comp.)^a^** | **Standardized mean difference (95% CI)** | **Test for subgroup difference^b^** |
| --- | --- | --- | --- | --- | --- |
| **Survey start** | | | | | |
| **General population** | | | | | |
| **Anxiety** | 23 (26)^c^ | 49746 | 132145 | 0.40 (0.15-0.65) | Chi2 = 3.55, df = 4 (p=.47) |
| ≤ 4 weeks | 7 (7) | 8570 | 18665 | 0.65 (0.13-1.17) |  |
| > 4 ≤ 6 weeks | 5 (5) | 2894 | 14310 | -0.08 (-0.79-0.62) |  |
| > 6 ≤ 8 weeks | 8 (9) | 33408 | 32674 | 0.36 (-0.08-0.81) |  |
| > 8 weeks | 1 (1) | 500 | 499 | 0.18 (-1.13-1.49) |  |
| not specified | 3 (4) | 4374 | 10805 | 0.54 (-0.16-1.25) |  |
| **Depression** | 25 (28)^c^ | 60213 | 183747 | 0.67 (0.07-1.27) | Chi^2^ = 10.15, df = 4 (p=.04) |
| ≤ 4 weeks | 8 (8) | 17120 | 37347 | 0.34 (-0.49-1.16) |  |
| > 4 ≤ 6 weeks | 5 (5) | 2894 | 15310 | -0.04 (-1.25-1.17) |  |
| > 6 ≤ 8 weeks | 8 (9) | 34824 | 30710 | 0.51 (-0.29-1.32) |  |
| > 8 weeks | 2 (2) | 1001 | 11608 | 3.04 (1.45-4.64) |  |
| not specified | 3 (4) | 4374 | 10805 | 0.56 (-0.68-1.81) |  |
| **Stress** | 11 (13)^c^ | 11600 | 67386 | 0.10 (-0.30-0.50) | Chi^2^ = 0.31, df = 4 (p=.99) |
| ≤ 4 weeks | 3 (3) | 1436 | 24530 | -0.06 (-1.20-1.09) |  |
| > 4 ≤ 6 weeks | 3 (3) | 1643 | 13208 | -0.36 (-1.76-1.04) |  |
| > 6 ≤ 8 weeks | 4 (5) | 6717 | 1809 | -0.00 (-1.06-1.05) |  |
| > 8 weeks | 1 (1) | 500 | 499 | 0.14 (-1.84-2.13) |  |
| not specified | 1 (1) | 1304 | 250 | 0.16 (-1.82-2.14) |  |
| **Sleep-related symptoms** | 4 (4) | 3332 | 7635 | 0.74 (-1.47-2.96) | Chi^2^ = 188.73, df = 2 (p<.001) |
| ≤ 4 weeks | 1 (1) | 123 | 4604 | 2.93 (2.49-3.37) |  |
| > 4 ≤ 6 weeks | 2 (2) | 782 | 1013 | -0.84 (-1.15- -0.53) |  |
| > 6 ≤ 8 weeks | 1 (1) | 2427 | 1005 | 0.15 (-0.25-0.56) |  |
| **Healthcare workers** | | | | | |
| **Anxiety** | 13 (14) | 5508 | 22204 | -0.08 (-0.66-0.49) | Chi^2^ = 7.91, df = 4 (p=.10) |
| ≤ 4 weeks | 5 (5) | 1835 | 2026 | 0.01 (-0.55-0.57) |  |
| > 4 ≤ 6 weeks | 2 (2) | 374 | 1591 | -0.57 (-1.46-0.32) |  |
| > 6 ≤ 8 weeks | 2 (2) | 435 | 5335 | 0.40 (-0.49-1.29) |  |
| > 8 weeks | 1 (1) | 356 | 5030 | 1.04 (-0.21-2.30) |  |
| not specified | 3 (4) | 2508 | 2737 | -0.59 (-1.22-0.04) |  |
| **Depression** | 7 (8) | 2226 | 4605 | -0.16 (-0.59-0.26) | Chi^2^ = 0.95, df = 2 (p=.62) |
| ≤ 4 weeks | 4 (4) | 1655 | 1178 | -0.03 (-0.75-0.69) |  |
| > 6 ≤ 8 weeks | 1 (1) | 304 | 157 | -0.11 (-1.45-1.23) |  |
| not specified | 2 (3) | 267 | 1850 | -0.51 (-1.32-0.30) |  |
| **Stress** | 3 (3) | 1570 | 2454 | 0.49 (-0.60-1.57) | Chi^2^ = 13.17, df = 1 (p<.001) |
| ≤ 4 weeks | 2 (2) | 1376 | 936 | -0.05 (-0.40-0.30) |  |
| > 4 ≤ 6 weeks | 1 (1) | 194 | 582 | 1.06 (0.57-1.54) |  |
| **Sleep-related symptoms** | 4 (5) | 554 | 20024 | 0.83 (-0.14-1.81) | Chi^2^ = 4.21, df = 2 (p=.12) |
| ≤ 4 weeks | 2 (2) | 303 | 4951 | 0.18 (-1.18-1.54) |  |
| > 6 ≤ 8 weeks | 1 (1) | 131 | 220 | 0.14 (-1.69-1.97) |  |
| not specified | 1 (2) | 120 | 4951 | 1.84 (0.48-3.21) |  |
| **Patients** | | | | | |
| **Anxiety** | 6 (6) | 1845 | 12458 | 0.31 (-0.07-0.69) | Chi^2^ = 4.58, df = 2 (p=.10) |
| ≤ 4 weeks | 3 (3) | 1438 | 4034 | 0.59 (0.15-1.03) |  |
| > 4 ≤ 6 weeks | 2 (2) | 202 | 8344 | 0.20 (-0.34-0.74) |  |
| > 6 ≤ 8 weeks | 1 (1) | 205 | 80 | -0.36 (-1.13-0.42) |  |
| **Depression** | 7 (7) | 2138 | 24444 | 0.48 (-0.08-1.04) | Chi^2^ = 3.08, df = 3 (p=.38) |
| ≤ 4 weeks | 3 (3) | 1438 | 3985 | -0.03 (-0.89-0.82) |  |
| > 4 ≤ 6 weeks | 2 (2) | 235 | 19160 | 0.88 (-0.17-1.92) |  |
| > 6 ≤ 8 weeks | 1 (1) | 205 | 1200 | 1.27 (-0.19-2.74) |  |
| not specified | 1 (2) | 260 | 99 | 0.41 (-1.07-1.89) |  |
| **Stress** | 4 (4) | 435 | 10061 | -0.10 (-0.81-0.61) | Chi^2^ = 0.76, df = 2 (p=.68) |
| ≤ 4 weeks | 2 (2) | 154 | 9558 | -0.49 (-1.80-0.81) |  |
| > 4 ≤ 6 weeks | 1 (1) | 76 | 166 | 0.13 (-1.71-1.97) |  |
| > 6 ≤ 8 weeks | 1 (1) | 205 | 337 | 0.45 (-1.37-2.28) |  |
| **Sleep-related symptoms** | 2 (2) | 127 | 298 | -0.61 (-1.75-0.54) | Chi^2^ = 25.66, df = 1 (p<.001) |
| ≤ 4 weeks | 1 (1) | 51 | 207 | -0.03 (-0.33-0.28) |  |
| > 4 ≤ 6 weeks | 1 (1) | 76 | 91 | -1.19 (-1.52- -0.86) |  |
| **Study conduction in China** | | | | | |
| **General population** | | | | | |
| **Anxiety** | 23 (26) | 49746 | 132145 | 0.40 (0.15-0.65) | Chi^2^ = 0.10, df = 1 (p=.75) |
| China | 12 (14) | 19300 | 26949 | 0.45 (0.06-0.83) |  |
| Non-China | 11 (12) | 30446 | 35700 | 0.36 (-0.02-0.73) |  |
| **Depression** | 25 (28) | 60213 | 183747 | 0.67 (0.07-1.27) | Chi^2^ = 0.60, df = 1 (p=.44) |
| China | 12 (14) | 19300 | 44734 | 0.35 (-0.65-1.36) |  |
| Non-China | 13 (14) | 40913 | 46793 | 0.85 (0.09-1.61) |  |
| **Stress** | 11 (13) | 11600 | 67386 | 0.10 (-0.30-0.50) | Chi^2^ = 0.10, df = 1 (p=.76) |
| China | 5 (6) | 3079 | 24530 | -0.06 (-0.81-0.68) |  |
| Non-China | 6 (7) | 8521 | 2558 | 0.09 (-0.54-0.72) |  |
| **Sleep-related symptoms** | 4 (4) | 3332 | 7635 | 0.74 (-1.47-2.96) | Chi^2^ = 0.07, df = 1 (p=.79) |
| China | 3 (3) | 905 | 5617 | 1.04 (-2.65-4.74) |  |
| Non-China | 1 (1) | 2427 | 1005 | 0.15 (-5.08-5.38) |  |
| **Healthcare workers** | | | | | |
| **Anxiety** | 13 (14) | 5508 | 22204 | -0.08 (-0.66-0.49) | Chi^2^ = 2.84, df = 1 (p=.09) |
| China | 7 (8) | 3147 | 2737 | -0.61 (-1.41-0.19) |  |
| Non-China | 6 (6) | 2361 | 6730 | 0.26 (-0.37-0.90) |  |
| **Depression** | 7 (8) | 2226 | 4605 | -0.16 (-0.59-0.26) | Chi^2^ = 0.08, df = 1 (p=.78) |
| China | 4 (5) | 546 | 1850 | -0.33 (-1.16-0.50) |  |
| Non-China | 3 (3) | 1680 | 1093 | -0.16 (-1.06-0.74) |  |
| **Sleep-related symptoms** | 4 (5) | 554 | 20024 | 0.83 (-0.14-1.81) | Chi^2^ = 0.32, df = 1 (p=.57) |
| China | 3 (4) | 423 | 4951 | 1.01 (-0.55-2.57) |  |
| Non-China | 1 (1) | 131 | 220 | 0.14 (-2.43-2.71) |  |
| **Patients** | | | | | |
| **Anxiety** | 6 (6) | 1845 | 12458 | 0.31 (-0.07, 0.69) | Chi^2^ = 3.35, df = 1 (p=.07) |
| China | 5 (5) | 1640 | 12378 | 0.43 (0.09-0.77) |  |
| Non-China | 1 (1) | 205 | 80 | -0.36 (-1.13-0.42) |  |
| **Depression** | 7 (7) | 2138 | 24444 | 0.48 (-0.08-1.04) | Chi^2^ = 0.62, df = 1 (p=.43) |
| China | 5 (5) | 1673 | 23145 | 0.33 (-0.35-1.02) |  |
| Non-China | 2 (2) | 465 | 1299 | 0.84 (-0.24-1.93) |  |
| **Stress** | 4 (4) | 435 | 10061 | -0.10 (-0.81-0.61) | Chi^2^ = 0.73, df = 1 (p=.39) |
| China | 3 (3) | 230 | 9724 | -0.29 (-1.15-0.57) |  |
| Non-China | 1 (1) | 205 | 337 | 0.45 (-1.02-1.92) |  |
| **Outcome measure** | | | | | |
| **General population** | | | | | |
| **Anxiety** | 23 (26) | 49746 | 132145 | 0.40 (0.15-0.65) | Chi^2^ = 10.7, df = 6 (p=.10) |
| DASS-21 | 9 (10) | 10984 | 17244 | 0.22 (-0.16-0.60) |  |
| GAD-2 | 1 (1) | 3480 | 110 | 0.36 (-0.80-1.51) |  |
| GAD-7 | 6 (7) | 30326 | 8736 | 0.55 (0.10-1.00) |  |
| HADS | 3 (4) | 692 | 31030 | -0.14 (-0.73-0.45) |  |
| SAS | 2 (2) | 2193 | 1158 | 1.31 (0.47-2.15) |  |
| SCL-90 | 1 (1) | 1060 | 1388 | 0.91 (-0.23-2.06) |  |
| STAI-Y | 1 (1) | 1011 | 2983 | 0.95 (-0.19-2.09) |  |
| **Depression** | 25 (28) | 60213 | 183747 | 0.67 (0.07-1.27) | Chi^2^ = 11.46, df = 5 (p=.04) |
| DASS-21 | 9 (10) | 10984 | 17244 | 0.32 (-0.60-1.25) |  |
| HADS | 3 (4) | 692 | 32030 | 0.27 (-1.03-1.57) |  |
| PHQ-2 | 4 (4) | 21445 | 16119 | 3.14 (1.61-4.67) |  |
| PHQ-9 | 6 (7) | 23839 | 5752 | 0.33 (-0.68-1.34) |  |
| SCL-90 | 1 (1) | 1060 | 1388 | 0.72 (-1.61-3.04) |  |
| SDS | 2 (2) | 2193 | 18994 | 0.28 (-1.83-2.39) |  |
| **Stress** | 11 (13) | 11600 | 67386 | 0.10 (-0.30-0.50) | Chi^2^ = 0.16, df = 1 (p=.69) |
| DASS-21 | 8 (9) | 10076 | 17244 | 0.08 (-0.50-0.66) |  |
| PSS | 3 (4) | 1524 | 9844 | -0.14 (-1.05-0.77) |  |
| **Sleep-related symptoms** | 4 (4) | 3332 | 7635 | 0.74 (-1.47-2.96) | Chi^2^ = 188.73, df = 2 (p<.001) |
| AIS | 1 (1) | 2427 | 1005 | 0.15 (-0.25-0.56) |  |
| ISI | 2 (2) | 782 | 1013 | -0.84 (-1.15- -0.53) |  |
| PSQI | 1 (1) | 123 | 4604 | 2.93 (2.49-3.37) |  |
| **Healthcare workers** | | | | | |
| **Anxiety** | 13 (14) | 5508 | 22204 | -0.08 (-0.66-0.49) | Chi^2^ = 2.80, df = 4 (p=.59) |
| DASS-21 | 2 (2) | 1376 | 933 | 0.12 (-1.46-1.70) |  |
| GAD-2 | 1 (1) | 304 | 305 | -0.32 (-2.08-1.45) |  |
| GAD-7 | 3 (3) | 681 | 5492 | 0.54 (-0.63-1.72) |  |
| SAS | 6 (7) | 1626 | 1129 | -0.34 (-1.78-1.10) |  |
| SCL-90 | 1 (1) | 1521 | 1608 | -1.17 (-2.92-0.59) |  |
| **Depression** | 7 (8) | 2226 | 4605 | -0.16 (-0.59-0.26) | Chi^2^ = 2.91, df = 3 (p=.41) |
| DASS-21 | 2 (2) | 1376 | 936 | -0.19 (-1.10-0.72) |  |
| PHQ-2 | 1 (1) | 304 | 157 | -0.11 (-1.33-1.12) |  |
| SCL-90 | 1 (1) | 147 | 1608 | -1.14 (-2.36-0.00) |  |
| SDS | 3 (4) | 399 | 242 | 0.09 (-0.63-0.82) |  |
| **Stress** | 3 (3) | 1570 | 2454 | 0.49 (-0.60-1.57) | Chi^2^ = 13.17, df = 1 (p<.001) |
| DASS-21 | 2 (2) | 1376 | 936 | -0.05 (-0.40-0.30) |  |
| PSS | 1 (1) | 194 | 582 | 1.06 (0.57-1.54) |  |
| **Sleep-related symptoms** | 4 (5) | 554 | 20024 | 0.83 (-0.14-1.81) | Chi^2^ = 0.32, df = 1 (p=.57) |
| ISI | 1 (1) | 131 | 220 | 0.14 (-2.43-2.71) |  |
| PSQI | 3 (4) | 423 | 4951 | 1.01 (-0.55-2.57) |  |
| **Patients** | | | | | |
| **Anxiety** | 6 (6) | 1845 | 12458 | 0.31 (-0.07-0.69) | Chi^2^ = 1.18, df = 4 (p=.88) |
| DASS-21 | 1 (1) | 76 | 166 | 0.14 (-1.31-1.60) |  |
| EPDS-3A | 1 (1) | 1284 | 2839 | 0.12 (-1.32-1.55) |  |
| GAD-7 | 2 (2) | 308 | 1176 | 0.17 (-0.86-1.20) |  |
| HAMA | 1 (1) | 51 | 99 | 1.06 (-0.42-2.53) |  |
| SAS | 1 (1) | 126 | 8178 | 0.25 (-1.19-1.69) |  |
| **Depression** | 7 (7) | 2138 | 24444 | 0.48 (-0.08-1.04) | Chi^2^ = 16.95, df = 5 (p=.005) |
| BDI | 1 (1) | 260 | 99 | 0.41 (-0.45-1.26) |  |
| DASS-21 | 1 (1) | 109 | 166 | 0.35 (-0.35-1.21) |  |
| EDPS | 1 (1) | 1284 | 2839 | 0.07 (-0.76-0.89) |  |
| HAMD | 1 (1) | 51 | 101 | -0.87 (-1.76-0.03) |  |
| PHQ-9 | 2 (2) | 308 | 2245 | 0.97 (0.38-1.57) |  |
| SDS | 1 (1) | 126 | 18994 | 1.40 (0.55-2.24) |  |
| **Stress** | 4 (4) | 435 | 10061 | -0.10 (-0.81-0.61) | Chi^2^ = 0.09, df = 1 (p=.76) |
| DASS-21 | 1 (1) | 76 | 166 | 0.13 (-1.56-1.83) |  |
| PSS | 3 (3) | 359 | 9895 | -0.18 (-1.15-0.80) |  |
| **Sleep-related symptoms** | 2 (2) | 127 | 298 | -0.61 (-1.75-0.54) | Chi^2^ = 25.66, df = 1 (p<.001) |
| ISI | 1 (1) | 76 | 91 | -1.19 (-1.52- -0.86) |  |
| PSQI | 1 (1) | 51 | 207 | -0.03 (-0.33-0.28) |  |
| **Sample size** | | | | | |
| **General population** | | | | | |
| **Anxiety** | 23 (26)^c^ | 49746 | 132145 | 0.40 (0.15-0.65) | Chi^2^ = 1.86, df = 1 (p=.17) |
| <1000 participants | 12 (14) | 4825 | 54175 | 0.24 (-0.10-0.58) |  |
| ≥1000 participants | 12 (12) | 44921 | 28966 | 0.58 (0.22-0.93) |  |
| **Depression** | 25 (28)^c^ | 60213 | 183747 | 0.67 (0.07-1.27) | Chi^2^ = 0.03, df = 1 (p=.86) |
| <1000 participants | 13 (15) | 5326 | 80239 | 0.70 (-0.02-1.42) |  |
| ≥1000 participants | 13 (13) | 54887 | 44535 | 0.63 (-0.12-1.38) |  |
| **Stress** | 11 (13)^c^ | 11600 | 67386 | 0.10 (-0.30-0.50) | Chi^2^ = 2.31, df = 1 (p=.13) |
| <1000 participants | 7 (8) | 2779 | 25366 | -0.27 (-0.87-0.33) |  |
| ≥1000 participants | 5 (5) | 8821 | 15267 | 0.31 (-0.36-0.97) |  |
| **Sleep-related symptoms** | 4 (4) | 3332 | 7635 | 0.74 (-1.47-2.96) | Chi^2^ = 0.07, df = 1 (p=.79) |
| <1000 participants | 3 (3) | 905 | 5617 | 1.04 (-2.65-4.74) |  |
| ≥1000 participants | 1 (1) | 2427 | 1005 | 0.15 (-5.08-5.38) |  |
| **Healthcare workers** | | | | | |
| **Anxiety** | 13 (14) | 5508 | 22204 | -0.08 (-0.66-0.49) | Chi^2^ = 2.83, df = 1 (p=.09) |
| <500 participants | 10 (11) | 2214 | 7859 | 0.09 (-0.45-0.64) |  |
| ≥500 participants | 3 (3) | 3294 | 3670 | -0.57 (-1.34-0.20) |  |
| **Depression** | 7 (8) | 2226 | 4605 | -0.16 (-0.59-0.26) | Chi^2^ = 0.00, df = 1 (p=.96) |
| <500 participants | 6 (7) | 1320 | 2943 | -0.16 (-0.66-0.34) |  |
| ≥500 participants | 1 (1) | 906 | 936 | -0.20 (-1.49-1.08) |  |
| **Stress** | 3 (3) | 1570 | 2454 | 0.49 (-0.60-1.57) | Chi^2^ = 0.79, df = 1 (p=.37) |
| <500 participants | 2 (2) | 664 | 1518 | 0.58 (-0.35-1.51) |  |
| ≥500 participants | 1 (1) | 906 | 936 | 0.02 (-1.20-1.25) |  |
| **Patients** | | | | | |
| **Anxiety** | 6 (6) | 1845 | 12458 | 0.31 (-0.07-0.69) | Chi^2^ = 3.60, df = 1 (p=.06) |
| <200 participants | 4 (4) | 356 | 9539 | 0.52 (0.14-0.90) |  |
| ≥200 participants | 2 (2) | 1489 | 2919 | -0.11 (-0.63-0.42) |  |
| **Depression** | 7 (7) | 2138 | 24444 | 0.48 (-0.08-1.04) | Chi^2^ = 0.09, df = 1 (p=.77) |
| <200 participants | 4 (4) | 389 | 20306 | 0.40 (-0.41-1.21) |  |
| ≥200 participants | 3 (3) | 1749 | 4138 | 0.58 (-0.34-1.51) |  |
| **Stress** | 4 (4) | 435 | 10061 | -0.10 (-0.81-0.61) | Chi^2^ = 0.73, df = 1 (p=.39) |
| <200 participants | 3 (3) | 230 | 9724 | -0.29 (-1.15-0.57) |  |
| ≥200 participants | 1 (1) | 205 | 337 | 0.45 (-1.02-1.92) |  |

Abbreviations: df, degrees of freedom; p, p value; CI, confidence interval; comp., comparative studies; pand., pandemic studies; N, sample size.

^a^ in meta-analyses with multiply used comparative studies (general population, healthcare workers), the respective study was only counted once to determine the number of control participants.

^b^ Chi^2^=test for subgroup differences.

^c^ Sum of studies does not add up to total number of studies as two samples of the same study were part of different subgroups.

**Table eResults 3c. Subgroup analyses according to comparative study characteristics**

| **Subgroup** | **Studies (samples** | **N (pand.)** | **N (comp.)^a^** | **Standardized mean difference (95% CI)** | **Test for subgroup difference^b^** |
| --- | --- | --- | --- | --- | --- |
| **Sample size** | | | | | |
| **General population** | | | | | |
| **Anxiety** | 23 (26) | 49746 | 132145 | 0.40 (0.15-0.65) | Chi^2^ = 0.9, df = 3 (p=.83) |
| ≤500 participants | 4 (4) | 8050 | 1276 | 0.19 (-0.48-0.86) |  |
| >1.000≤5.000 participants | 11 (11) | 22293 | 12868 | 0.54 (0.11-0.97) |  |
| >5.000≤10.000 participants | 3 (5) | 15393 | 13653 | 0.30 (-0.36-0.96) |  |
| >10.000 participants | 5 (6) | 4010 | 34852 | 0.35 (-0.37-1.07) |  |
| **Depression** | 25 (28) | 60213 | 183747 | 0.67 (0.07-1.27) | Chi^2^ = 3.5, df = 4 (p=.48) |
| ≤500 participants | 3 (3) | 4570 | 1166 | 0.25 (-1.32-1.83) |  |
| >500≤1.000 participants | 1 (1) | 8550 | 897 | -0.38 (-3.11-2.35) |  |
| >1.000≤ 5.000 participants | 9 (10) | 19345 | 11215 | 0.43 (-0.52-1.39) |  |
| >5.000≤10.000 participants | 4 (5) | 21044 | 13294 | 0.58 (-1.19-2.35) |  |
| >10.000 participants | 8 (9) | 6704 | 64955 | 1.69 (0.39-2.98) |  |
| **Stress** | 11 (13) | 11600 | 67386 | 0.10 (-0.30-0.50) | Chi^2^ = 8.6, df = 3 (p=.03) |
| ≤500 participants | 5 (6) | 5991 | 1503 | 0.33 (-0.16-0.82) |  |
| >1.000≤5.000 participants | 2 (2) | 2653 | 2870 | -0.37 (-1.21-0.48) |  |
| >5.000≤10.000 participants | 1 (1) | 103 | 9507 | -1.38 (-2.59- -0.19) |  |
| >10.000 participants | 3 (4) | 2853 | 13208 | 0.37 (-0.23-0.97) |  |
| **Healthcare workers** | | | | | |
| **Anxiety** | 13 (14) | 5508 | 22204 | -0.08 (-0.66-0.49) | Chi^2^ = 9.93, df = 3 (p=.02) |
| ≤500 participants | 2 (2) | 498 | 767 | -0.22 (-1.04-0.59) |  |
| >500≤1.000 participants | 2 (2) | 1376 | 933 | 0.12 (-0.74-0.98) |  |
| >1.000≤5.000 participants | 7 (8) | 3147 | 2737 | -0.51 (-1.03-0.01) |  |
| >5.000≤10.000 participants | 2 (2) | 487 | 5030 | 1.08 (0.22-1.95) |  |
| **Depression** | 7 (8) | 2226 | 4605 | -0.16 (-0.59-0.26) | Chi^2^ = 4.3, df = 2 (p=0.12) |
| ≤500 participants | 4 (5) | 703 | 399 | 0.05 (-0.41-0.52) |  |
| >500≤1.000 participants | 2 (2) | 1376 | 936 | -0.19 (-0.91-0.53) |  |
| >1.000≤5.000 participants | 1 (1) | 147 | 1608 | -1.14 (-2.16- -0.11) |  |
| **Sleep-related symptoms** | 4 (5) | 554 | 20024 | 0.83 (-0.14-1.81) | Chi^2^ = 0.3, df = 1 (p=.57) |
| ≤500 participants | 1 (1) | 131 | 220 | 0.14 (-2.43-2.71) |  |
| >1.000≤5.000 participants | 3 (4) | 423 | 4951 | 1.01 (-0.55-2.57) |  |
| **Patients** | | | | | |
| **Anxiety** | 6 (6) | 1845 | 12458 | 0.31 (-0.07, 0.69) | Chi^2^ = 0.1, df = 2 (p=.97) |
| ≤500 participants | 3 (3) | 332 | 345 | 0.27 (-0.44-0.98) |  |
| >1.000≤5.000 participants | 2 (2) | 1387 | 3935 | 0.40 (-0.45-1.25) |  |
| >5.000≤10.000 participants | 1 (1) | 126 | 8178 | 0.25 (-0.95-1.45) |  |
| **Depression** | 7 (7) | 2138 | 24444 | 0.48 (-0.08-1.04) | Chi^2^ = 3.9, df = 2 (p=.14) |
| ≤500 participants | 3 (3) | 420 | 366 | -0.02 (-0.78-0.73) |  |
| >1.000≤5.000 participants | 3 (3) | 1592 | 5084 | 0.67 (-0.07-1.41) |  |
| >10.000 participants | 1 (1) | 126 | 18994 | 1.40 (0.11-2.68) |  |
| **Stress** | 4 (4) | 435 | 10061 | -0.10 (-0.81-0.61) | Chi^2^ = 44.8, df = 1 (p<.001) |
| ≤500 participants | 3 (3) | 332 | 554 | 0.29 (0.06-0.52) |  |
| >5.000≤10.000 participants | 1 (1) | 103 | 9507 | -1.15 (-1.50- -0.80) |  |
| **Publication year** | | | | | |
| **General population** | | | | | |
| **Anxiety** | 23 (26) | 49746 | 132145 | 0.40 (0.15-0.65) | Chi^2^ = 8.0, df = 5 (p=.16) |
| published ≤1 year ago | 2 (2) | 5500 | 3326 | 0.75 (-0.08-1.58) |  |
| published ≤2 years ago6 | 6 (7) | 6076 | 16441 | 0.48 (0.03-0.92) |  |
| published >2≤5 years ago | 5 (6) | 14929 | 9797 | 0.05 (-0.43-0.54) |  |
| published >5≤10 years ago | 4 (4) | 4572 | 23355 | 0.02 (-0.57-0.61) |  |
| published >10 years ago | 5 (6) | 18546 | 7915 | 0.85 (0.37-1.34) |  |
| unclear | 1 (1) | 123 | 1815 | 0.09 (-1.10-1.27) |  |
| **Depression** | 25 (28) | 60213 | 183747 | 0.67 (0.07-1.27) | Chi^2^ = 12.4, df = 5 (p=.03) |
| published ≤1 year ago | 4 (4) | 15643 | 23217 | 0.29 (-0.79-1.36) |  |
| published ≤2 years ago | 5 (6) | 5065 | 13458 | 0.36 (-0.98-1.70) |  |
| published >2≤5 years ago | 3 (4) | 2989 | 10516 | 0.34 (-0.87-1.54) |  |
| published >5≤10 years ago | 9 (10) | 34012 | 29737 | 0.43 (-0.40-1.26) |  |
| published >10 years ago | 2 (2) | 1561 | 12497 | 3.30 (1.74-4.85) |  |
| unclear | 2 (2) | 943 | 21096 | 0.38 (-1.09-1.85) |  |
| **Stress** | 11 (13) | 11600 | 67386 | 0.10 (-0.30-0.50) | Chi^2^ = 11.6, df = 4 (p=.02) |
| published ≤1 year ago | 2 (2) | 2633 | 10562 | -1.16 (-1.96- -0.36) |  |
| published ≤2 years ago | 3 (4) | 2853 | 13208 | 0.37 (-0.20-0.94) |  |
| published >2≤5 years ago | 3 (4) | 4187 | 754 | 0.42 (-0.15-0.98) |  |
| published >5≤10 years ago | 1 (1) | 500 | 499 | 0.14 (-0.99-1.28) |  |
| unclear | 2 (2) | 1427 | 2065 | 0.19 (-0.62-0.99) |  |
| **Sleep-related symptoms** | 4 (4) | 3332 | 7635 | 0.74 (-1.47-2.96) | Chi^2^ = 188.7, df = 2 (p<.001) |
| published ≤2 years ago | 2 (2) | 782 | 1013 | -0.84 (-1.15- -0.53) |  |
| unclear | 1 (1) | 123 | 4604 | 2.93 (2.49-3.37) |  |
| published >5≤10 years ago | 1 (1) | 2427 | 1005 | 0.15 (-0.25-0.56) |  |
| **Healthcare workers** | | | | | |
| **Anxiety** | 13 (14) | 5508 | 22204 | -0.08 (-0.66-0.49) | Chi^2^ = 14.5, df = 3 (p=.002) |
| published ≤1 year ago | 8 (9) | 3002 | 2062 | -0.24 (-0.59-0.11) |  |
| published ≤2 years ago | 1 (1) | 1521 | 1608 | -1.17 (-2.21- -1.13) |  |
| published >2≤5 years ago | 2 (2) | 498 | 767 | -0.22 (-0.96-0.52) |  |
| published >10 years ago | 2 (2) | 487 | 5030 | 1.08 (0.34-1.82) |  |
| **Depression** | 7 (8) | 2226 | 4605 | -0.16 (-0.59-0.26) | Chi^2^ = 4.6, df = 1 (p=.03) |
| published ≤1 year ago | 6 (7) | 2079 | 1335 | -0.02 (-0.38-0.35) |  |
| published ≤2 years ago | 1 (1) | 147 | 1608 | -1.14 (-2.10- -0.10) |  |
| **Stress** | 3 (3) | 1570 | 2454 | 0.49 (-0.60-1.57) | Chi^2^ = 13.2, df = 1 (p<.001) |
| published ≤1 year ago | 2 (2) | 1376 | 936 | -0.05 (-0.40-0.30) |  |
| published ≤2 years ago | 1 (1) | 194 | 582 | 1.06 (0.57-1.54) |  |
| **Patients** | | | | | |
| **Anxiety** | 6 (6) | 1845 | 12458 | 0.31 (-0.07, 0.69) | Chi^2^ = 0.1, df = 2 (p=.94) |
| published ≤1 year ago | 2 (2) | 256 | 179 | 0.34 (-0.52-1.19) |  |
| published >2≤5 years ago | 3 (3) | 305 | 9440 | 0.36 (-0.33-1.05) |  |
| unclear | 1 (1) | 1284 | 2839 | 0.12 (-1.06-1.29) |  |
| **Depression** | 7 (7) | 2138 | 24444 | 0.48 (-0.08-1.04) | Chi^2^ = 17.0, df = 5 (p=.005) |
| published ≤1 year ago | 1 (1) | 126 | 18994 | 1.40 (0.55-2.24) |  |
| published ≤2 years ago | 1 (1) | 260 | 99 | 0.41 (-0.45-1.26) |  |
| published >2≤5 years ago | 1 (1) | 109 | 166 | 0.35 (-0.51-1.21) |  |
| published >5≤10 years ago | 2 (2) | 308 | 2245 | 0.97 (0.38-1.57) |  |
| published >10 years ago | 1 (1) | 51 | 101 | -0.87 (-1.76-0.03) |  |
| unclear | 1 (1) | 1284 | 2839 | 0.07 (-0.76-0.89) |  |
| **Stress** | 4 (4) | 435 | 10061 | -0.10 (-0.81-0.61) | Chi^2^ = 44.8, df = 1 (p<.001) |
| published ≤1 year ago | 1 (1) | 103 | 9507 | -1.15 (-1.50- -0.80) |  |
| published >2≤5 years ago | 3 (3) | 332 | 554 | 0.29 (0.06-0.52) |  |
| **Sleep-related symptoms** | 2 (2) | 127 | 298 | -0.61 (-1.75-0.54) | Chi^2^ = 25.7, df = 1 (p<.001) |
| published >5≤10 years ago | 1 (1) | 76 | 91 | -1.19 (-1.52- -0.86) |  |
| published ≤1 year ago | 1 (1) | 51 | 207 | -0.03 (-0.33-0.28) |  |

Abbreviations: df, degrees of freedom; p, p value; CI, confidence interval; comp., comparative studies; pand., pandemic studies; N, sample size.

^a^ in meta-analyses with multiply used comparative studies (general population, healthcare workers), the respective study was only counted once to determine the number of control participants.

^b^ Chi^2^=test for subgroup differences.

**Table eResults 3d. Subgroup analyses according to relationship of sample sizes between pandemic and prepandemic comparative studies**

| **Subgroup** | **Studies (samples)** | **N (pand.)** | **N (comp.)^a^** | **Standardized mean difference (95% CI)** | **Test for subgroup difference^b^** |
| --- | --- | --- | --- | --- | --- |
| **Relationship of sample sizes** | | | | | |
| **General population** | | | | | |
| **Anxiety** | 23 (26)^c^ | 49746 | 132145 | 0.40 (0.15-0.65) | Chi^2^ = 10.0, df = 3 (p=.02) |
| Ratio ≥2 | 8 (8) | 37128 | 8297 | 0.18 (-0.21-0.57) |  |
| Ratio ≥0.5<2 | 5 (5) | 6723 | 5316 | 1.11 (0.62-1.60) |  |
| Ratio ≥0.1<0.5 | 2 (2) | 1354 | 4085 | 0.21 (-0.57-0.98) |  |
| Ratio <0.1 | 10 (11) | 4541 | 51077 | 0.28 (-0.05-0.61) |  |
| **Depression** | 25 (28) | 60213 | 183747 | 0.67 (0.07-1.27) | Chi^2^ = 4.8, df = 3 (p=.19) |
| Ratio ≥2 | 7 (7) | 42147 | 8674 | 0.02 (-0.90-0.95) |  |
| Ratio ≥0.5<2 | 4 (4) | 8010 | 9168 | 0.31 (-0.84-1.46) |  |
| Ratio ≥0.1<0.5 | 5 (6) | 3238 | 22904 | 0.80 (-0.08-1.69) |  |
| Ratio <0.1 | 9 (11) | 6818 | 75054 | 1.33 (0.41-2.25) |  |
| **Stress** | 11 (13) | 11600 | 67386 | 0.10 (-0.30-0.50) | Chi^2^ = 0.4, df = 2 (p=.84) |
| Ratio ≥2 | 4 (4) | 7611 | 2059 | 0.18 (-0.64-1.01) |  |
| Ratio ≥0.5<2 | 2 (3) | 910 | 836 | -0.12 (-1.13-0.90) |  |
| Ratio <0.1 | 5 (6) | 3079 | 24530 | -0.11 (-0.96-0.73) |  |
| **Sleep-related symptoms** | 4 (4) | 3332 | 7635 | 0.74 (-1.47-2.96) | Chi^2^ = 1335.6, df = 3 (p<.001) |
| Ratio ≥2 | 1 (1) | 2427 | 1005 | 0.15 (0.08-0.23) |  |
| Ratio ≥0.5<2 | 1 (1) | 673 | 1013 | -0.98 (-1.08- -0.87) |  |
| Ratio ≥0.1<0.5 | 1 (1) | 109 | 1013 | -0.68 (-0.88- -0.48) |  |
| Ratio <0.1 | 1 (1) | 123 | 4604 | 2.93 (2.74-3.12) |  |
| **Healthcare workers** | | | | | |
| **Anxiety** | 13 (14) | 5508 | 22204 | -0.08 (-0.66-0.49) | Chi^2^ = 4.2, df = 2 (p=.12) |
| Ratio ≥0.5<2 | 5 (5) | 4068 | 3975 | -0.45 (-1.09-0.19) |  |
| Ratio ≥0.1<0.5 | 5 (5) | 833 | 1591 | 0.03 (-0.68-0.75) |  |
| Ratio <0.1 | 3 (4) | 607 | 6159 | 0.50 (-0.26-1.26) |  |
| **Depression** | 7 (8) | 2226 | 4605 | -0.16 (-0.59-0.26) | Chi^2^ = 3.8, df = 2 (p=.15) |
| Ratio ≥0.5<2 | 5 (5) | 1959 | 1335 | 0 (-0.47-0.47) |  |
| Ratio ≥0.1<0.5 | 1 (2) | 120 | 242 | -0.08 (-0.84-0.69) |  |
| Ratio <0.1 | 1 (1) | 147 | 1608 | -1.14 (-2.19- -0.08) |  |
| **Stress** | 3 (3) | 1570 | 2454 | 0.49 (-0.60-1.57) | Chi^2^ = 13.2, df = 1 (p<.001) |
| Ratio ≥0.5<2 | 2 (2) | 1376 | 936 | -0.05 (-0.40-0.30) |  |
| Ratio ≥0.1<0.5 | 1 (1) | 194 | 582 | 1.06 (0.57-1.54) |  |
| **Sleep-related symptoms** | 4 (5) | 554 | 20024 | 0.83 (-0.14-1.81) | Chi^2^ = 0.32, df = 1 (p=.57) |
| Ratio ≥0.5<2 | 1 (1) | 131 | 220 | 0.14 (-2.43-2.71) |  |
| Ratio <0.1 | 3 (4) | 423 | 4951 | 1.01 (-0.55-2.57) |  |
| **Patients** | | | | | |
| **Anxiety** | 6 (6) | 1845 | 12458 | 0.31 (-0.07, 0.69) | Chi^2^ = 17.7, df = 3 (p<.001) |
| Ratio ≥2 | 1 (1) | 205 | 80 | -0.36 (-0.82-0.11) |  |
| Ratio ≥0.5<2 | 1 (1) | 51 | 99 | 1.06 (0.53-1.59) |  |
| Ratio ≥0.1<0.5 | 2 (2) | 1360 | 3005 | 0.13 (-0.18-0.43) |  |
| Ratio <0.1 | 2 (2) | 229 | 9274 | 0.46 (0.16-0.77) |  |
| **Depression** | 7 (7) | 2138 | 24444 | 0.48 (-0.08-1.04) | Chi^2^ = 3.0, df = 3 (p=.39) |
| Ratio ≥2 | 1 (1) | 260 | 99 | 0.41 (-1.08-1.90) |  |
| Ratio ≥0.5<2 | 2 (2) | 160 | 267 | -0.25 (-1.31-0.81) |  |
| Ratio ≥0.1<0.5 | 2 (2) | 1489 | 4039 | 0.67 (-0.38-1.71) |  |
| Ratio <0.1 | 2 (2) | 229 | 20039 | 1.03 (-0.02-2.08) |  |
| **Stress** | 4 (4) | 435 | 10061 | -0.10 (-0.81-0.61) | Chi^2^ = 64.2, df = 2 (p<.001) |
| Ratio ≥0.5<2 | 2 (2) | 256 | 388 | 0.37 (0.13-0.62) |  |
| Ratio ≥0.1<0.5 | 1 (1) | 76 | 166 | 0.13 (-0.22-0.48) |  |
| Ratio <0.1 | 1 (1) | 103 | 9507 | -1.15 (-1.44- -0.85) |  |
| **Sleep-related symptoms** | 2 (2) | 127 | 298 | -0.61 (-1.75-0.54) | Chi^2^ = 25.7, df = 1 (p<.0001) |
| Ratio ≥0.5<2 | 1 (1) | 76 | 91 | -1.19 (-1.52- -0.86) |  |
| Ratio ≥0.1<0.5 | 1 (1) | 51 | 207 | -0.03 (-0.33-0.28) |  |

Abbreviations: df, degrees of freedom; p, p value; CI, confidence interval; comp., comparative studies; pand., pandemic studies; N, sample size.

^a^ in meta-analyses with multiply used comparative studies (general population, healthcare workers), the respective study was only counted once to determine the number of control participants.

^b^ Chi^2^=test for subgroup differences.

^c^ Sum of studies does not add up to total number of studies as two samples of the same study were part of different subgroups.

**eTable 14. Risk factors in the general population, healthcare workers, and patients across all categories of mental burden^a^**

|  | **Demographic factors** | **Profession and work-related factors** | **Pandemic-specific factors** | **Information/communication related factors** | **Psychosocial factors** | **Health related factors** |
| --- | --- | --- | --- | --- | --- | --- |
| General population | - being female (González-Sanguino^b^, Chang^b, c^, Mazza^b^, Odriozola-González^d^, Özdin^b^, Sakib^e^, Tsipropoulou^e^, Tull^b^, Zhou SJ^b^, Reznik^e^, Voitsidis^e^) ^49,52,63,66,69,72,74,79,82,83,93^; - being male (Wang C^b^)^85^; - being a student (González-Sanguino^b^, Reznik^e^, Lei^e^, Liu S^e^, Olagoke^e^)^52,57,60,72,146^; - being mother (Lauri Korajlija ^e^)^155^; - grandparents alive (Lauri Korajlija^e^)^155^; - lower education (Tsipropoulou^e^, Tian^e^)^81,82^; - higher education (Sutin^b^, Zhang SX^b^)^121,147^; - younger age (Lei^c, e^ , Gao J^b^, Wang H^e^, Wang C^b^) ^50,57,85,86,^; - older age (Lauri Korajlija^f^, Tsipropoulou^c^, ^e^, Tian^e^, Yang H^b^) ^81,82,88,155^; - financial damage (Lei^e^)^57^; - arts, sciences, health sciences, social sciences, research area: arts, health sciences (vs. engineering; Odriozola-González^d^)^66^; - unemployment or changes in employment (Odriozola-González^d^, Olagoke^e^)^66,146^; - no children (Mazza^b^)^63^; - no current relationship (Olagoke^e^, Tan W^b, c^, Lei^e^, Tian^e^)^57,80,81,146^; - being married (Yang H^b^)^88^; - living with someone (González-Sanguino^b^)^52^; - large household size (Wang C^b^)^85^; - financial damage (Lei^e^)^57^; - health profession (Odriozola-González^d^, Wu W^e^, Tian^e^)^66,81,141^; - agricultural worker, enterprise staff, other professions (Tian^e^)^81^; - rural area (Chang^b, c^, Lei^e^)^49,57^; - location: suburb (Chang^b, c^)^49^; - urban area (Özdin^b, c^, Voitsidis^e^)^69,83^; - history of epidemics in the community (Wang H^e^)^86^ | - non-medical students(Chang^b, c^)^49^; - presential job (González-Sanguino^b^, Mazza^b^)^52,63^; - perceived risk of unemployment (Olagoke^e^)^146^; - missing support by colleagues / workplace (Tan W^b^)^80^; - perceived health risk at workplace (Tan W^b, c^)^80^ | - economic worries about COVID-19 (Cao^f^)^48^; - worries/fears and anxiety about relatives or oneself (Cao^b^, Germani†, McKay^f^, Lei^e^, Odriozola-González^d^, Sakib^b^, Satici^b^, Tsipropoulou^f^, Tan W^b^, Voitsidis^b^, Yuan R^e^)^48,51,57,64,66,74,75,80,82,83,89^; - personal/social worries about COVID-19 (Germani^f^, Cao^f^, Wang C^b^, Zhou SJ^b^, Wang H ^b^)^48,51,85,86,93^; - relatives/ loved-ones with COVID-19 (González-Sanguino^b^, Mazza^b^)^52,63^; - COVID-19 related behaviour changes (Harper^f^)^53^; - children hospitalized during pandemic (Yuan R^e^)^89^; - current local outbreak severity (Lei ^e^, Wu W^e^, Yang H^b^, Zhou SJ^b^,)^57,88,93,116^; - stay-at-home order (Tull^b^)^79^; - COVID-19 impact on daily life (Tull^b^)^79^; - own or close person’s quarantine (Lei^e^, Wang C^b^, Ma^e^)^57,62,85^; - close contact to COVID-19 (Ni^b^)^140^; - working in designated COVID-19 hospital (Wu K^e^)^116^; - intention to self-isolate (Bacon^e^)^44^; - feeling that too much unnecessary worry has been made about COVID-19 epidemic (Wang C^b^)^85^; - history of visiting Wuhan (Wang H^b^)^86^ | - negative information concerning epidemic (Chang^b, c^)^49^; - social media exposure (Gao J^b^, Ni^b^)^50,140^; - knowledge about COVID-19^f^(Germani)^51^; - primary source of health information on COVID-19 radio (Wang C^b^)^85^; - time spent on COVID-19 news (via social media) (Hao X^b^, Ni^b^,)^133,140^; - news exposure (Olagoke^b^)^146^; - concerns with media reports related to epidemic (Wang H^e^)^86^; - unawareness of potential contact with COVID-19 (Voitsidis^e^)^83^ | - history of stressful situations (Mazza^b^, Li Y^b^, González-Sanguino^b^, Sutin^b^)^52,58,63,147^; - reward reactivity (Bacon^f^)^44^; - impulsivity (Bacon^f^)^44^; - behavioural inhibition (Bacon^f^)^44^; - fight-flight-freeze-reaction (Bacon^f^)^44^; - level of concern about personal safety (Bacon^f^)^44^; - emotional and behavioural difficulties (Germani^f^, Wang H^b^)^51,86^; - stress(Germani^f^)^51^; - loneliness (González-Sanguino^b^, Voitsidis^b^)^52,83^; - care/harm as a moral foundation(Harper^f^)^53^; - purity/sanctity as a moral foundation (Harper^f^)^53^; - negative affect (Mazza^b^)^63^; - detachment (Mazza^b^)^63^; - disgust propensity and sensitivity (McKay^f^)^64^; - negative appraisal of effect of confinement (Odriozola-González^d^)^66^; - reward reactivity (Bacon^f^)^44^; - cyclothymic temperament (Moccia^b^)^65^; - depressive temperament (Moccia^b^)^65^; - irritable temperament (Moccia^e^)^65^; - anxious temperament (Moccia^b^)^65^; - need for approval (Moccia^b^)^65^; - preoccupation with relationships (Moccia^e^)^65^; - higher school grades (Zhou SJ^b^)^93^; - being religious (Reznik^e^)^72^; - intolerance of uncertainty (Voitsidis^b^)^83^ | - negative attitudes to illness (Bacon^f^)^44^; - reduced perceived health(Gao J^b^, Hao F^b, c^ , Lei^e^, Wang C^b^, Tan W^b^,)^50,57,80,85,132,^; - mental disease /or symptoms (González-Sanguino^b^, Özdin^b^, Bacon^f^, McKay^f^, Sakib^f^, Soraci^f^, Tsipropoulou^f^, Wu K^f^, Voitsidis, Li Y^b^, Germani†, Hao F^b^, Iasevoli^e^)^44,51,52,58,64,69,74,78,82,83,116,132,135^; - physical symptoms (González-Sanguino^b^, Odriozola-González^d^, Tan W^b^, Wang C^b^, Hao F^b^)^52,66,80,85,132^; - vulnerability to COVID-19 (Wang C^b^, Olagoke^b^, Harper^f^)^53,85,146^; - previous (chronic) medical disease (Lauri Korajlija^e^, Özdin*, Mazza^b^, McKay^f^, González-Sanguino^b^, Wang C^b^, Iasevoli^e^)^52,55,63,64,69,85,135^; - COVID-19 diagnosis (González-Sanguino^b^)^52^; - previous/current treatment (Odriozola-González^d^)^66^; - psychoactive medication (Odriozola-González^d^)^66^; - physical concerns (McKay^f^)^64^; - epidemic-related dreams (Wang H^b^)^86^; - (history of) alcohol abuse (Chang^b, c^)^86^; - internet addiction (Li Y ^b^)^58^ |
| Healthcare workers | - being female (Rossi^b^, Yin^b^, Zhang SX^b^, Badahdah^e^)^98,110,119,121^; - urban/suburban practice setting (Khusid^b, c^)^107^; - being an only child (Wang S^b^)^115^; - low education (Zhang C^b^)^120^; - younger age (Sahu^g^)^111^ | - being a nurse/ nonmedical healthcare worker (Tan B^e^, Zhu J^g^, Rossi^b^)^110,114,122^; - concern about professional future (Consolo^f^, Sahu^g^)^103,111^; - current redeployment (Khusid^b, c^)^107^; - professional experience (Pu^e^, Abdessater^b^)^94,109^; - (subjective) workload (Khusid^b, c^, Shacham^b^)^107,112^; - being a general practitioner (Rossi^b^)^110^; - impact on work-life balance (Sahu^g^)^111^ | - concern about infection with COVID-19 (Consolo^f^, Pu^e^, Zhang C^b^, Zhang SX^b^)^103,109,120121^; - concerns about patients (Consolo^f^)^103^; - current local COVID-19 severity (Khusid^b,c^ , Xu J^e^, Abdessater^b^, Wu W^e^)^94,107,118,141^; - concerns about loved ones (Khusid^b, c^)^107^; - colleagues affected by SARS-CoV-2 (Rossi^b^)^110^; - exposure to COVID-19 patients (Abdessater^b^ ,Rossi^b^, Wang S^b^, Yin ^b, f^)^94,110,115,119,^; - working in high risk departments (Pu^e^, Zhang C^b^)^109,120^ | - insatisfaction with perceived psychological support from media (Zhang C^b^)^120^ | - concern about autonomy (Khusid^b, c^)^107^; - doubts about effective disease control (Zhang C^b^)^120^ | - physical symptoms (Chew^b^)^102^; - personal history of infection with COVID-19 (Khusid^b, c^)^107^; - mental disease /or symptoms (Amerio^f^, Wang S^b^, Wu K^f^, Yin^e, f^, Zhu J^b, c^)^97,115,116,119,122^; - previous medical disease (Abdessater^b^)^94^ |
| Patients | - women (Li X^b^)^126^; - unemployment (Wu Y^b^)^127^; - part-time occupation (Wu Y^b^)^127^; - little living area (Wu Y^b^)^127^; - having siblings (Wu Y^b^)^127^ |  | - effect of COVID-19 on psychology (Durankus^b^)^125^; - contact with COVID-19 (Li X^b^)^126^ |  | - social isolation (Durankus^b^)^125^; - perceived poor family support (Wu Y^b^)^127^; - <7h exercise per week (Wu Y^b^)^127^ | - mental disease /or symptoms (Durankus^b^)^125^; - (suspected) COVID-19 (Guo^e^, Zhang J^e^, Liu X^e^)^42,131,143,^; - inflammatory markers in blood (Guo^f^, Liu X^e^)^131,174^; - placenta previa (Wu Y^b^)^127^ |

^a^ Across all categories of mental burden: anxiety, depression, stress, sleep-related symptoms, psychological distress, posttraumatic stress disorder; additional results regarding the frequency of reported risk factors are available on request by the corresponding author.

^b^ regression analysis.

^c^ ambiguous data.

^d^ General Linear Model.

^e^ comparison of means-

^f^ correlation analysis.

^g^ comparison of frequencies.

**eTable 15. Protective factors in the general population, healthcare workers, and patients across all categories of mental burden^a^**

|  | **Demographic factors** | **Profession and work-related factors** | **Pandemic-specific factors** | **Information/communication-related factors** | **Psychosocial factors** | **Health related factors** |
| --- | --- | --- | --- | --- | --- | --- |
| General population | - good economic situation (Cao^b^, González-Sanguino^b^, Tull^b^, Ni^b^, Olagoke^c^, Yang H^b^)^48,52,79,88,140,146^; - living in a big city (Cao^b^)^48^; - rural areas (Gao J^b^)^50^; - living with parents (Cao^b^)^48^; - older age (Chang^b, d^, González-Sanguino^b^, Odriozola-González^e^, Sutin^b^, Tull^b^, Ni^b^, Mazza^b^, Moccia^c^, Zhang SX 2020a^b^)^49,52,63,65,66,79,90,140,147^; - being male (Jahanshahi^b^, Moccia^b^, Wang C^b^,)^54,65,85^; - being single (Gao J^b^)^50^; - not being single (Odriozola-González^e^, Tan W^b^, Wang H^b^, Yang H^b^)^66,80,86,88^; - residence outside Hubei (Gao J^b^)^50^; - residence in Hubei (Yang H^b^)^88^; - having (several) children (González-Sanguino^b^, Jahanshahi^b^)^52,54^; - being retired(González-Sanguino^b^)^52^; - not being a student (Odriozola-González^e^)^66^; - student (vs. unemployed; Jahanshahi^b^)^54^; - higher education (Gao J^b^, Odriozola-González^e^, Olagoke^c^, González-Sanguino^b^)^50,52,66,146^; - not being unemployed (González-Sanguino^b^, Jahanshahi^b^)^52,54^; - distance to epicentre squared (u-shaped, Zhang SX 2020b^b^)^91^; - living with 1 (vs. 2-4, Odriozola-González^d^)^66^ | - non-presential job (González-Sanguino^b, d^, Jahanshahi^b^)^52,54^; - work not paused (Zhang SX 2020a^b^)^90^ | - prevention and control measures (Zhou SJ^b^)^93^; - hygiene behaviour (Tan W^b^, Wang C^b^)^80,85^ | - satisfaction with/level of information on COVID-19 (González-Sanguino^b^, Wang C^b^, Bäuerle^f^, Chang^b, d^, Zhou SJ^b^, Yang H^b^)^45,49,52,85,88,93^; - knowledge about not being infected (Jahanshahi^b^)^54^ | - social support (Cao^f^, González-Sanguino^b^, Ni^b^)^48,52,140^; - horizontal collectivism (Germani^f^)^51^; - sense of belonging (González-Sanguino^b^)^52^; - self-compassion (González-Sanguino^b^)^52^; - spiritual well-being (González-Sanguino^b^)^52^; - positive appraisal for effect of confinement (Odriozola-González^d^)^66^; - optimism (Wang C^b,^ Zhou SJ^b^,)^85,93^; - being a caregiver for a psychiatric patient (Iasevoli^c^)^135^; - trust in governmental actions to face COVID-19 (Bäuerle^f^)^45^; - environment-related factors for quality of life (Harper^f^)^53^; - good family functioning (Li Y^b^)^58^; - positive coping style (Wang H^b^)^86^; - confidence (Moccia^b^)^65^; - discomfort with closeness (Moccia^b^)^65^; - physical exercise (Jahanshahi^b^)^54^; - sense of control (Yang H^b^)^88^ | - physical quality of life (Harper^f^)^53^ |
| Healthcare workers | - older age (Rossi^b^, Badahdah^f^)^98,110,^; - male gender (Khusid^b, d^, Zhu J^b, d^)^107,122^; - being married (Badahdah^c^)^98^ | - work experience (Cai W^c^)^101^; - perceived access to PPE (Khusid^b, d^)^107^; - prior intensive care unit training (Khusid^b, d^)^107^; - perceived professional support (Khusid^b, d^)^107^; - physicians (Zhang C^b^)^120^ | - availability of test (Khusid^b, d^)^107^ |  | - positive coping style (Zhu J^f^)^145^; - social support (Ni^b^)^140^ | - physical health-related quality of life (Amerio^f^)^97^; - mental health-related quality of life (Amerio^f^)^97^ |
| Patients | - higher education (Wu Y^b^)^127^; - economic well-being (Wu Y^b^)^127^ |  |  |  |  | - higher lymphocyte ratio in blood (Liu X^c^)^42^; - concomitant medical diseases (Iasevoli^c^)^135^; - no change, poor or worse self-reported physical health status (Hao F^b, d^)^132^; - physical symptoms (Hao F^b^)^132^; - no drug-resistant epilepsy (Hao X^b, d^)^133^ |

^a^ Across all categories of mental burden: anxiety, depression, stress, sleep-related symptoms, psychological distress, posttraumatic stress disorder; additional results regarding the frequency of reported protective factors are available on request by the corresponding author.

^b^ regression analysis.

^c^ comparison of means.

^d^ ambiguous data.

^e^ General Linear Model.

^f^ correlation analysis.
